# Novel 1,3,5-Triazinyl Aminobenzenesulfonamides Incorporating Aminoalcohol, Aminochalcone and Aminostilbene Structural Motifs as Potent Anti-VRE Agents, and Carbonic Anhydrases I, II, VII, IX, and XII Inhibitors

**DOI:** 10.3390/ijms23010231

**Published:** 2021-12-26

**Authors:** Eva Havránková, Vladimír Garaj, Šárka Mascaretti, Andrea Angeli, Zuzana Soldánová, Miroslav Kemka, Jozef Motyčka, Marie Brázdová, Jozef Csöllei, Josef Jampílek, Claudiu T. Supuran

**Affiliations:** 1 Department of Chemical Drugs, Faculty of Pharmacy, Masaryk University, Palackého 1-3, 612 42 Brno, Czech Republic; csolleij@pharm.muni.cz; 2 Department of Pharmaceutical chemistry, Faculty of Pharmacy, Comenius University, Odbojarov 10, 832 32 Bratislava, Slovakia; garaj1@uniba.sk (V.G.); kemka2@uniba.sk (M.K.); 3 Czech Advanced Technology and Research Institute, Palacky University, Slechtitelu 27, 783 71 Olomouc, Czech Republic; sarka.mascaretti@gmail.com; 4 Section of Farmaceutical and Neutraceutical Sciences, Neurofarba Department, University of Florence, 50019 Sesto Fiorentino, Italy; andrea.angeli@unifi.it; 5 Department of Molecular Pharmacy, Faculty of Pharmacy, Masaryk University, Palackého 1-3, 612 42 Brno, Czech Republic; soldanovaz@pharm.muni.cz (Z.S.); brazdovam@pharm.muni.cz (M.B.); 6 Department of Pharmaceutical Analysis and Nuclear Pharmacy, Faculty of Pharmacy, Comenius University, Odbojarov 10, 832 32 Bratislava, Slovakia; motycka1@uniba.sk; 7 Department of Chemical Biology, Faculty of Science, Palacky University, Slechtitelu 27, 783 71 Olomouc, Czech Republic; josef.jampilek@gmail.com; 8 Institute of Neuroimmunology, Slovak Academy of Sciences, Dúbravska Cesta 9, 845 10 Bratislava, Slovakia

**Keywords:** vancomycin-resistant enterococci, carbonic anhydrase, inhibition, triazine, benzenesulfonamide, stilbene, chalcone

## Abstract

A series of 1,3,5-triazinyl aminobenzenesulfonamides substituted by aminoalcohol, aminostilbene, and aminochalcone structural motifs was synthesized as potential human carbonic anhydrase (hCA) inhibitors. The compounds were evaluated on their inhibition of tumor-associated hCA IX and hCA XII, hCA VII isoenzyme present in the brain, and physiologically important hCA I and hCA II. While the test compounds had only a negligible effect on physiologically important isoenzymes, many of the studied compounds significantly affected the hCA IX isoenzyme. Several compounds showed activity against hCA XII; (*E*)-4-{2-[(4-[(2,3-dihydroxypropyl)amino]-6-[(4-styrylphenyl)amino]-1,3,5-triazin-2-yl)amino]ethyl}benzenesulfonamide (**31**) and (*E*)-4-{2-[(4-[(4-hydroxyphenyl)amino]-6-[(4-styrylphenyl)amino]-1,3,5-triazin-2-yl)amino]ethyl}benzenesulfonamide (**32**) were the most effective inhibitors with K_I_s = 4.4 and 5.9 nM, respectively. In addition, the compounds were tested against vancomycin-resistant *Enterococcus faecalis* (VRE) isolates. (*E*)-4-[2-({4-[(4-cinnamoylphenyl)amino]-6-[(4-hydroxyphenyl)amino]-1,3,5-triazin-2-yl}amino)ethyl]benzenesulfonamide (**21**) (MIC = 26.33 µM) and derivative **32** (MIC range 13.80–55.20 µM) demonstrated the highest activity against all tested strains. The most active compounds were evaluated for their cytotoxicity against the Human Colorectal Tumor Cell Line (HCT116 p53 +/+). Only 4,4’-[(6-chloro-1,3,5-triazin-2,4-diyl)bis(iminomethylene)]dibenzenesulfonamide (**7**) and compound **32** demonstrated an IC_50_ of ca. 6.5 μM; otherwise, the other selected derivatives did not show toxicity at concentrations up to 50 µM. The molecular modeling and docking of active compounds into various hCA isoenzymes, including bacterial carbonic anhydrase, specifically α-CA present in VRE, was performed to try to outline a possible mechanism of selective anti-VRE activity.

## 1. Introduction

According to the WHO, the increasing resistance of microorganisms against clinically used antibiotics is one of the most extensive worldwide problems for public health [1,2]. Therefore, there is an urgent need to identify new targets of action, which allows the development of new, more efficient drugs while bypassing the bacterial resistance mechanisms [3,4]. One of these targets, which have been widely studied in the last few years, but are still underexploited, are bacterial carbonic anhydrases [5].

Carbonic anhydrases (CAs, EC 4.2.1.1.) are metalloenzymes catalyzing the reversible hydration of carbon dioxide to bicarbonate anion and proton, thereby affecting related physiological processes [3,4,6,7]. Inhibition of bacterial CAs leads to the inhibition of growth, growth defects, and makes bacteria vulnerable to host defense mechanisms [4,8,9]. Over the last few years, a number of articles describing the inhibition activity of clinically licensed or new sulfonamides against bacterial CAs presented in *Vibrio cholerae* [10,11,12,13,14], *Mycobacterium tuberculosis* [15,16,17,18], *Burkholderia pseudomallei* [19], *Burkholderia territorii* [7,20], *Escherichia coli* [21,22], and others, were published.

Vancomycin-resistant enterococci (VRE) cause one of the most difficult to treat cases of gastrointestinal tract colonization and systemic infection, with a high percentage of mortality [23,24,25,26]. In these days, only linezolid is an approved antibiotic against VRE, according to the Food and Drug Administration (FDA) and European Medicines Agency (EMA) [23,27]. 

Flaherty’s group recently published an exciting report on sulfonamides with high activity against VRE [23]. The structures of the discussed compounds are based on Acetazolamide as lead structure. Acetazolamide is the inhibitor of the human carbonic anhydrase I (hCA I) isoenzyme, which has been used for decades to treat glaucoma, epilepsy, periodic paralysis, and others [28]. Furthermore, they indirectly proved the presence of bacterial CAs in VRE by studying the interactions of mentioned sulfonamides with the α-CA and γ-CA from *E. faecium* using computational methods. The selected experiments with cell lines were performed both under normal and increased CO_2_ culture conditions to determine whether the compound exhibits cellular activity mediated by the inhibition of CAs. Because the CAs probably present in *Enterococcus* sp. were yet not cloned and characterized, the in vitro tests confirming the inhibition of CAs as a mechanism of action were not performed yet [3,23].

According to the [23], the α-CA present in *E. faecium* has over a 50% similarity and identity in the sequence with the hCA I and hCA II (both belonging to the α-CA family). The same resemblance of similarity and identity in the sequence probably also exists between hCAs and bacterial CAs of another *Enterococcus* sp. Thus, the development of highly active and highly selective compounds towards enterococci is desirable. 

In this work, a new series of 1,3,5-triazinyl aminobenzenesulfonamides, incorporating aminoalcohol, aminostilbene, and aminochalcone structural motifs, were synthesized and evaluated as anti-VRE agents. A number of aminobenzenesulfonamides demonstrated high (in some cases even picomolar) inhibitory activity against various isoenzymes of hCA, such as physiologically important hCA I, hCA II, or tumor-associated hCA IX and hCA XII [29,30,31]. Due to this reason, the inhibitory activity against hCA I, hCA II, hCA VII, hCA IX, and hCA XII was also determined.

## 2. Results and Discussion

### 2.1. Chemistry

**Scheme 1:**
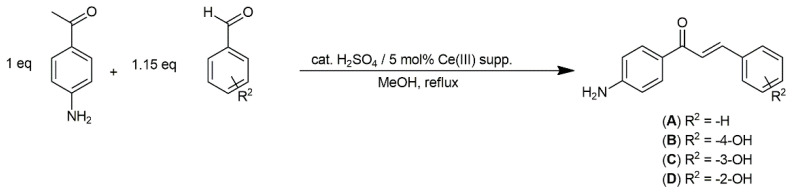
General scheme of synthesis of target chalcone precursors.

Chalcone precursors (**A**–**D**) were prepared from starting aminoacetophenone and corresponding benzaldehyde (Scheme 1). The reaction was catalyzed by a binary catalytic system containing a catalytic amount of H_2_SO_4_ and Ce(III) ions supported on weakly acidic macroporous exchange resin. Cerium(III) ions supported on the cation exchanger already proved their catalytic efficiency in various C-C coupling reactions [32,33,34,35]. The effectiveness of catalysis by system H_2_SO_4_-Ce(III) in terms of reaction time and %yields was compared with catalysis by the catalytic amount of H_2_SO_4_ and catalysis by Ce(III) ions supported on cation exchanger. Binary catalytic system H_2_SO_4_-Ce(III) showed a synergic effect and significantly shortened the reaction time compared to catalysis by H_2_SO_4_ (see Table 1). In the first step of the reaction, Ce(III) ions are probably coordinated to the oxygen of the carbonyl group in aminoacetophenone due to the high oxophility of Ce. This coordination causes a large positive partial charge on carbonyl carbon, which is enhanced by protonation of the amino group (electron-withdrawing effect of -NH_3_^+^). Ce(III) also has the same effect (large positive partial charge) on the oxygen of the carbonyl group in benzaldehyde.

Stilbene precursors (**E**–**H**) were synthesized by a slightly modified Wittig–Horner reaction [36,37]. 

**Scheme 2:**
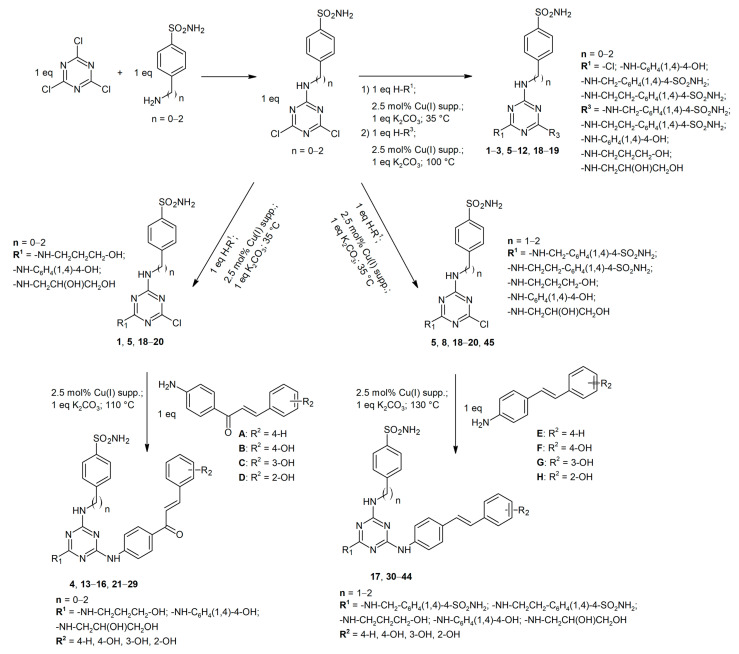
General synthetic scheme for the synthesis of target compounds **1**–**44**.

Target compounds (**1**–**44**) were synthesized according to the methodology published in [38] by a step-by-step nucleophile substitution of chlorine atoms of 4,6-dichloro-1,3,5-triazin-2-yl aminobenzenesulfhonamide. The appropriate starting triazinyl aminobenzenesulfonamide reacted with a nucleophile in the presence of anhydrous potassium carbonate in a molar ratio of 1:1:1. Reactions were catalyzed by Cu(I)-supported on a weakly acidic resin. The substitution of the second or third chlorine atom was controlled by the temperature mode (Scheme 2).

### 2.2. Biological Activity Evaluation

The synthesis and characterization of some of the presented compounds (**1**–**3**, **5**–**15**, **17**–**20**, **22**–**24**, **35**, **37**, **39**, **43**) were previously reported by our group [38,39,40]. However, the targeted biological activities discussed in this article were not determined in the previous articles.

#### 2.2.1. CA Inhibition

The presence of aminobenzenesulfonamide structural moiety in the structure of target compounds **1**–**44** can cause inhibition activity in these compounds against human carbonic anhydrases. The inhibition of physiologically relevant hCA I and hCA II can lead to various undesirable side effects. On the other hand, the inhibition of tumor-associated isoenzymes hCA IX and hCA XII could be a great benefit in tumor treatment. Inhibition of isoenzyme hCA VII, which is presented mostly in the brain, can be a new approach for the treatment of epilepsy [41,42] or chronic neuropathic pain [42,43]. Compounds **1**–**44** were evaluated as potential inhibitors of hCA I, hCA II, hCA VII, hCA IX, and hCA XII. The inhibition activities against individual hCAs were determined by methodology based on stopped-flow assay [44]. The obtained results are shown in Table 2. For completeness, Table 2 also includes the previously published inhibition activities of compounds **2**, **3**, **5**–**12** and 18–19. Values of K_I_s were compared with clinically used sulfonamides as standards—AAZ (acetazolamide), BRZ (brinzolamide), DCP (dichlorphenamide), DZA (dorzolamide), EZA (ethoxzolamide), MZA (methazolamide), and IND (indisulam; canceled in clinical trials).

**Figure 1:**
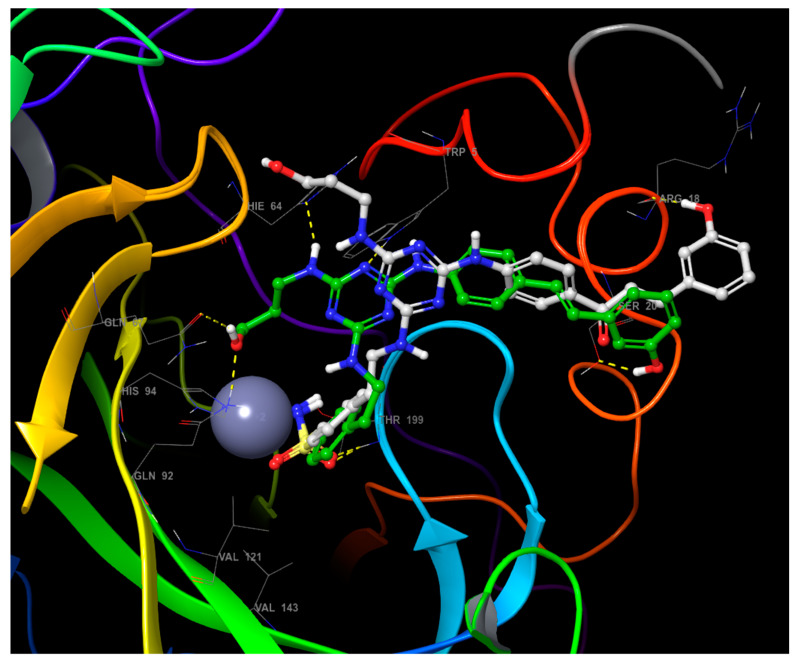
Superposition of ligands **25** (gray carbon atoms) and **37** (green carbon atoms) after docking into the active site of hCA IX.

Based on data presented in Table 2, some general conclusions about relationships between the structure and activity of tested compounds can be made:

- All tested compounds are weak inhibitors of cytosolic isozyme hCA I with K_I_s in a range from 8.5 to >10,000 nM. In general, compounds substituted with stilbenes are better inhibitors of hCA I than compounds containing chalcone substituents. Three stilbene derivatives (**31**, **33**, and **42**) with the highest activity against hCA I with K_I_s in the range of 36.9–48.0 nM have at the terminal benzene core (R^2^) hydrogen (4-H) or hydroxyl functional group (4-OH) in the position para. If the substituents present on the triazine core are very bulky (for example, in a homologous series of compounds **4**, **17**, and **37**), the compounds’ inhibitory activity increases with the increasing number of CH_2_ groups between the triazine core and the benzenesulfonamide structural moiety. If the methylene or ethylene group between the triazine and benzene core is not present, the compound does not fit into the narrowing cavity of the active site due to the steric inherence.
- All compounds presented in this article are very weak inhibitors of physiologically relevant isoenzyme hCA II. In comparison with previously reported compounds **2**, **3**, **5**–**12** and **18**–**19**, chalcone and stilbene derivatives exhibit much lower inhibition activity against hCA II. This is probably caused by the bulkiness of chalcone and stilbene structural moieties. The best inhibition activities of new compounds against hCA II were obtained for chalcone derivatives substituted with 4-OH (**16** with K_I_ = 36.0 nM and **27** with K_I_ = 28.8 nM) or 2-OH (**14** with K_I_s = 35.4 nM) at the terminal benzene core (R^2^). These K_I_s values were comparable with standard **DCP** (K_I_ = 38.0 nM). Substitution 3-OH has a strongly negative effect on the affinity to hCA II. On the other hand, most active stilbene derivatives **40**, **41**, and **17** with K_I_s in the range of 18.9–46.8 nM are substituted at the terminal benzene core with a hydroxyl functional group in position meta (3-OH).
- Selected compounds were tested as potential inhibitors of isoenzyme hCA VII. Chalcone derivatives are, with K_I_s in the range from 25.9 to 810.2 nM, rather weak inhibitors of hCA VII. Exceptions of this are compound **15** with the K_I_ = 25.9 nM comparable with the standard **DCP** (K_I_ = 26.5 nM) and compound **26** (K_I_ = 42.1 nM). Unlike all standards, these compounds show high selectivity over the cytosolic isoenzymes:for compound **15** with K_I_ (hCA II)/K_I_ (hCA VII) = 116.48 and K_I_ (hCA I)/K_I_ (hCA VII) = >386.01, for compound **26** with K_I_ (hCA II)/K_I_ (hCA VII) = 31.71 and K_I_ (hCA I)/K_I_ (hCA VII) = 157.41. All chalcone derivatives with inhibitory activity against hCA VII have common 3-OH substitution at the terminal benzene core of the chalcone structural moiety. From the comparison of the analogue pairs of compounds **15** and **25**, and **14** and **22,** it can be assumed that biological activity and selectivity of chalcone derivatives are negatively affected by the increasing length of the alkyl chain between the 1,3,5-triazine core and the benzenesulfonamide moiety.
- The inhibitory activities of compounds **2**, **3**, **5**–**12** and **18**–**19** against the isoenzyme hCA IX were published and discussed extensively in [39]. For this reason, only the values of K_I_s in the inhibition of hCA IX for chalcone and stilbene derivatives were discussed in this part. Although the structures of chalcone derivatives with potential inhibition activity against isoenzyme hCA IX were selected based on docking (please see the Supplementary Materials), none of the tested compounds showed significant biological activity or selectivity against this isoenzyme. Contrarily, four of tested stilbene derivatives (**17**, **30**, **38,** and **40**) are significantly better inhibitors of hCA IX (K_I_s =12.1–22.2 nM) than all used standards (K_I_s = 24.0–52.0 nM), and the inhibitory activities of another four compounds (**4**, **37**, **41,** and **43**, K_I_s = 25.7–31.1 nM) are comparable to these standards. Interestingly, most of these compounds have in common the hydroxyl group at the position *meta* (3-OH) at the terminal benzene of the stilbene structural moiety. The exception is compound **30,** with the lowest K_I_ = 12.1 nM, where the hydroxyl group is not present at the stilbene moiety. The three of the best inhibitors are also compounds with a very high selectivity over both cytosolic isoenzymes, with values of selectivity for compound **4** K_I_ (hCA II)/K_I_ (hCA IX) = 132.36, K_I_ (hCA I)/K_I_ (hCA IX) = 273.56; for compound **30** K_I_ (hCA II)/K_I_ (hCA IX) = 424.21, K_I_ (hCA I)/K_I_ (hCA IX) = 470.83; and for compound **38** K_I_ (hCA II)/K_I_ (hCA IX) = 256.82, K_I_ (hCA I)/K_I_ (hCA IX) = 388.79. Comparing the best poses of ligands **25** and **37** from molecular docking provides an explanation for the difference in activity between the chalcone and stilbene derivatives (Figure 1). The only difference in the structure of their molecules is the carbonyl group in the chalcone substituent of inactive ligand **25**. Although it is a small structural change in the molecule, the influence on the position and subsequent interactions is significant. While some H-bonds of hydroxyls in distal ends of inactive ligand **25** are made with different residues compared to highly active ligand **37**, some H-bonds of the core part disappeared (with residues His-64, Trp-5), and the carbonyl group creates no H-bond itself.
- Stilbene derivatives were also tested as potential inhibitors of tumor-associated isoenzyme hCA XII. Of the tested compounds, eight (**30**–**34**, **36**, **42,** and **44**) are excellent inhibitors of hCA XII (K_I_s = 4.4–10.0 nM), with biological activity better than (**DCP**, **EZA**), or comparable to (**AAZ**, **IND**, **MZA**), used standards. The structure of most active compounds is quite diverse, containing different aminoalcohole, aminobenzene sulfonamide, and stilbene structural moieties. It is very interesting that stilbene derivatives containing two aminobenzene sulfonamide substituents (**33**, **36,** and **44**), which are very bulky, are among the most active compounds (K_I_s = 6.2–10.0 nM) with good selectivity (K_I_ (hCA II)/K_I_ (hCA VII) = 9.34–25.21). On the other hand, the comparison of the K_I_s values of analogue compounds **4**, **17**, and **37** implies that, with an increasing number of CH_2_ groups in the aminobenzene sulfonamide structural moiety, the biological activity decreases. Thus, it seems that the bulkiness of the molecule is not the only essential factor that affects the biological activity of the tested substances. However, it does significantly affect their selectivity. These observations suggest that, not only does the interaction of the sulfonamide functional group with the active site influence activity and selectivity, but also that the interactions between the rest of the molecule and amino acid residues of the cavity or amino acid residues close to the cavity must have a major effect on the activity and selectivity. In accordance with this statement, it is remarkable that the compounds with excellent selectivity and, at the same time, inhibitory activities comparable with all standards, contain 4’-H-aminostilbene structural moiety **30** (K_I_ = 7.6 nM; K_I_ (hCA II)/K_I_ (hCA XII) = 675.39; K_I_ (hCA I)/K_I_ (hCA XII) =749.60), **32** (K_I_ = 5.9 nM; K_I_ (hCA II)/K_I_ (hCA XII) = 1 269.15; K_I_ (hCA I)/K_I_ (hCA XII) = 15.36), or 2’-OH-aminostilbene structural moiety **34** (K_I_ = 8.5 nM; K_I_ (hCA II)/K_I_ (hCA XII) = 662.59; K_I_ (hCA I)/K_I_ (hCA XII) = 196.71).

#### 2.2.2. VRE Inhibition

All the investigated compounds were tested on their anti-staphylococcal activity against three clinical isolates of methicillin-resistant *Staphylococcus aureus* (MRSA) [49] and *S. aureus* ATCC 29213 as the reference and quality control strain. Surprisingly, these compounds did not show any anti-staphylococcal activity (>256 µg/mL), therefore the data are not reported. On the other hand, some compounds demonstrated a selective ability to inhibit the growth of enterococci. All the compounds were tested against *Enterococcus faecalis* ATCC 29212 as the reference strain and three isolates from American crows of vanA-carrying vancomycin-resistant *E. faecalis* (VRE) [50], and the activities, expressed as minimum inhibitory concentrations (MICs), are listed in Table 3. 

Based on the presented results, it can be stated that derivatives **21** and **32** are the most effective compounds against all tested strains. Other compounds, such as **26**, **9**, and, optionally, **25**, **24**, **7,** and **29,** can also be considered active against at least two of the VRE isolates. It is evident that the activity is associated with substitution at the R^1^ position with the 1-(4-hydroxyphenyl)amino fragment. It appears to be preferred that the compounds be substituted with a bulky lipophilic substituent represented by either a chalcone or stilbene fragment, the subsequent substitution of which rather decreases potency. In this case, it is also advantageous if the linker between the triazine and sulfonamide parts of the molecule is three-membered, which ensures higher conformational flexibility of both parts of the molecule. On the other hand, activity was also observed for derivative **9**, where the bulky and lipophilic aromatic substituent is replaced by a hydrophilic 2,3-hydroxypropylamine chain. It should be noted that this modification of the basic scaffold increases the solubility. On the other hand, similarly substituted potent compounds are limited in amount, therefore it is not possible to decide whether this is a preferred structural modification of anti-VRE active compounds derived from 1,3,5-triazinyl aminobenzenesulfonamides.

Based on the observed results, one may speculate about the specific effectivity against *Enterococcus* sp. It can be hypothesized that anti-VRE activity is caused by the inhibition of bacterial carbonic anhydrase, specifically α-CA, present in VRE. Unfortunately, the pure CA isoenzyme from VRE has not been isolated yet. Therefore, the mechanism of action cannot be experimentally confirmed. However, the selective antibacterial activity could indicate a special mechanism of action associated with the effect on species-specific CA, as discussed below. 

#### 2.2.3. Cytotoxicity Determination against Human Colorectal Tumor Cell Line (HCT116 p53^+/+^)

The compounds with promising inhibitory activity against tumor-associated isoenzymes hCA IX and hCA XII or anti-VRE activity (**7**, **9**, **20**, **21**, **24**, **25**, **26**, **29,** and **32**) were investigated to observe whether they influenced the metabolic activity using the HCT116 p53^+/+^ colorectal tumor cell line after 48 h treatment. Their influence was evaluated as an IC_50_ value (concentration caused a decrease in metabolic activity to 50%). Doxorubicin was used as the positive control. The results are shown in Table 4. Treatment with **9**, **20**, **21**, **24**, **25**, **26,** and **29** in the highest concentration (50 µM) did not significantly decrease the metabolic activity of the investigated tumor cell line. Only compounds **7** and **32** influenced metabolic activity, and their IC_50_ values were determined. Their ability to decrease metabolic activity is lower in comparison with doxorubicin. To conclude these results, compounds **9**, **20**, **21**, **24**, **25**, **26,** and **29** could be discussed as promising antibacterial agents, and compounds **7** and **32** could be further tested for their possible use as anticancer drugs.

### 2.3. Molecular Modeling 

#### 2.3.1. Molecular Docking into hCA IX

A small virtual combinatorial library of 76 compounds was used for virtual screening of hCA IX, and the computed score of the ligands (Supplementary Materials) served as the selection of compounds for synthesis and biological evaluation. The molecules for synthesis were selected from the virtual combinatorial library according to occurrence of substituents on the *s*-triazine core among the best-scoring ligands. For validation, the acetazolamide that co-crystallized with the hCA IX was re-docked in the protein structure with RMSD = 1.57 Å (Supplementary Materials Figure S3), showing good results of the used docking method.

Poses of those ligands with the best inhibition activity against hCA IX (**4**, **17**, **30**, **37**, **38**, **40**, **41**, **43**) were optimized in the active site of a hCA IX crystal structure, and the interaction energies were computed. The values of the binding free energies of top poses obtained from molecular docking (Table 5) are in the range from −55.08 to −90.82 kcal/mol. These values predict strong binding to the hCA IX. The correlation with MIC is poor because of the very narrow range of both computed binding energies and concentrations (taking the expected error of both methods into account).

All poses of the molecules showed an interaction between the deprotonated nitrogen of the sulfonamide group and Zn^2+^ (Supplementary Materials Figure S1). Distance between nitrogen and Zn^2+^ was in a range between 2.15 and 2.17 Å for the complexes with the lowest energy. An oxygen atom of the sulfonamide group created a longer coordination bond with the zinc dication (distance in the range 2.5–2.8 Å), forming a bidentate arrangement. The hydrogen atom of the sulfonamide group served as the H-bond with Thr-199 residue side-chain oxygen. At the same time, the second oxygen of the sulfonamide group served as an acceptor of the H-bond with Thr-199.

**Figure 2:**
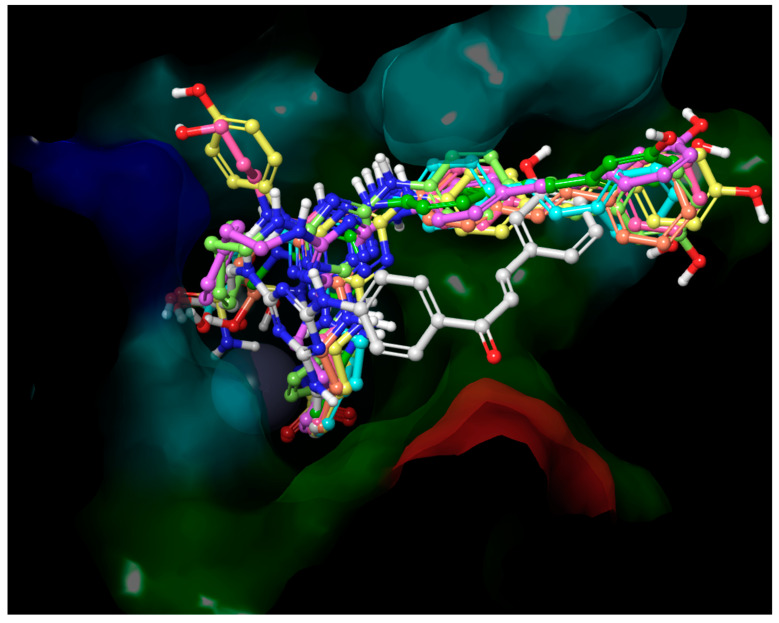
Superposition of the best hCA IX inhibitors docked into the active site of the protein crystal structure. The surface of the active site cavity is colored according to residues properties (green—hydrophobic; light blue—polar; red—negatively charged; dark blue—positively charged). The ligands are distinguished by the color of the carbons as follows: **4**—light gray; **17**—green; **30**—pink; **37**—blue; **38**—orange; **40**—yellow-green; **41**—magenta; **43**—yellow.

**Figure 3:**
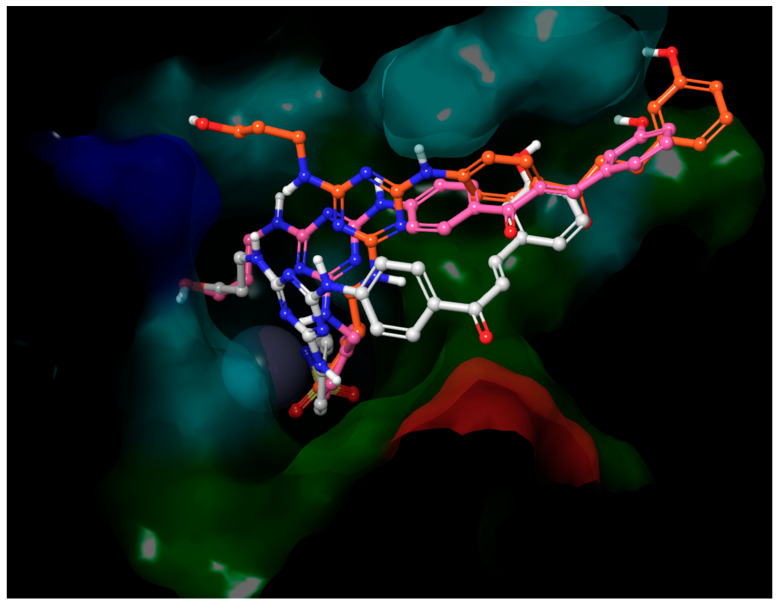
Superposition of the ligands **4, 15, 25** docked into the active site of the hCA IX crystal structure. The surface of the active site cavity is colored according to residues properties (green–hydrophobic; light blue–polar; red–negatively charged; dark blue–positively charged). The ligands are distinguished by the color of the carbons as follows: **4**–light gray; **15**–pink; **25**–orange.

**Figure 4:**
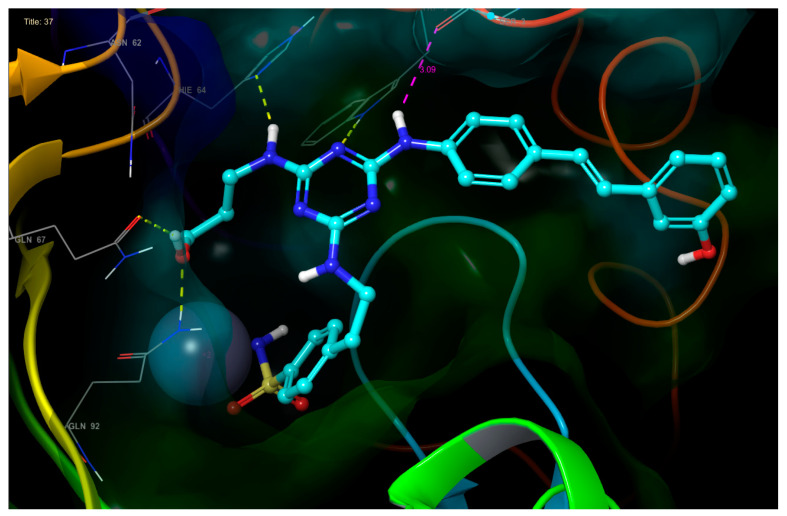
Ligand **37** docked in the hCA IX active site and its H-bonds.

As shown in Figure 2, benzenesulfonamide substituents are oriented to the bottom of the cavity, where they can make coordination bonds with the zinc dication, and all the chalcone/stilbene substituents occupy the same space along the hydrophobic side of the cavity while the third substituent on triazine ring is oriented towards the polar side of the cavity. The arrangement of ligand **4** is slightly different. The reason is a short linker between the benzenesulfonamide and *s*-triazine rings. Ligands **15** and **25** have the same substituents but a longer linker between the benzenesulfonamide and *s*-triazine rings, therefore they accommodated the same volume as other ligands with a longer linker between the rings (Figure 3). While all ligands with longer a linker interact with Trp-5 as H-bond acceptors from indole >NH group, some of them are even H-bond donors to the imidazole of the known proton shuttle residue His-64 by means of *s*-triazine bound >NH group bearing polar substituent or to the Ser-3 carbonyl oxygen (this interaction is weaker because of longer distance) by means of the *s*-triazine bound >NH group bearing a chalcone/stilbene moiety (Figure 4). The short linker prevents such interaction in this set of ligands.

**Figure 5:**
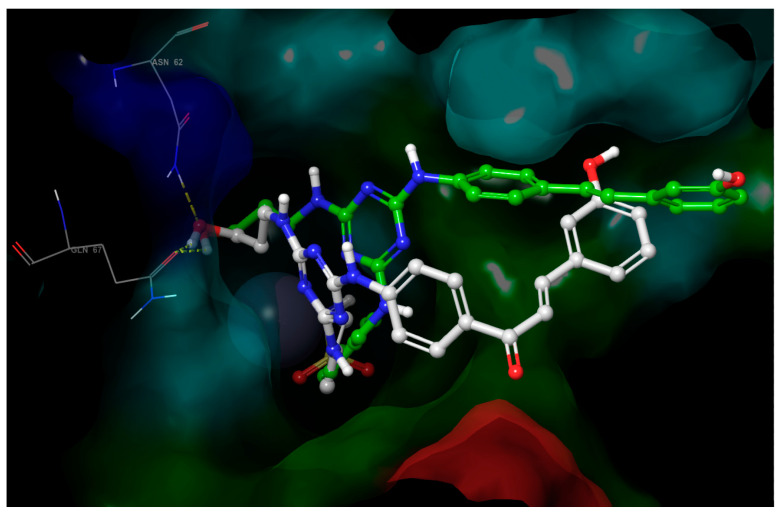
Ligands **4** (gray carbons) and **17** (green carbons) docked in the hCA IX active site. H-bonds with Gln-67 and Asn-62 residues.

**Figure 6:**
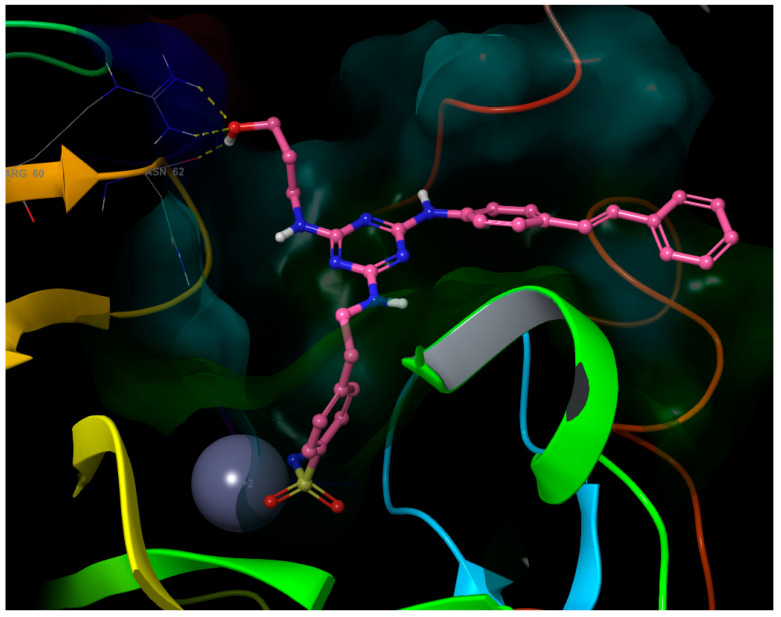
Ligand **30** docked in the hCA IX active site and the H-bonds of hydroxypropyl substituent.

**Figure 7:**
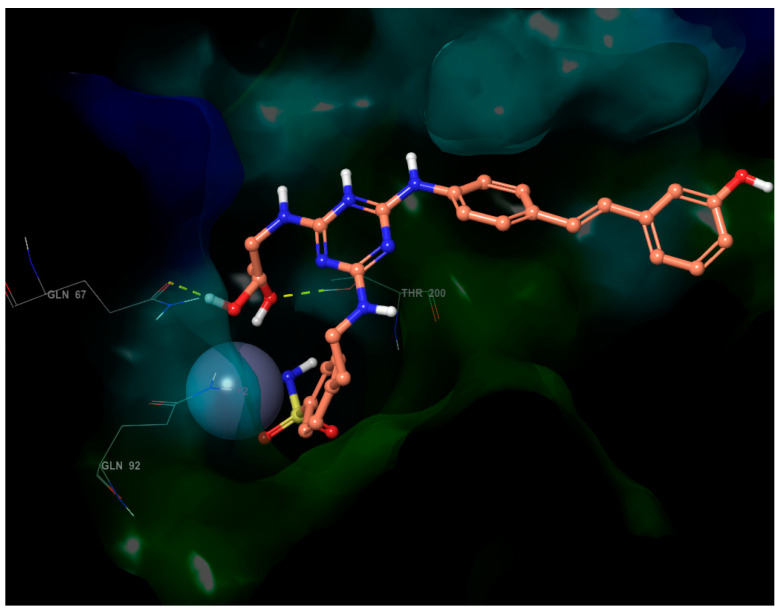
Ligand **38** docked in the hCA IX active site and the H-bonds of the 2,3-dihydroxypropyl substituent.

**Figure 8:**
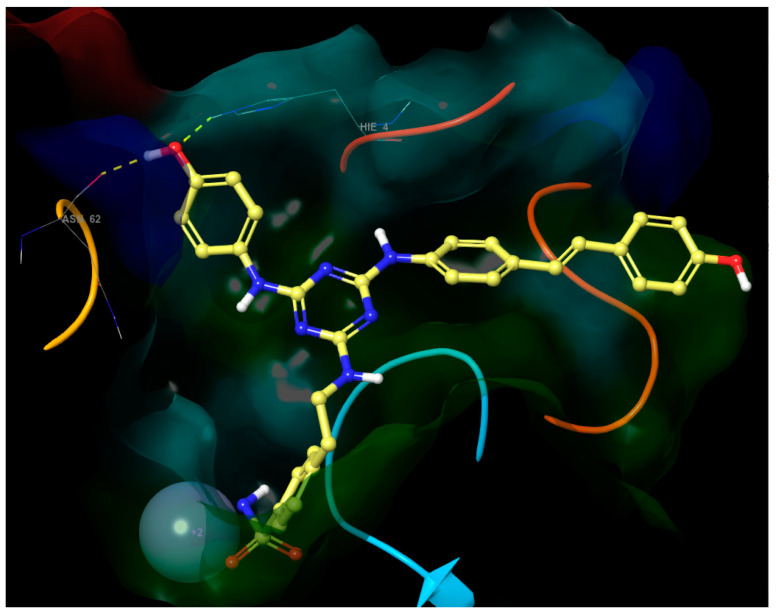
Ligand **43** docked in the hCA IX active site and the H-bonds of phenolic hydroxyl.

The *s*-triazine core bears three substituents responsible for different interactions of the ligand:

- The first substituent is always the benzenesulfonamide moiety with linkers of three different lengths. The coordination of the sulfonamide group to the metal center usually makes up 60% of the interaction energy of the ligands with the carbonic anhydrase [51].
- The second substituent is either stilbene or chalcone, making hydrophobic interaction, which is the weakest interaction, however is significant because of the big surface covered. Most of these substituents bear phenolic hydroxyl at the end, which usually creates H-bonds with bulk water, but in some cases does so with polar groups of amino acid residues at the edge of the cavity (ligand **37** with the hydroxyl of Ser-20; ligand **4** with the carbonyl of Val-19).
- The third substituent is responsible for the H-bond interactions with polar residues, and the polar side of the active site cavity gives a varied offer of polar residues to interact with them. Ligands **4** and **17** with hydroxypropyl substituent (Figure 5) are H-bond acceptors to the Asn-62 side chain and H-bond donors to the Gln-67 side chain. Ligand **30** (Figure 6) shows that also Arg-60 can serve as a H-bond donor and Asn-62 as a H-bond acceptor to the hydroxypropyl substituent. The hydroxypropyl substituent of ligand **37** (Figure 4) is the H-bond acceptor Gln-67 again, but the H-bond donor is Gln-92. The terminal hydroxyl of the polar substituent on ligand **38** interacts the same way as in ligand **37**, and the second hydroxyl on the substituent contributes by accepting the H-bond from Thr-200 (Figure 7). Ligands **40** and **41** with a benzenesulfonamide substituent near the polar side of the cavity use the oxygen of the sulfonamide group as the acceptor of the H-bond from Asn-62, and phenolic hydroxyl on ligand **43** is the H-bond acceptor from the His-4 and H-bond donor to backbone carbonyl of Asn-62 (Figure 8). The aromatic ring of this phenol substituent also allows T-stacking with His-64.

#### 2.3.2. Retrieval of Enterococcal CA Protein Sequence

**Figure 9:**
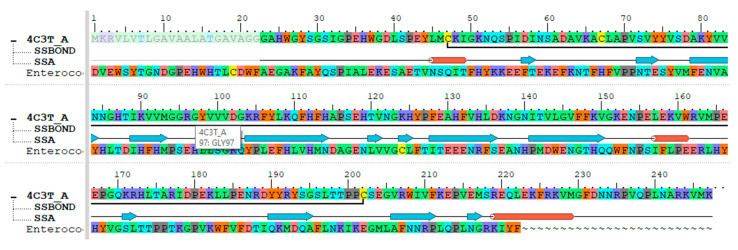
Sequence alignment. In the first row is the sequence of template protein; the sequence of the target protein is in the last row.

There were no resolved 3D structures available for the α-CA protein of *E. faecalis* in PDB yet. In the literature, it was reported that, for homology modeling, the sequence identity between target and template proteins should be 25% or more [52]. The crystal structure of CA from *Thermovibrio ammonificans* (4C3T) was selected with a 31% similarity sequence for the target sequence and 6% for gaps. The sequence alignment is depicted in Figure 9.

#### 2.3.3. 3D Homology Modeling

**Figure 10:**
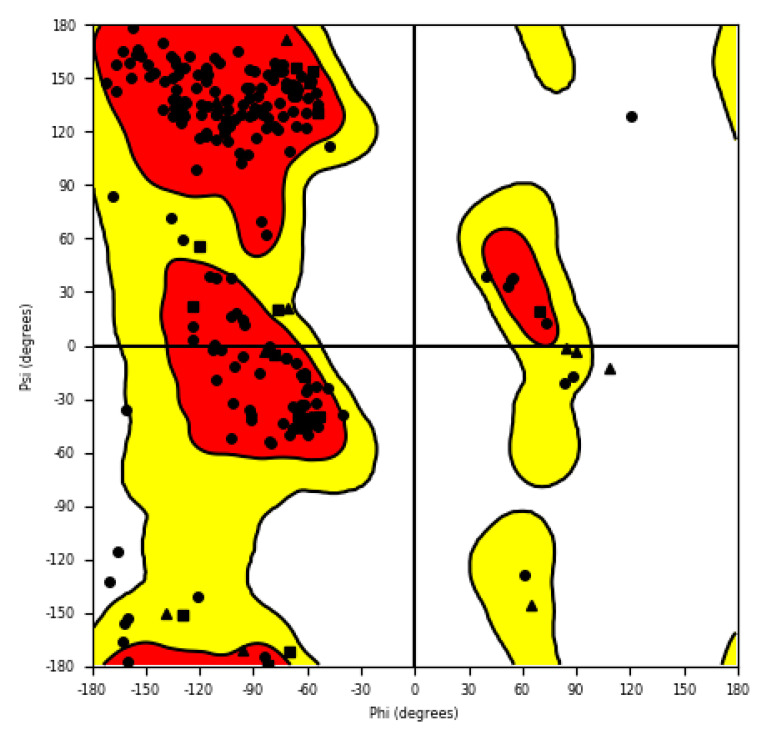
Ramachandran plot of Modelled enterococcal CA protein.

The stereochemical quality of the 3D model was validated by the Ramachandran plot. Figure 10 showed that around 97.8% of the residues were present in the allowed regions (88.4% in the favored region and 9.4% residues in the allowed regions) and that only 2.2% of residues were present in the outlier region, indicating that the quality of the model was good. The plot did not change significantly after MD (Molecular dynamic) simulation, and MD showed the stability of the structure (Figure 16, see Section 4.6.4 in Materials and Methods).

#### 2.3.4. Induced Fit Docking (IFD)

The best poses of all docked molecules show interaction (metal-coordination bond) of deprotonated sulfonamide nitrogen (Lewis base) with Zn^2+^ (Lewis acid) (Supplementary Materials Figure S2). The remaining hydrogen of the sulfonamide group serves as a donor for the H-bond with the backbone carbonyl oxygen of Leu-179 in all cases except ligand **21**. While other ligands have the distance between hydrogen and oxygen atoms 2.4 Å, the top pose of ligand **21** shows a distance of 3.1 Å, and the pose with bidentate binding to Zn^2+^ shows a distance of 2.7 Å. This is too far to create a H-bond and can only be considered as a dipole–dipole interaction.

The enterococcal α-CA has a deep narrow cavity (Figure 12), as is common for the α-class of this enzyme (3D representation of enterococcal carbonic anhydrase created by homology modeling is presented in Supplementary Materials Figure S4). Still, it provides enough space to create a bidentate metal-coordination bond with Zn^2+^ using one of the sulfonamide oxygens, in contrast with the human CAs. The only top pose where the sulfonamide oxygen does not participate in coordination with Zn^2+^ is at compound **21**; however, the scoring algorithm considers the strength of the interaction only with the coulombic term because parametrization is missing for any covalent bonds. There is a high probability that the pose of **21** having a bidentate interaction with Zn^2+^ is the correct one, despite the lower score. A quantum-mechanic computation method is needed to find out.

The second sulfonamide oxygen serves as an acceptor for a very weak H-bond to the backbone nitrogen of Thr-181 with a -H…O- distance of about 2.4 Å and, in the case of **9 R-enantiomer,** also to its hydroxylic group. In the case of the bidentately-binding compound **21,** the H-bond is stronger, with a distance of 1.9 Å.

**Figure 11:**
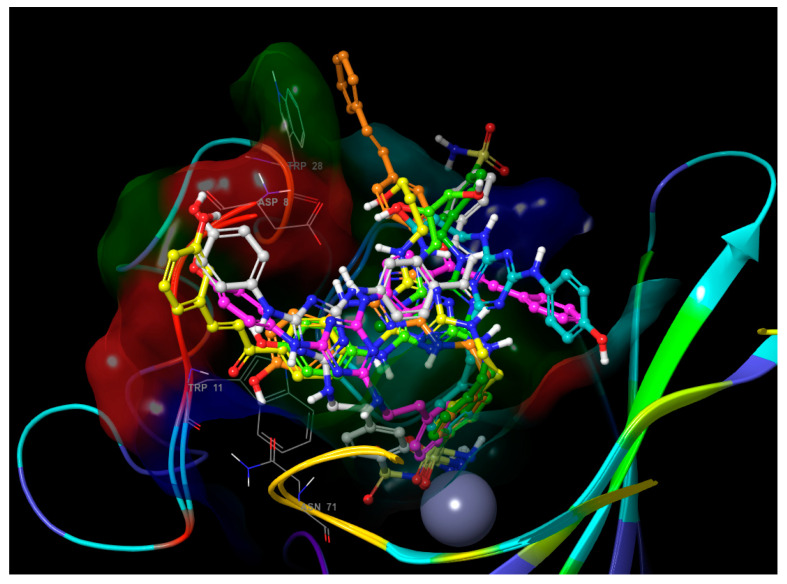
All compounds docked in the active site of the enterococcal α-CA homology model. The colors of carbon atoms for compounds are: **7,** green; **9-S,** yellow-green; **25,** yellow**; 32,** orange. For compound **9-R,** teal; For compound **21** with bidentate binding mode, magenta; For compound **21** with monodentate binding mode, gray. Color of other elements: hydrogen—white, nitrogen—blue, oxygen—red, sulfur—yellow, Zn^2+^—gray-blue sphere.

All compounds have the benzenesulfonamide moiety binding to Zn^2+^ in the same position except compound **21** (Figure 11).

**Figure 12:**
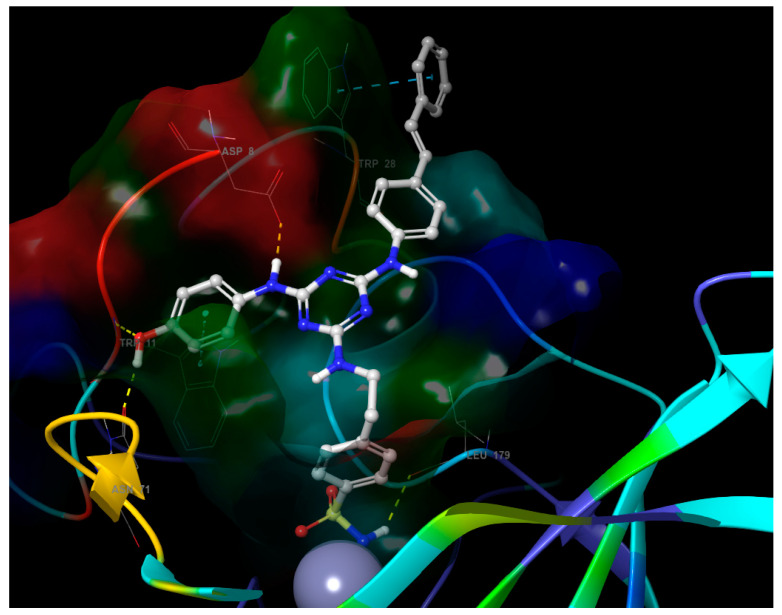
Compound **32** is docked in the active site of enterococcal α-CA homology model. Color of atoms: hydrogen—white, carbon—gray, nitrogen—blue, oxygen—red and sulfur—yellow, Zn^2+^—gray-blue sphere. Intermolecular interactions: H-bond—yellow dashed line, π-π stacking—light blue dashed line. Color of the surface of the binding site: hydrophobic residues—green, polar residues–light blue, negatively charged residues—red, positively charged residues—dark blue.

Ligands **7**, **9-S**, **25**, and **32** are in contact with the surface of the part of the active site of the cavity created by loop 179–185 (Figure 11). Ligand **9-R** shifted a little more to the β-sheet, and ligand **21** shifted a little more to loop 8–13. The most active compound, **32,** creates no H-bonds nor salt bridges with loop 179–185 except hydrophobic interactions (Figure 12). These are supplemented with the π–π stacking of terminal aromatic rings with Trp-28 and Trp-11 and H-bonds of p-aminophenol substituent with Asp-8, Trp-11, and Asn-71. The second most active ligand, **7,** creates with loop 179–185 besides the hydrophobic interactions, as well as with the H-bond, the carbonyl oxygen of Thr-184, and the protonated primary amine group of Lys-185, showing the importance of the second sulfonamide group attached to the aromatic ring which interacts with the aromatic system of Trp-28 through weak T-stacking. 

**Figure 13:**
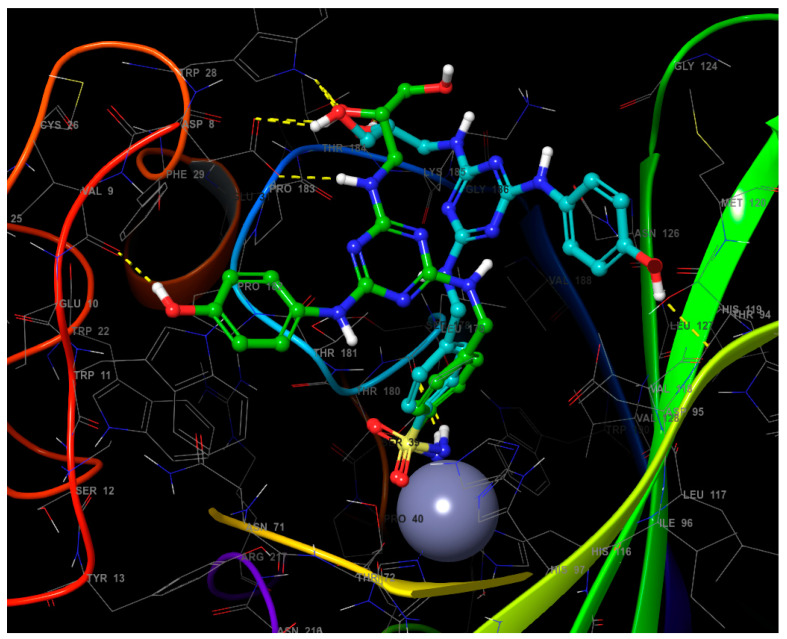
Both enantiomers of compound **9** are docked in the active site of the enterococcal α-CA homology model. The color of carbon atoms for compounds **9-S** is green and is teal for compound **9-R**. Color of other elements: hydrogen—white, nitrogen—blue, oxygen—red, sulfur—yellow, Zn^2+^—gray-blue sphere. Intermolecular interactions: H-bond—yellow dashed line.

When comparing the S- and R-enantiomers of ligand **9** (Figure 13, Supplementary Materials Figure S2), benzenesulfonamide moieties are perfectly overlayed (RMSD = 0.3 Å). The second and last have the H-bond of the chiral hydroxyl group with Trp-28 in common. The difference in the torsional angles of the middle bond of the link between benzenesulfonamide and *s*-triazine is 115°, making the phenol group interact on opposite sides of the active site cavity. Despite the different positions of the disubstituted *s*-triazine moieties, the number of H-bonds of both enantiomers is similar, therefore the affinity can be expected to be similar as well.

From our observation, we conclude that an increase in the inhibition activity of **32** can be achieved by substitution of the stilbene aromatic ring near *s*-triazine with an acidic functional group which would create a salt bridge with the primary ammonium group of Lys-185. 

#### 2.3.5. Binding Energy Calculation

The values of the binding free energies of top poses obtained from the IFD output (Table 6) are in the range from −51.59 to −75.35 kcal/mol. These values predict strong binding to the enterococcal α-CA. The correlation with MIC is poor because of the very narrow range of both computed binding energies and concentrations (taking expected error of both methods into account), and other features may have an impact, e.g., penetration through a bacterial cell wall.

The S-enantiomer of compound **9** has computed a stronger binding affinity, although the chirality does not seem to play an important role in this part of the structure, as the R-enantiomer seems to belong to the group with the best compounds, according to the computed energies and scores.

#### 2.3.6. Prediction of ADMET and Fluorescence

According to the predictions acquired from QikProp (Table 7), we can assume that the studied compounds would not reach the CNS. The most active compound, **32,** has good oral absorption. The lower oral bioavailability of **7** and **9** is associated with a higher hydrophilicity, making those compounds more soluble in water. Caution needs to be taken because of the high predicted affinity to the Human ether-a-go-go-related gene (HERG). QikProp identified the acceptor carbonyl as a reactive group, which can explain the high experimentally measured toxicity of all chalcones. The probable metabolism would include the oxidative hydroxylation of phenyl rings in para- and meta-positions, the oxidation of hydroxyls on aliphatic carbons to carboxylic acids in the case of primary alcohols, and the ketones in the case of secondary alcohols.

**Figure 14:**
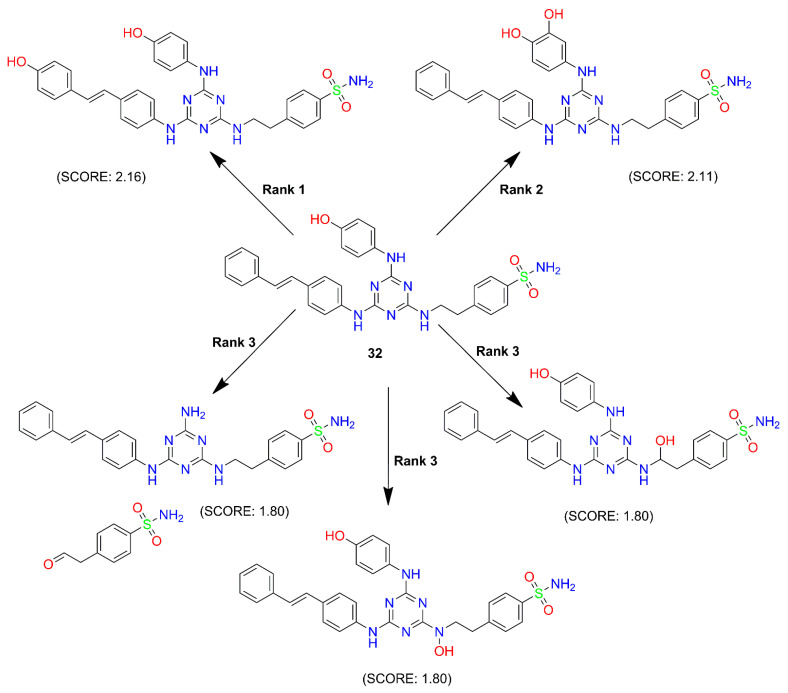
Metabolism of **32** according to algorithm GLORY.

According to GLORY, additional metabolic pathways are the hydroxylation of nitrogen or the neighboring carbon of the link between the *s*-triazine ring and benzenesulfonamide and the cleavage of the whole substituent from the *s*-triazine ring (Figure 14).

The predictions of ChemFLuo (Table 8) show that all compounds except **9** could be used for fluorescent labeling of the enzyme with a green light.

## 3. Conclusions

A series of forty-four 1,3,5-triazinyl aminobenzenesulfonamides was prepared as potential carbonic anhydrase (hCA) inhibitors. All tested compounds are weak inhibitors of physiological isoenzyme hCA I and II. All the chalcone derivatives substituted with 3-OH demonstrated inhibitory activity against hCA VII; compounds 15 and 26 showed the highest activity and selectivity. On the other hand, stilbene derivatives, e.g., molecules 31 and 32, showed to be good inhibitors of isoenzyme hCA XII. Activity against VRE isolates is associated with substitution with the 1-(4-hydroxyphenyl)amino fragment and the stilbene fragment; compounds 21 and 32 demonstrated the highest activity against all tested strains. The most active compounds were evaluated for their cytotoxicity against HCT116 p53^+/+^, and, except for derivatives 7 and 32, showed no toxic effect up to a concentration of 50 μM; i.e., compounds 7 and 32 could be further tested for their possible use as anticancer drugs. In addition, compound 32 has the potential of a multi-target compound to act against both colorectal tumor and enterococcus. The most promising anti-VRE agent 21 exhibited no cytotoxic activity and could be discussed as a promising antibacterial agent. The molecular modeling and docking of active compounds into various hCA isoenzymes, including bacterial carbonic anhydrase, specifically α-CA present in VRE, was performed and outlined in a possible mechanism of selective anti-VRE activity. Thus, it can be stated that the selected derivatives described here deserve further attention and a deeper investigation of their biological properties.

## 4. Materials and Methods

### 4.1. General Informations

All reagents were purchased from commercial suppliers (Sigma–Aldrich, Darmstadt, Germany) and used as supplied without further purification. Commercially unavailable 4-aminobenzaldehyde, X-hydroxybenzylchlorides, (*E*)-X-(4-aminostyryl)phenol, and (*E*)-1-(4-aminophenyl)-3-(X-hydroxyphenyl)prop-2-en-1-one were synthesized according to the literature. Used catalytic systems of Ce(III)-supported on weakly acidic resin and Cu(I)-supported on a weakly acidic resin were purchased from Entwick Chemicals (Entwick Chemicals, Brno, Czech Republic; The company is no longer in operation)

All the reactions were monitored by TLC performed on precoated Silica gel 60 F254 plates (Merck, Darmstadt, Germany). For compounds (**1**, **2**, **3**, **5**–**12**, **18**–**20**), the methanol was used as an eluent; UV light (254 and 356 nm) and ninhydrin reagent were used for the detection of spots at 180 °C. For compounds (**4**, **13**–**17**, **21**–**44**), methanol: dichloromethane = 1:1 was used as an eluent; UV light (254 and 356 nm), iodine, and ninhydrin reagent were used for the detection of spots at 180 °C. NMR spectra were recorded on a DRX 500 Avance (Bruker Biospin, Billerica, MA, USA) spectrometer using TMS as an internal standard. The FTIR spectra were obtained on an Alpha II FTIR Spectrometer (Bruker, Billerica, MA, USA) equipped with the ATR module. Melting points (uncorrected) were recorded on Kofler’s block Boetius Rapido PHMK 79/2106 (Wagetechnik, Dresden, Germany) with a temperature gradient 4 °C/min.

### 4.2. General Synthetic Procedures

#### 4.2.1. General Method for Synthesis of Chalcones

4-Aminoacetophenone (1 eq; 10 mmol) was dissolved in 20 mL of methanol. Then, 1.15 eq (11.5 mmol) of benzaldehyde, 4-hydroxybenzaldehyde, 3-hydroxybenzaldehyde, or 2-hydroxybenzaldehyde was added. Finally, a catalytic amount of H_2_SO_4_ and 5 mol% of Ce(III) ions supported on the weakly acidic resin were added, and the reaction mixture was refluxed until the disappearance of starting aminoacetophenone. The reaction was monitored by TLC: eluent n-hexane:ethyl-acetate = 1:1, UV light (254 and 356 nm), and ninhydrin reagent (at 180 °C) were used for the detection of spots. After the completion of the reaction, the catalyst was filtered off. The reaction mixture was concentrated on a rotary vacuum evaporator to 1/3 of the volume. The crude product was precipitated with cold water and filtered off. The precipitate was suspended in a minimal amount of cold water and added to a dropwise saturated solution of NaHCO_3_ until it reached pH 7–8. The solid was filtered again and washed with cold water until it reached a neutral pH. The crude product was purified as follows: solid was dissolved in hot ethanol, then water was added until the first turbidity. The mixture was placed into the fridge (5–7 °C) overnight, and the clear product was filtered.

Chalcones prepared by Claisen–Schmidt reaction have an exclusively *trans* configuration [53,54,55]. Derivatives **A**, **B**, **C**, and **D** were *trans* configuration confirmed by the presence of two doublets in area 7.7 and 7.5 ppm with characteristic interaction constant *J* = 15.5 Hz [55] at ^1^H-NMR spectra. 

*(E)-1-(4-Aminophenyl)-3-phenylprop-2-en-1-one* (**A**): 

Ocher solid; yield 88%; mp 156–157 °C. IR (ATR): 3336, 3321 (NH_2_), 3041, 1656 (C=O), 1594 (CH=CH). ^1^H-NMR (500 MHz, DMSO-*d_6_*): δ 7.94 (2H, d, *J* = 7.1 Hz, Ar-*H*), 7.77 (1H, d, *J* = 15.7 Hz, C*H*=CH-CO), 7.63 (1H, d, *J* = 15.7 Hz, CH=C*H*-CO), 7.56 (2H, d, *J* = 7.1 Hz, Ar-*H*), 7.41–7.39 (2H, m, Ar-*H*), 6.71–6.69 (3H, m, Ar-*H*), 5.54 (2H, br s, NH_2_). ^13^C-NMR (125 MHz, DMSO-*d_6_*): δ 188.2, 151.4, 143.0, 135.2, 131.1, 130.0, 128.2, 122.1, 121.2, 119.8, 113.9. 

*(E)-1-(4-Aminophenyl)-3-(4-hydroxyphenyl)prop-2-en-1-one* (**B**): 

Yellow solid; yield 89%; mp 79–80 °C. IR (ATR): 3339, 3326 (NH_2_), 3038, 1655 (C=O), 1593 (CH=CH). ^1^H-NMR (500 MHz, DMSO-*d_6_*): δ 7.97 (2H, d, *J* = 7.1 Hz, Ar-*H*), 7.61 (1H, d, *J* = 15.7 Hz, C*H*=CH-CO), 7.49 (2H, d, *J* = 7.1 Hz, Ar-*H*), 7.31 (1H, d, *J* = 15.7 Hz, CH=C*H*-CO), 7.06 (2H, d, *J* = 7.1 Hz, Ar-*H*), 6.89 (2H, d, *J* = 7.1 Hz, Ar-*H*), 5.48 (2H, br s, N*H*_2_). ^13^C-NMR (125 MHz, DMSO-*d_6_*): δ 189.1, 159.9, 152.8, 144.3, 139.9, 128.5, 126.6, 125.4, 118.6, 117.3, 115.7.

*(E)-1-(4-Aminophenyl)-3-(3-hydroxyphenyl)prop-2-en-1-one* (**C**): 

Burgundy solid; yield 83%; mp 142–143 °C. IR (ATR): 3335, 3326 (NH_2_), 3026, 1654 (C=O), 1595 (CH=CH). ^1^H-NMR (500 MHz, DMSO-*d_6_*): δ 7.99 (2H, d, *J* = 7.1 Hz, Ar-*H*), 7.63 (1H, d, *J* = 15.7 Hz, C*H*=CH-CO), 7.36 (2H, d, *J* = 7.1 Hz, Ar-*H*), 7.22 (1H, d, *J* = 15.7 Hz, CH=C*H*-CO), 7.08–7.02 (3H, m, Ar-*H*), 6.81–6.80 (1H, m, Ar-*H*), 5.47 (2H, br s, N*H*_2_). ^13^C-NMR (125 MHz, DMSO-*d_6_*): δ 189.7, 156.7, 152.7, 144.4, 136.1, 130.2, 127.5, 124.6, 119.2, 118.1, 117.9, 117.6, 114.3.

*(E)-1-(4-Aminophenyl)-3-(2-hydroxyphenyl)prop-2-en-1-one* (**D**): 

Light brown solid; yield 93%; mp 127–130 °C. IR (ATR): 3330, 3327 (NH_2_), 3029, 1654 (C=O), 1599 (CH=CH). ^1^H-NMR (500 MHz, DMSO-*d_6_*): δ 7.97 (2H, d, *J* = 7.1 Hz, Ar-*H*), 7.88 (1H, d, *J* = 15.7 Hz, C*H*=CH-CO), 7.64 (2H, d, *J* = 7.1 Hz, Ar-*H*), 7.56 (1H, d, *J* = 15.7 Hz, CH=C*H*-CO), 7.25–7.06 (4H, m, Ar-*H*), 5.49 (2H, br s, N*H*_2_). ^13^C-NMR (125 MHz, DMSO-*d_6_*): δ 188.8, 159.7, 152.8, 139.8, 132.1, 129.6, 128.5, 126.5, 121.0, 119.7, 118.6, 117.7, 114.3.

#### 4.2.2. General Method for Synthesis of Stilbenes

4-Aminostilbene precursors were synthesized by the Wittig–Horner reaction based on [36,37]. 

In a three-necked flask was mixed one eq (10 mmol) of benzyl chloride or X-(chloromethyl)phenol with 1.2 eq (12 mmol) of triethyl phosphite. The reaction mixture was stirred under the argon atmosphere and heated at 150 °C for 3 h. Then, the reaction mixture was cooled in an ice bath, and 25 mL of dry DMF was added. With continuous cooling and stirring, the 1.5 eq (15 mmol) of solid NaH was added portionwise. After the completion of the added NaH, the reaction mixture was cooled and stirred for another 30 min. Then, one eq (10 mmol) of 4-amino benzaldehyde dissolved in 10 mL of dry DMF was added dropwise. Then, the reaction mixture was stirred at room temperature for another 15 h. The reaction was monitored by TLC: eluent ethyl-acetate: hexane = 1:4; UV light (254 and 356 nm), I_2_ vapors, and anisidine reagent (at 180 °C) were used for the detection of spots. The pure product was obtained by pouring the reaction mixture at 100 g of crushed ice and recrystallization from methanol.

Stilbenes synthesized by the Wittig–Horner reaction have an exclusively *trans* configuration. [36,37] Derivatives **E**, **F**, **G,** and **H** were *trans* configuration confirmed by the presence of two doublets in area after 7 ppm with characteristical interaction constant *J* = 16.1 Hz [56] at ^1^H-NMR spectra.

*(E)-4-Styrylaniline* (**E**): 

Ocher solid; yield 71%; mp 124–126 °C. IR (ATR): 3174 (NH_2_), 3059, 1687 (CH=CH), 1588, 1559. ^1^H-NMR (500 MHz, DMSO-*d_6_*): δ 7.52 (2H, d, *J* = 7.1 Hz, Ar-*H*), 7.35 (2H, d, *J* = 7.1 Hz, Ar-*H*), 7.29 (2H, d, *J* = 8.5 Hz, Ar-*H*), 7.22 (1H, d, *J* = 7.8 Hz, Ar-*H*) 7.02 (1H, d, *J* = 16.1 Hz, CH=CH), 6.97 (1H, d, *J* = 16.1 Hz, CH=CH), 6.82 (2H, d, *J* = 8.5 Hz, Ar-*H*), 4.53 (2H, br s, N*H*_2_). ^13^C-NMR (125 MHz, DMSO-*d_6_*): δ 137.4, 128.8, 128.6, 127.7, 126.4, 125.8, 125.6, 123.7, 114.2.

*(E)-4-(4-Aminostyryl)phenol* (**F**): 

Light brown solid; yield 79%; mp 81–84 °C. IR (ATR): 3174, 3168 (OH, NH_2_), 3059, 1681 (CH=CH), 1591, 1228, 1025. ^1^H-NMR (500 MHz, DMSO-*d_6_*): δ 9.18 (1H, br s, O*H*), 7.43 (2H, d, *J* = 8.5 Hz, Ar-*H*), 7.13 (2H, d, *J* = 8.1 Hz, Ar-*H*), 6.91 (1H, d, *J* = 16.1 Hz, CH=CH), 6.87 (2H, d, *J* = 8.5 Hz, Ar-*H*), 6.81 (1H, d, *J* = 16.1 Hz, CH=CH), 6.52 (2H, d, *J* = 8.5 Hz, Ar-*H*), 5.16 (2H, br s, N*H*_2_). ^13^C-NMR (125 MHz, DMSO-*d_6_*): δ 156.8, 148.5, 129.4, 127.5, 127.4, 126.3, 125.7, 123.4, 117.6, 115.4.

*(E)-3-(4-Aminostyryl)phenol* (**G**): 

Yellow solid; yield 78%; mp 129–131°C. IR (ATR): 3168, 3163 (OH, NH_2_), 3074, 1681 (CH=CH), 1590, 1258, 1025. ^1^H-NMR (500 MHz, DMSO-*d_6_*): δ 9.08 (1H, br s, O*H*), 7.29 (2H, d, *J* = 8.5 Hz, Ar-*H*), 7.12 (2H, d, *J* = 7.8 Hz, Ar-*H*), 6.89 (1H, d, *J* = 16.1 Hz, CH=CH), 6.87–6.84 (3H, m, Ar-*H*), 6.79 (1H, d, *J* = 16.1 Hz, CH=CH), 6.57–6.55 (1H, Ar-*H*), 5.24 (2H, br s, N*H*_2_). ^13^C-NMR (125 MHz, DMSO-*d_6_*): δ 157.6, 148.8, 139.4, 124.6, 129.4, 128.7, 126.6, 123.1, 119.8, 118.7, 116.9, 112.4.

*(E)-2-(4-Aminostyryl)phenol* (**H**): 

Yellow solid; yield 87%; mp 137–139 °C. IR (ATR): 3361, 3206 (OH, NH_2_), 3074, 2991, 1719 (CH=CH), 1590, 1277, 1025. ^1^H-NMR (500 MHz, DMSO-*d_6_*): δ 9.11 (1H, br s, O*H*), 7.48 (2H, d, *J* = 7.8 Hz, Ar-*H*), 7.29 (2H, d, *J* = 7.8 Hz, Ar-*H*), 7.18 (1H, d, *J* = 16.1 Hz, CH=CH), 7.05 (2H, d, *J* = 8.5 Hz, Ar-*H*), 6.82 (1H, d, *J* = 16.1 Hz, CH=CH), 6.63 (2H, d, *J* = 8.5 Hz, Ar-*H*), 5.01 (2H, br s, N*H*_2_). ^13^C-NMR (125 MHz, DMSO-*d_6_*): δ 155.5, 148.2, 129.8, 129.7, 128.7, 128.4, 128.3, 127.1, 126.6, 121.0, 120.7, 116.6.

*Synthesis of X-hydroxybenzylchlorides:*

Commercially unavailable X-hydroxybenzylchlorides (**I**, **J**, **K**, **L**) were prepared by modified synthesis [57]. In our modification, the synthesis was carried out without the solvent, and the DMF was used as the organic catalyst. This modification achieved significantly higher %yields. In the flask equipped with the calcium chloride drying tube was cooled the mixture of 1 eq (20 mmol) of benzylalcohol or X-(hydroxymethyl)phenol and a catalytical amount of DMF in an ice bath (0–5 °C). Then, the X-(hydroxymethyl)phenol was overlayed with 3 eq (60 mmol) of SOCl_2_. The reaction mixture was vigorously stirred for 10 min under intensive cooling. Then, the reaction mixture was stirred for 1 h at room temperature. The reaction was monitored by TLC: eluent EtOH, UV light (254 and 356 nm), and anisidine reagent (at 180 °C) were used for the detection of spots. After the completion of the reaction, the reaction mixture was dissolved in 10 mL of chloroform and was washed five times with 20 mL of distilled water. The organic layer was dried with anhydrous sodium sulfate. Chloroform was evaporated at a rotary vacuum evaporator (15 mbar, 25 °C). 

*(Chloromethyl)benzene* (**I**): 

Light yellow oil; yield 51%. IR (ATR): 3354 (OH), 1587, 1483 (C=C), 1284, 1241, 1154, 782, 756. ^1^H-NMR (500 MHz, DMSO-*d_6_*): δ 7.41–7.39 (3H, m, Ar-*H*), 7.36–7.31 (2H, m, Ar-*H*), 4.63 (2H, s, C*H*_2_). ^13^C-NMR (125 MHz, DMSO-*d_6_*): δ 137.1, 128.6, 128.1, 127.9, 46.1.

*4-(Chloromethyl)phenol* (**J**): 

Light yellow oil; yield 62%. IR (ATR): 3354 (OH), 1596, 1457 (C=C), 1281, 1256, 1153, 787, 751. ^1^H-NMR (500 MHz, DMSO-*d_6_*): δ 7.47 (2H, d, *J* = 7.45 Hz, Ar-*H*), 7.18 (2H, d, *J* = 7.15 Hz, Ar-*H*), 4.89 (1H, br s, O*H*), 4.61 (2H, s, C*H*_2_). ^13^C-NMR (125 MHz, DMSO-*d_6_*): δ 158.8, 136.0, 129.8, 121.3, 45.3. 

*3-(Chloromethyl)phenol* (**K**): 

Light yellow solid; yield 78%. IR (ATR): 3356 (OH), 1596, 1455 (C=C), 1291, 1254, 1152, 787, 753. ^1^H-NMR (500 MHz, DMSO-*d_6_*): δ 7.48 (1H, t, *J* = 7.9 Hz, Ar-*H*), 6.95–6.94 (2H, m, Ar-*H*), 6.80–6.79 (1H, m, Ar-*H*), 4.96 (1H, br s, O*H*), 4.36 (2H, s, C*H*_2_). ^13^C-NMR (125 MHz, DMSO-*d_6_*): δ 156.3, 139.4, 132.1, 120.9, 115.8, 46.2.

*2-(Chloromethyl)phenol* (**L**): 

Light yellow oil; yield 65%. IR (ATR): 3359 (OH), 1584, 1461 (C=C), 1287, 1254, 1159, 776, 748. ^1^H-NMR (500 MHz, DMSO-*d_6_*): δ 7.51–7.48 (1H, m, Ar-*H*), 7.12–7.06 (3H, m, Ar-*H*), 5.01 (1H, br s, O*H*), 4.42 (2H, s, C*H*_2_). ^13^C-NMR (125 MHz, DMSO-*d_6_*): δ 155.8, 140.1, 131.7, 122.3, 117.6, 115.8, 45.9.

*Preparation of 4-aminobenzaldehyde:*

4-Aminobenzaldehyde was synthesized according to the [58]. Obtained ^1^H-NMR spectra and ^13^C-NMR spectra are in agreement with those previously reported [58].

#### 4.2.3. General Method for Synthesis of Starting 4,6-Dichloro-1,3,5-triazin-2-yl Aminobenzenesulfonamides

Starting 4-[(4,6-dichloro-1,3,5-triazin-2-yl)amino]benzene-1-sulfonamide, 4-{[(4,6-dichloro-1,3,5-triazin-2-yl)amino]methyl}benzene-1-sulfonamide, and 4-{2-[(4,6-dichloro-1,3,5-triazin-2-yl)amino]ethyl}benzene-1-sulfonamide were prepared according to the literature [46]. All spectral data are in the agreement with those of previously reported [46].

#### 4.2.4. General Method for Synthesis of Disubstituted Derivatives of 1,3,5-Triazine Containing Aminobenzene Sulfonamide or Aminoalcohol/Phenol Structural Motifs (**1**, **2**, **5**–**8**, **18**–**20**)

Compounds (**1**, **2**, **5**–**8**, and **18**–**20**) were prepared according to the methodology published in [38,39]. All spectral data of known compounds (**1**, **2**, **5**–**8**, and **18**–**20**) are in agreement with those previously reported [39].

#### 4.2.5. General Method for Synthesis of Trisubstituted Derivatives of 1,3,5-Triazine Containing Aminobenzene Sulfonamide or Aminoalcohol/Phenol Structural Motifs (3, 9–12)

Compounds (**3**, **9**–**12**) were prepared according to the methodology published in [38,39]. All spectral data of known compounds (**3**, **9**–**12**) are in agreement with those previously reported [39].

#### 4.2.6. General Method for Synthesis of Trisubstituted Derivatives of 1,3,5-Triazine Containing Chalcone Structural Motif (**4**, **13**–**16**, **21**–**29**) 

Compounds were prepared according to the methodology published in [38,40]. 

Step 1: Starting dichlorotriazinyl benzenesulfonamide (1 mmol) was dissolved in 10 mL of DMF. One mmol of solid anhydrous potassium carbonate was added gradually, and the mixture was stirred for 10 min. Then, one mmol of the appropriate nucleophile was added portionwise. Finally, 2.5% mol of supported Cu(I) ions were added into the reaction mixture. The reaction was stirred at 35 °C until the maximum conversion of starting reactants was achieved (monitored by TLC). After the completion of a reaction, the catalyst and salt were filtered off. Crushed ice was then added into the solution, and the formed precipitate was collected by filtration. The crude product was dissolved in hot acetone and precipitated by the addition of isopropyl alcohol.

Step 2: Appropriate chalcone (1 mmol) was dissolved in 15 mL of DMF. One mmol of solid anhydrous potassium carbonate was added in small portions, and the mixture was stirred for 15 min. Then, one mmol of the appropriate nucleophile (a disubstituted derivative of cyanuric chloride) was added portionwise. Finally, 2.5% mol of supported Cu(I) ions were added into the reaction mixture. The reaction was stirred at 110 °C until the maximum conversion of starting reactants was achieved (monitored by TLC). After the completion of a reaction, the catalyst and salt were filtered off. The filtrate was concentrated to 1/5 of the original volume by a rotary vacuum evaporator. The pure product was obtained by the addition of the mixture of isopropyl alcohol: diethyl ether (1:10) and filtered off. 

All spectral data of known compounds (**13**–**15**, **22**–**24**) are in agreement with those previously reported [40].

#### 4.2.7. General Method for Synthesis of Trisubstituted Derivatives of 1,3,5-Triazine Containing Stilbene Structural Motif (**17**, **30**–**44**)

Compounds were prepared according to the methodology published in [38,40]. 

Step 1: Starting dichlorotriazinyl benzenesulfonamide (1 mmol) was dissolved in 10 mL of DMF. One mmol of solid anhydrous potassium carbonate was added in small portions, and the mixture was stirred for 10 min. Next, 1 mmol of the appropriate nucleophile was added portionwise. Finally, 2.5% mol of supported Cu(I) ions were added into the reaction mixture. The reaction mass was stirred at 35 °C until the maximum conversion of starting reactants was achieved (monitored by TLC). After the completion of a reaction, the catalyst and salt were filtered off. Crushed ice was added into the solution, and the formed precipitate was collected by filtration. The crude product was dissolved in hot acetone and precipitated by the addition of isopropyl alcohol.

Step 2: Appropriate stilbene (1 mmol) was dissolved in 15 mL of DMF. One mmol of solid anhydrous potassium carbonate was added in small portions, and the mixture was stirred for 15 min. Then, one mmol of the appropriate nucleophile (a disubstituted derivative of cyanuric chloride) was added portionwise. Finally, 2.5% mol of supported Cu(I) ions were added to the reaction mixture. The reaction mass was stirred at 130 °C until the maximum conversion of the starting reactants was achieved (monitored by TLC). After the completion of the reaction, the catalyst and salt were filtered off. The filtrate was concentrated to 1/10 of the original volume by a rotary vacuum evaporator, and 15 mL of isopropyl alcohol was added. The mixture was cooled at 0–5 °C overnight. The obtained pure product was filtered off. The other portion of pure product was obtained as follows: The filtrate was treated with a few drops of diethyl ether, and then the solvent was evaporated entirely by a rotary vacuum evaporator. Then, 2 mL of cold water was poured into the mixture, and the crystals were formed. The mixture was cooled to 0–5 °C (in the fridge) for 72 h and was then filtered.

All spectral data of known compounds (**17**, **35**, **37**, **39**, **43**) are in agreement with those previously reported [40].

#### 4.2.8. Characterization of Novel Compounds **4**, **16**, **21**, **25**–**34**, **36**, **38**, **40**–**42**, **44**

*(E)-4-({4-[(3-Hydroxypropyl)amino]-6-{[4-(3-hydroxystyryl)phenyl]amino}-1,3,5-triazin-2-yl}amino)benzenesulfonamide* (**4**): 

Brown solid; yield 40%; mp 133–134 °C. IR (ATR): 3386, 3221 (OH, NH, NH_2_), 3075, 3055, 2928, 2911, 1679 (CH=CH), 1590, 1573 (C=C, C=N), 1332 (SO_2_NH_2_), 1232, 1156 (SO_2_NH_2_), 1026. ^1^H-NMR (500 MHz, DMSO-*d_6_*): δ 9.13 (5H, br s, N*H*, N*H*_2_), 7.82 (2H, d, *J* = 7.8 Hz, Ar-*H*), 7.79 (2H, d, *J* = 7.8 Hz, Ar-*H*), 7.71–7.69 (1H, m, Ar-*H*), 7.68–7.66 (3H, m, Ar-*H*), 7.58 (2H, d, *J* = 8.5 Hz, Ar-*H*), 7.55 (2H, d, *J* = 8.5 Hz, Ar-*H*), 7.12–7.09 (2H, m, CH=CH), 6.91 (2H, br s, O*H*), 3.51–3.49 (2H, m, C*H*_2_-OH), 3.39–3.35 (2H, m, NH-C*H*_2_), 1.98–1.96 (2H, m, NH-CH_2_C*H*_2_). ^13^C-NMR (125 MHz, DMSO-*d_6_*): δ 166.4, 166.1, 159.8, 158.3, 144.1, 143.2, 142.7, 141.6, 139.8, 131.1, 129.7, 129.6, 129.4, 129.1, 128.7, 126.6, 126.4, 126.1, 125.9, 56.7, 37.5, 32.4. 

*(E)-4-[({4-({4-[3-(4-Hydroxyphenyl)acryloyl]phenyl}amino)-6-[(3-hydroxypropyl)amino]-1,3,5-triazin-2-yl}amino)methyl]benzenesulfonamide* (**16**): 

Ocher solid; yield 76%; mp 120–121 °C. IR (ATR): 3334, 3220 (OH, NH, NH_2_), 2936, 1689, 1631, 1591(C=C, C=N, C=O), 1574, 1345, 1158 (SO_2_NH_2_), 1086, 1026. ^1^H-NMR (500 MHz, DMSO-*d_6_*): δ 7.97 (2H, d, *J* = 8.1 Hz, Ar-*H*), 7.84 (2H, d, *J* = 8.1 Hz, Ar-*H*), 7.79 (1H, d, *J* = 15.5 Hz, C*H*=CH-CO), 7.61 (2H, d, *J* = 8.1 Hz, Ar-*H*), 7.59 (1H, d, *J* = 15.5 Hz, CH=C*H*-CO), 7.31 (2H, d, *J* = 8.1 Hz, Ar-*H*), 6.90 (2H, d, *J* = 8.1 Hz, Ar-*H*), 6.76 (7H, s, O*H*, N*H*, N*H*_2_), 4.62–4.61 (2H, m, C*H*_2_), 3.62–3.60 (2H, m, C*H*_2_-OH), 3.44–3.41 (2H, m, NH-C*H*_2_),1.92–1.89 (2H, m, NH-CH_2_C*H*_2_). ^13^C-NMR (125 MHz, DMSO-*d_6_*): δ 189.5, 168.7, 165.9, 165.1, 146.8, 144.3, 144.0, 142.1, 142.0, 141.9, 138.6, 130.9, 128.4, 126.9, 126.3, 120.1, 119.1, 114.5, 57.9, 44.4, 38.6, 32.5. 

*(E)-4-[2-({4-[(4-Cinnamoylphenyl)amino]-6-[(4-hydroxyphenyl)amino]-1,3,5-triazin-2-yl}amino)ethyl]benzenesulfonamide* (**21**): 

Brown solid; yield 54%; mp 263–264 °C. IR (ATR): 3329, 3224 (OH, NH, NH_2_), 2937, 1687, 1675, 1623, 1583 (C=C, C=N, C=O), 1557 1338, 1159 (SO_2_NH_2_), 1036. ^1^H-NMR (500 MHz, DMSO-*d_6_*): δ 7.95 (2H, d, *J* = 8.1 Hz, Ar-*H*), 7.79 (2H, d, *J* = 8.1 Hz, Ar-*H*), 7.71 (1H, d, *J* = 15.5 Hz, C*H*=CH-CO), 7.56 (2H, d, *J* = 8.1 Hz, Ar-*H*), 7.49 (1H, d, *J* = 15.5 Hz, CH=C*H*-CO), 7.37 (2H, d, *J* = 8.1 Hz, Ar-*H*), 7.32 (2H, d, *J* = 8.1 Hz, Ar-*H*), 7.29–7.26 (3H, m, Ar-*H*), 6.91 (6H, s, O*H*, N*H*, N*H*_2_), 6.85 (2H, d, *J* = 8.1 Hz, Ar-*H*), 6.52 (2H, d, *J* = 8.1 Hz, Ar-*H*), 3.71–3.69 (2H, m, C*H*_2_), 2.96–2.93 (2H, m, C*H*_2_). ^13^C-NMR (125 MHz, DMSO-*d_6_*): δ 189.1, 168.1, 166.1, 165.7, 156.5, 144.9, 143.2, 141.3, 136.1, 135.7, 132.8, 130.4, 129.3, 128.4, 125.7, 124.7, 123.1, 121.4, 120.0, 118.1, 116.1, 43.4, 36.7.

*(E)-4-[2-({4-({4-[3-(3-Hydroxyphenyl)acryloyl]phenyl}amino)-6-[(3-hydroxypropyl)amino]-1,3,5-triazin-2-yl}amino)ethyl]benzenesulfonamide* (**25**): 

Yellow solid; yield 81%; mp 381–382 °C. IR (ATR): 3329, 3221 (OH, NH, NH_2_), 2941, 1698, 1681, 1668, 1662, 1653, 1634 (C=C, C=N, C=O), 1580, 1346, 1182 (SO_2_NH_2_), 1079, 1034. ^1^H-NMR (500 MHz, DMSO-*d_6_*): δ 7.92 (2H, d, *J* = 8.1 Hz, Ar-*H*), 7.88 (2H, d, *J* = 8.1 Hz, Ar-*H*), 7.76 (1H, d, *J* = 15.5 Hz, C*H*=CH-CO), 7.54 (2H, d, *J* = 8.1 Hz, Ar-*H*), 7.48 (1H, d, *J* = 15.5 Hz, CH=C*H*-CO), 7.29 (2H, d, *J* = 8.1 Hz, Ar-*H*), 7.18 (2H, d, *J* = 8.1 Hz, Ar-*H*), 6.99 (7H, s, O*H*, N*H*, N*H*_2_), 4.02–3.99 (2H, m, C*H*_2_), 3.58–3.55 (2H, m, C*H*_2_-OH), 3.38–3.34 (2H, m, NH-C*H*_2_), 2.86–2.85 (2H, m, C*H*_2_), 1.97–1.95 (2H, m, NH-CH_2_C*H*_2_). ^13^C-NMR (125 MHz, DMSO-*d_6_*): δ 189.2, 168.4, 166.6, 166.1, 158.9, 144.5, 143.7, 141.5, 139.7, 140.4, 139.0, 131.1, 129.7, 128.2, 125.8, 119.4, 118.7, 115.9, 57.1, 43.8, 38.6, 33.9, 32.1.

*(E)-4-[2-({4-({4-[3-(3-Hydroxyphenyl)acryloyl]phenyl}amino)-6-[(4-hydroxyphenyl)amino]-1,3,5-triazin-2-yl}amino)ethyl]benzenesulfonamide* (**26**): 

Brown solid; yield 58%; mp 176–178 °C. IR (ATR): 3328, 3219 (OH, NH, NH_2_), 2937, 1698, 1682, 1667, 1661, 1653, 1614 (C=C, C=N, C=O), 1576, 1350, 1197 (SO_2_NH_2_), 1079, 1034. ^1^H-NMR (500 MHz, DMSO-*d_6_*): δ 7.92 (2H, d, *J* = 8.1 Hz, Ar-*H*), 7.87 (2H, d, *J* = 8.1 Hz, Ar-*H*), 7.69 (1H, d, *J* = 15.5 Hz, C*H*=CH-CO), 7.59 (2H, d, *J* = 8.1 Hz, Ar-*H*), 7.51 (1H, d, *J* = 15.5 Hz, CH=C*H*-CO), 7.36 (2H, d, *J* = 8.1 Hz, Ar-*H*), 7.13 (2H, d, *J* = 8.1 Hz, Ar-*H*), 6.97 (7H, s, O*H*, N*H*, N*H*_2_), 6.86 (2H, d, *J* = 8.1 Hz, Ar-*H*), 6.81 (2H, d, *J* = 8.1 Hz, Ar-*H*), 3.71–3.69 (2H, m, C*H*_2_), 2.30–2.28 (2H, m, C*H*_2_). ^13^C-NMR (125 MHz, DMSO-*d_6_*): δ 188.7, 167.1, 166.8, 166.4, 159.6, 155.3, 144.1, 141.7, 139.6, 135.6, 135.1, 132.6, 132.3, 129.4, 128.3, 125.5, 124.9, 123.8, 121.3, 119.9, 118.0, 115.7, 43.2, 36.7.

*(E)-4-[2-({4-({4-[3-(4-Hydroxyphenyl)akryloyl]phenyl}amino)-6-[(3-hydroxypropyl)amino]-1,3,5-triazin-2-yl}amino)ethyl]benzenesulfonamide* (**27**): 

Ocher solid; yield 77%; mp 138–139 °C. IR (ATR): 3336, 3221 (OH, NH, NH_2_), 2937, 1698, 1683, 1630, 1592 (C=C, C=N, C=O), 1576, 1345, 1164, (SO_2_NH_2_), 1085, 1025. ^1^H-NMR (500 MHz, DMSO-*d_6_*): δ 7.95 (2H, d, *J* = 8.1 Hz, Ar-*H*), 7.86 (2H, d, *J* = 8.1 Hz, Ar-*H*), 7.78 (1H, d, *J* = 15.5 Hz, C*H*=CH-CO), 7.57 (2H, d, *J* = 8.1 Hz, Ar-*H*), 7.49 (1H, d, *J* = 15.5 Hz, CH=C*H*-CO), 7.32 (2H, d, *J* = 8.1 Hz, Ar-*H*), 7.21 (2H, d, *J* = 8.1 Hz, Ar-*H*), 6.95 (7H, s, O*H*, N*H*, N*H*_2_), 4.13–4.09 (2H, m, C*H*_2_), 3.62–3.59 (2H, m, C*H*_2_-OH), 3.42–3.39 (2H, m, NH-C*H*_2_), 2.90–2.87 (2H, m, C*H*_2_), 2.04–1.98 (2H, m, NH-CH_2_C*H*_2_). ^13^C-NMR (125 MHz, DMSO-*d_6_*): δ 189.3, 168.5, 166.5, 165.9, 158.7, 144.2, 143.8, 141.3, 139.6, 140.2, 138.9, 130.5, 129.4, 128.0, 125.7, 119.8, 118.6, 115.8, 57.3, 43.6, 38.4, 33.8, 31.7.

*(E)-4-[({4-({4-[3-(4-Hydroxyphenyl)acryloyl]fenyl}amino)-6-[(2,3-dihydroxypropyl)amino]-1,3,5-triazin-2-yl}amino)methyl]benzenesulfonamide* (**28**): 

Yellow solid; yield 98%; mp 121–122 °C. IR (ATR): 3352, 3220 (OH, NH, NH_2_), 2932, 1698, 1683 1588 (C=C, C=N, C=O), 1578 1333, 1155 (SO_2_NH_2_), 1091, 1026. ^1^H-NMR (500 MHz, DMSO-*d_6_*): δ 7.91 (2H, d, *J* = 8.1 Hz, Ar-*H*), 7.87 (2H, d, *J* = 8.1 Hz, Ar-*H*), 7.82 (1H, d, *J* = 15.5 Hz, C*H*=CH-CO), 7.76 (2H, d, *J* = 8.1 Hz, Ar-*H*), 7.45 (2H, d, *J* = 8.1 Hz, Ar-*H*), 7.36 (1H, d, *J* = 15.5 Hz, CH=C*H*-CO), 6.90 (2H, d, *J* = 8.1 Hz, Ar-*H*), 6.68 (2H, d, *J* = 8.1 Hz, Ar-*H*), 6.12 (8H, s, O*H*, N*H*, N*H*_2_), 4.53–4.51 (1H, m, C*H*-OH), 3.79–3.74 (4H, m, NH-C*H*_2_, C*H*_2_-OH), 3.19–3.16 (2H, m, C*H*_2_), 2.88–2.86 (2H, m, C*H*_2_). ^13^C-NMR (125 MHz, DMSO-*d_6_*): δ 186.3, 170.7, 167.7, 167.2, 154.3, 144.2, 144.1, 142.5, 142.0, 141.0, 139.8, 131.2, 129.6, 129.5, 126.2, 126.1, 125.9, 113.2, 70.9, 62.5, 44.5, 42.3, 36.1.

*(E)-4-[2-({4-({4-[3-(4-Hydroxyphenyl)acryloyl]phenyl}amino)-6-[(4-hydroxyphenyl)amino]-1,3,5-triazin-2-yl}amino)ethyl]benzenesulfonamide* (**29**): 

Orange solid; yield 87%; mp 254–255 °C. IR (ATR): 3331, 3223 (OH, NH, NH_2_), 2947, 1698, 1682, 1651, 1646 (C=C, C=N, C=O), 1582, 1339, 1163 (SO_2_NH_2_), 1082, 1032. ^1^H-NMR (500 MHz, DMSO-*d_6_*): δ 7.95 (2H, d, *J* = 8.1 Hz, Ar-*H*), 7.85 (2H, d, *J* = 8.1 Hz, Ar-*H*), 7.71 (1H, d, *J* = 15.5 Hz, C*H*=CH-CO), 7,62 (2H, d, *J* = 8.1 Hz, Ar-*H*), 7.53 (1H, d, *J* = 15.5 Hz, CH=C*H*-CO), 7.34 (2H, d, *J* = 8.1 Hz, Ar-*H*), 7.15 (2H, d, *J* = 8.1 Hz, Ar-*H*), 6.96 (7H, s, O*H*, N*H*, N*H*_2_), 6.85 (2H, d, *J* = 8.1 Hz, Ar-*H*), 6.78 (2H, d, *J* = 8.1 Hz, Ar-*H*), 3.70–3.67 (2H, m, C*H*_2_), 2.68–2.66 (2H, m, C*H*_2_). ^13^C-NMR (125 MHz, DMSO-*d_6_*): δ 188.9, 167.3, 166.5, 166.1, 159.4, 155.1, 144.4, 139.9, 139.1, 135.3, 134.9, 132.5, 131.8, 129.5, 128.1, 125.2, 124.4, 123.2, 121.6, 119.7, 118.1, 115.4, 42.3, 36.0. 

*(E)-4-[2-({4-[(3-Hydroxypropyl)amino]-6-[(4-styrylphenyl)amino]-1,3,5-triazin-2-yl}amino)ethyl]benzenesulfonamide* (**30**): 

Yellow solid; yield 79%; mp 132–133 °C. IR (ATR): 3401, 3267 (OH, NH, NH_2_), 3112, 3096, 2994, 2956, 1670 (CH=CH), 1636, 1557 (C=C, C=N), 1329 (SO_2_NH_2_), 1242, 1151 (SO_2_NH_2_), 1025. ^1^H-NMR (500 MHz, DMSO-*d_6_*): δ 9.18 (5H, br s, N*H*, N*H*_2_), 7.75 (2H, d, *J* = 7.8 Hz, Ar-*H*), 7.69–7.66 (3H, m, Ar-*H*), 7.58 (2H, d, *J* = 8.0 Hz, Ar-*H*), 7.49 (2H, d, *J* = 8.0 Hz, Ar-*H*), 7.36 (2H, d, *J* = 8.0 Hz, Ar-*H*), 7.01 (2H, d, *J* = 7.8 Hz, Ar-*H*), 6.89 (1H, d, *J* = 16.1 Hz, CH=CH), 6.74 (1H, d, *J* = 16.1 Hz, CH=CH), 6.01 (1H, br s, O*H*), 3.61 (2H, t, *J* = 7.8 Hz, C*H*_2_), 3.48–3.44 (2H, m, C*H*_2_-OH), 3.36–3.34 (2H, m, NH-C*H*_2_), 2.91–2.89 (2H, m, C*H*_2_), 2.03–1.99 (2H, m, NH-CH_2_C*H*_2_). ^13^C-NMR (125 MHz, DMSO-*d_6_*): δ 168.6, 166.6, 166.1, 143.9, 142.2, 139.7, 136.4, 130.9, 129.7, 129.3, 128.6, 127.4, 126.7, 126.1, 125.7, 124.7, 121.1, 57.9, 43.7, 38.4, 34.8, 31.9. 

*(E)-4-{2-[(4-[(2,3-Dihydroxypropyl)amino]-6-[(4-styrylphenyl)amino]-1,3,5-triazin-2-yl)amino]ethyl}benzenesulfonamide* (**31**): 

Ocher solid; yield 44%; mp 131–134 °C. IR (ATR): 3412, 3253 (OH, NH, NH_2_), 3077, 3055, 2991, 2947, 1681 (CH=CH), 1591, 1575 (C=C, C=N), 1333 (SO_2_NH_2_), 1158 (SO_2_NH_2_), 1028. ^1^H-NMR (500 MHz, DMSO-*d_6_*): δ 9.03 (3H, br s, N*H*) 7.77 (2H, d, *J* = 7.8 Hz, Ar-*H*), 7.73 (2H, d, *J* = 7.8 Hz, Ar-*H*), 7.67 (2H, d, *J* = 8.0 Hz, Ar-*H*), 7.59 (2H, d, *J* = 8.0 Hz, Ar-*H*), 7.41–7.39 (2H, d, *J* = 8.0 Hz, Ar-*H*), 7.28–7.23 (3H, m, Ar-*H*), 7.06–7.03 (2H, m, CH=CH), 6.71 (2H, br s, N*H*_2_), 6.64 (2H, br s, O*H*), 4.38–4.37 (2H, m, NH-C*H*_2_), 3.57–3.53 (3H, m, C*H*-OH, C*H*_2_-OH). ^13^C-NMR (125 MHz, DMSO-*d_6_*): δ 165.4, 165.3, 165.2, 144.4, 144.3, 144.1, 143.2, 142.8, 133.2, 132.6, 132.0, 131.4, 124.6, 124.1, 122.3, 121.1, 117.8, 68.3, 64.1, 45.2.

*(E)-4-{2-[(4-[(4-Hydroxyphenyl)amino]-6-[(4-styrylphenyl)amino]-1,3,5-triazin-2-yl)amino]ethyl}benzenesulfonamide* (**32**): 

Brown solid; yield 62%; mp 82–84 °C. IR (ATR): 3224 (NH, NH_2_), 3060, 3054, 2989, 2913, 1705 (CH=CH), 1591, 1574 (C=C, C=N), 1332 (SO_2_NH_2_), 1228, 1158 (SO_2_NH_2_). ^1^H-NMR (500 MHz, DMSO-*d_6_*): δ 9.20 (5H, br s, N*H*, N*H*_2_), 7.75 (2H, d, *J* = 7.8 Hz, Ar-*H*), 7.71 (2H, d, *J* = 7.8 Hz, Ar-*H*), 7.41 (2H, d, *J* = 8.0 Hz, Ar-*H*), 7.32 (2H, d, *J* = 8.0 Hz, Ar-*H*), 7.26 (2H, d, *J* = 8.0 Hz, Ar-*H*), 7.17 (2H, d, *J* = 8.0 Hz, Ar-*H*), 7.09 (2H, d, *J* = 8.0 Hz, Ar-*H*), 6.78 (3H, m, Ar-*H*), 6.72 (1H, br s, O*H*), 6.65–6.62 (2H, m, CH=CH), 3.76 (2H, t, *J* = 7.8 Hz, C*H*_2_), 2.92 (2H, t, *J* = 7.8 Hz, C*H*_2_). ^13^C-NMR (125 MHz, DMSO-*d_6_*): δ 166.2, 165.9, 165.7, 156.8, 144.1, 143.7, 142.9, 142.3, 135.6, 132.4, 130.0, 129.7, 129.2, 128.7, 128.4, 128.3, 127.1, 123.2, 122.5, 119.1, 116.3, 43.6, 35.1. 

*(E)-4-{2-[(3-[(4-Styrylphenyl)amino]-6-[(4-sulfamoylbenzyl)amino]phenyl)amino]ethyl} benzenesulfonamide **(*****33**): 

Ocher solid; yield 44%; mp 215–217 °C. IR (ATR): 3323, 3302 (NH, NH_2_), 3284, 3273, 2924, 2867, 1715 (CH=CH), 1661, 1567 (C=C, C=N), 1339 (SO_2_NH_2_), 1156 (SO_2_NH_2_). ^1^H-NMR (500 MHz, DMSO-*d_6_*): δ 9.01 (3H, s, N*H*), 7.81 (2H, d, *J* = 7.8 Hz, Ar-*H*), 7.68 (2H, d, *J* = 7.8 Hz, Ar-*H*), 7.61–7.58 (3H, m, Ar-*H*), 7.51 (2H, d, *J* = 8.0 Hz, Ar-*H*), 7.46 (2H, d, *J* = 8.0 Hz, Ar-*H*), 7.37 (2H, d, *J* = 8.0 Hz, Ar-*H*), 7.01 (2H, d, *J* = 7.8 Hz, Ar-*H*), 6.99 (2H, d, *J* = 7.8 Hz, Ar-*H*), 6.94 (4H, br s, N*H*_2_), 6.65–6.63 (2H, m, CH=CH), 4.60–4.58 (2H, m, NH-C*H*_2_), 3.62 (2H, t, *J* = 7.8 Hz, C*H*_2_), 2.95 (2H, t, *J* = 7.8 Hz, C*H*_2_). ^13^C-NMR (125 MHz, DMSO-*d_6_*): δ 166.5, 165.9, 165.6, 145.7, 143.9, 142.0, 139.9, 139.5, 136.4, 130.1, 129.5, 129.2, 129.1, 129.0, 128.7, 127.5, 126.7, 125.7, 125.1, 124.9, 121.0, 44.4, 43.6, 34.7.

*(E)-4-{2-[(4-[(2,3-Dihydroxypropyl)amino]-6-{[4-(2-hydroxystyryl)phenyl]amino}-1,3,5-triazin-2-yl)amino]ethyl}benzenesulfonamide* (**34**): 

Vanilla solid; yield 21%; mp 153–156 °C. IR (ATR): 3348, 3312 (OH, NH, NH_2_), 3075, 3051, 3037, 3021, 2991, 1621 (CH=CH), 1590, 1574, 1558 (C=C, C=N), 1347 (SO_2_NH_2_), 1264, 1188 (SO_2_NH_2_), 1027. ^1^H-NMR (500 MHz, DMSO-*d_6_*): δ 9.07 (3H, br s, N*H*), 7.82 (2H, d, *J* = 7.8 Hz, Ar-*H*), 7.78 (2H, d, *J* = 7.8 Hz, Ar-*H*), 7.73 (2H, d, *J* = 8.0 Hz, Ar-*H*), 7.71 (2H, d, *J* = 8.0 Hz, Ar-*H*), 7.59–7.52 (1H, m, Ar-*H*), 7.24 (2H, m, CH=CH), 6.83 (2H, br s, N*H*_2_), 6.61 (3H, br s, O*H*), 4.41–4.39 (2H, m, NH-C*H*_2_), 3.72 (2H, t, *J* = 7.8 Hz, C*H*_2_), 3.58–3.55 (3H, m, C*H*-OH, C*H*_2_-OH), 3.18–3.16 (2H, m, C*H*_2_). ^13^C-NMR (125 MHz, DMSO-*d_6_*): δ 166.8, 166.7, 166.4, 159.1, 144.2, 144.0, 143.4, 142.3, 141.9, 132.3, 131.8, 131.2, 131.6, 124.1, 123.6, 122.9, 121.3, 119.8, 119.7, 69.1, 62.8, 43.7, 43.4, 40.3. 

*(E)-4-{2-[(4-{[4-(2-Hydroxystyryl)phenyl]amino}-6-[(4-sulfamoylbenzyl)amino]-1,3,5-triazin-2-yl)amino]ethyl}benzenesulfonamide* (**36**): 

Vanilla solid; yield 63%; mp 119–121 °C. IR (ATR): 3314, 3281 (OH, NH, NH_2_), 3055, 3036, 2991, 1667 (CH=CH), 1589, 1571 (C=C, C=N), 1334 (SO_2_NH_2_), 1264, 1181 (SO_2_NH_2_). ^1^H-NMR (500 MHz, DMSO-*d_6_*): δ 9.22 (7H, br s, N*H*, N*H*_2_), 7.84 (2H, d, *J* = 7.8 Hz, Ar-*H*), 7.76 (2H, d, *J* = 7.8 Hz, Ar-*H*), 7.61–7.58 (4H, m, Ar-*H*), 7.52 (2H, d, *J* = 8.0 Hz, Ar-*H*), 7.41 (2H, d, *J* = 8.0 Hz, Ar-*H*), 7.00 (2H, d, *J* = 7.8 Hz, Ar-*H*), 6.99 (2H, d, *J* = 7.8 Hz, Ar-*H*), 6.67–6.65 (2H, m, CH=CH), 6.13 (1H, br s, O*H*) 4.63–4.62 (2H, m, NH-C*H*_2_), 3.67 (2H, t, *J* = 7.8 Hz, C*H*_2_), 2.98 (2H, t, *J* = 7.8 Hz, C*H*_2_). ^13^C-NMR (125 MHz, DMSO-*d_6_*): δ 166.4, 165.8, 165.5, 157.9, 145.8, 144.0, 142.1, 140.5, 136.7, 136.6, 136.6, 133.9, 132.9, 132.6, 131.9, 131.7, 131.6, 131.2, 131.0, 129.9, 129.3, 129.1, 128.9, 44.6, 43.7, 34.8.

*(E)-4-{2-[(4-[(2,3-Dihydroxypropyl)amino]-6-{[4-(3-hydroxystyryl)phenyl]amino}-1,3,5-triazin-2-yl)amino]ethyl}benzenesulfonamide* (**38**): 

Vanilla solid; yield 33%; mp 152–155 °C. IR (ATR): 3392, 3273 (OH, NH, NH_2_), 3075, 3055, 3073, 3011, 2990, 1679 (CH=CH), 1574 (C=C, C=N), 1331 (SO_2_NH_2_), 1264, 1188 (SO_2_NH_2_), 1028. ^1^H-NMR (500 MHz, DMSO-*d_6_*): δ 9.07 (3H, br s, N*H*), 7.81 (2H, d, *J* = 7.8 Hz, Ar-*H*), 7.75 (2H, d, *J* = 7.8 Hz, Ar-*H*), 7.70 (2H, d, *J* = 8.0 Hz, Ar-*H*), 7.64 (2H, d, *J* = 8.0 Hz, Ar-*H*), 7.34–7.31 (1H, m, Ar-*H*), 7.16 (1H, d, *J* = 16.1 Hz, CH=CH) 7.05–7.01 (3H, m, Ar-*H*), 6.89 (1H, d, *J* = 16.1 Hz, CH=CH), 6.69 (2H, br s, N*H*_2_), 6.17 (3H, br s, O*H*), 4.47–4.45 (2H, m, NH-C*H*_2_), 3.66 (2H, t, *J* = 7.8 Hz, C*H*_2_), 3.58–3.56 (3H, m, C*H*-OH, C*H*_2_-OH), 3.12 (2H, t, *J* = 7.8 Hz, C*H*_2_). ^13^C-NMR (125 MHz, DMSO-*d_6_*): δ 166.8, 166.8, 166.3, 158.3, 145.2, 143.9, 142.1, 139.3, 138.1, 131.1, 131.0, 130.9, 130.7, 122.7, 119.6, 119.5, 119.4, 116.8, 116.1, 69.2, 63.9, 44.1, 43.6, 34.9.

*(E)-4-{2-[(4-{[4-(3-Hydroxystyryl)phenyl]amino}-6-[(4-sulfamoylbenzyl)amino]-1,3,5-triazin-2-yl)amino]ethyl}benzenesulfonamide* (**40**): 

Ocher solid; yield 78%; mp 101–103 °C. IR (ATR): 3385, 3245 (OH, NH, NH_2_), 3075, 3054, 2999, 2991, 1685 (CH=CH), 1590, 1576, 1572 (C=C, C=N), 1334 (SO_2_NH_2_), 1238, 1160 (SO_2_NH_2_). ^1^H-NMR (500 MHz, DMSO-*d_6_*): δ 9.13 (3H, br s, N*H*), 7.81 (2H, d, *J* = 8.0 Hz, Ar-*H*), 7.79 (2H, d, *J* = 8.0 Hz, Ar-*H*), 7.71–7.69 (1H, m, Ar-*H*), 7.62 (2H, d, *J* = 8.0 Hz, Ar-*H*), 7.58–7.54 (3H, m, Ar-*H*), 7.48 (2H, d, *J* = 8.0 Hz, Ar-*H*), 7.32 (2H, d, *J* = 8.0 Hz, Ar-*H*), 7.28 (2H, d, *J* = 8.0 Hz, Ar-*H*), 7.02–7.00 (2H, m, CH=CH), 6.69 (4H, br s, N*H*_2_), 6.27 (1H, br s, O*H*), 4.58–4.56 (2H, m, NH-C*H*_2_), 3.69–3.67 (2H, m, C*H*_2_), 3.12–3.08 (2H, m, C*H*_2_). ^13^C-NMR (125 MHz, DMSO-*d_6_*): δ 166.7, 166.5, 166.3, 157.6 144.8, 144.6, 142.5, 141.9, 138.9, 137.6, 137.1, 134.4, 133.7, 133.3, 133.2, 132.8, 132.4, 132.1, 131.9, 131.2, 130.6, 130.5, 130.1, 45.1, 42.2, 34.4.

*(E)-4,4’-{[(6-{[4-(3-Hydroxystyryl)phenyl]amino}-1,3,5-triazin-2,4-diyl)bis(azandiyl)]bis(ethan-2,1-diyl)} benzenesulfonamide* (**41**): 

Vanilla solid; yield 52%; mp 94–96 °C. IR (ATR): 3373, 3296 (OH, NH, NH_2_), 3076, 3055, 3024, 2929, 1658 (CH=CH), 1560,1542 (C=C, C=N), 1331 (SO_2_NH_2_), 1275, 1182 (SO_2_NH_2_). ^1^H-NMR (500 MHz, DMSO-*d_6_*): δ 9.11 (7H, br s, N*H*, N*H*_2_), 7.29 (4H, d, *J* = 8.1 Hz, Ar-*H*), 7.06 (4H, d, *J* = 8.1 Hz, Ar-*H*), 6.98–6.97 (1H, m, Ar-*H*), 6.86 (2H, d, *J* = 8.0 Hz, Ar-*H*), 6.74–6.71 (3H, m, Ar-*H*), 6.64 (2H, d, *J* = 8.0 Hz, Ar-*H*), 6.61–6.59 (2H, m, CH=CH), 6.11 (1H, br s, O*H*), 3.57–3.54 (4H, m, NH-C*H*_2_), 2.91–2.87 (4H, m, C*H*_2_). ^13^C-NMR (125 MHz, DMSO-*d_6_*): δ 166.3, 166.2, 165.8, 158.2, 144.7, 144.4, 142.1, 141.3, 138.7, 135.1, 133.5, 133.4, 132.9, 129.4, 128.6, 128.4, 128.2, 127.6, 127.5, 43.6, 34.7.

*(E)-4-{2-[(4-[(2,3-Dihydroxypropyl)amino]-6-{[4-(4-hydroxystyryl)phenyl]amino}-1,3,5-triazin-2-yl)amino]ethyl}benzenesulfonamide* (**42**): 

Yellow solid; yield 83%; mp 129–130 °C. IR (ATR): 3363, 3352 (OH, NH, NH_2_), 3059, 3026, 2962, 2930, 1659 (CH=CH), 1594, 1543, 1520 (C=C, C=N), 1334 (SO_2_NH_2_), 1260, 1155 (SO_2_NH_2_), 1028. ^1^H-NMR (500 MHz, DMSO-*d_6_*): δ 9.14 (3H, br s, N*H*), 7.75 (2H, d, *J* = 7.8 Hz, Ar-*H*), 7.70 (2H, d, *J* = 7.8 Hz, Ar-*H*), 7.67 (2H, d, *J* = 8.0 Hz, Ar-*H*), 7.60 (2H, d, *J* = 8.0 Hz, Ar-*H*), 7.57 (2H, d, *J* = 8.0 Hz, Ar-*H*), 7.54 (2H, d, *J* = 8.0 Hz, Ar-*H*), 7.25 (1H, d, *J* = 16.1 Hz, CH=CH), 7.15 (1H, d, *J* = 16.1 Hz, CH=CH), 6.68 (2H, br s, N*H*_2_), 6.32 (3H, br s, O*H*), 4.62–4.61 (2H, m, NH-C*H*_2_), 3.65 (2H, t, *J* = 7.8 Hz, C*H*_2_), 3.32–3.29 (3H, m, C*H*-OH, C*H*_2_-OH) 2.80 (2H, t, *J* = 7.8 Hz, C*H*_2_). ^13^C-NMR (125 MHz, DMSO-*d_6_*): δ 166.9, 166.8, 166.2, 157.2, 143.8, 143.6, 142.1, 140.0, 139.9, 131.2, 129.3, 128.7, 127.2, 126.0, 120.9, 116.1, 116.0, 69.1, 63.8, 43.9, 43.5, 34.7.

*(E)-4-{2-[(4-{[4-(4-Hydroxystyryl)phenyl]amino}-6-[(4-sulfamoylbenzyl)amino]-1,3,5-triazin-2-yl)amino]ethyl}benzenesulfonamide* (**44**): 

Ocher solid; yield 70%; mp 81–82 °C. IR (ATR): 3371, 3365 (OH, NH, NH_2_), 3081, 3054, 3029, 2962, 2928, 1709 (CH=CH), 1659, 1632, 1561 (C=C, C=N), 1333 (SO_2_NH_2_), 1260, 1154 (SO_2_NH_2_). ^1^H-NMR (500 MHz, DMSO-*d_6_*): δ 9.21 (7H, br s, N*H*, N*H*_2_), 7.68 (2H, d, *J* = 7.8 Hz, Ar-*H*), 7.65 (2H, d, *J* = 7.8 Hz, Ar-*H*), 7.61 (2H, d, *J* = 8.0 Hz, Ar-*H*), 7.57 (2H, d, *J* = 8.0 Hz, Ar-*H*), 7.52 (2H, d, *J* = 8.0 Hz, Ar-*H*), 7.46 (2H, d, *J* = 7.8 Hz, Ar-*H*), 7.38 (2H, d, *J* = 7.8 Hz, Ar-*H*), 7.35 (2H, d, *J* = 8.0 Hz, Ar-*H*), 7.14–7.11 (2H, m, CH=CH), 6.17 (1H, br s, O*H*), 4.58–4.56 (2H, m, C*H*_2_), 3.62–3.60 (2H, m, C*H*_2_), 2.87–2.86 (2H, m, C*H*_2_). ^13^C-NMR (125 MHz, DMSO-*d_6_*): δ 165.9, 165.8, 165.7, 157.2, 143.2, 143.1, 142.8, 141.4, 138.6, 138.0, 137.8, 134.2, 133.8, 132.6, 132.1, 131.8, 131.4, 130.6, 130.5, 128.6, 128.0, 44.1, 42.9, 34.6. 

### 4.3. Carbonic Anhydrase Inhibition Assay

AnSX.18MV-R Applied Photophysics stopped-flow instrument (Applied Photophysics, Leatherhead, UK) has been used for assaying the CA catalyzed CO_2_ hydration activity [44]. Phenol red (at a concentration of 0.2 mM) has been used as an indicator, working at the absorbance maximum of 557 nm, with 20 mM Hepes (pH 7.5) as a buffer and 20 mM Na_2_SO_4_ (for maintaining constant the ionic strength), following the CA-catalyzed CO_2_ hydration reaction for a period of 10–100 s. The CO_2_ concentrations ranged from 1.7 to 17 mM for the determination of the kinetic parameters and inhibition constants. For each inhibitor, at least six traces of the initial 5–10% of the reaction were used for determining the initial velocity. The uncatalyzed rates were determined in the same manner and were subtracted from the total observed rates. Stock solutions of the inhibitor (0.1 mM) were prepared in distilled-deionized water, and dilutions up to 0.005 nM were done thereafter with the assay buffer. Inhibitor and enzyme solutions were preincubated together for 15 min at room temperature prior to assay in order to allow for the formation of the E–I complex. The inhibition constants were obtained by non-linear least-squares methods using PRISM 3 and the ChengPrusoff equation, as reported earlier, and represent the mean from at least three different determinations. All CA isoforms were recombinant ones and were prepared as reported earlier [59,60,61,62,63,64,65]. Errors in the range of 5–10% of the reported value (Mean from three different assays, by stopped-flow technique).

### 4.4. VRE Inhibition Assay

The inhibition activity against enterococci was evaluated by the microtitration broth method according to the Clinical and Laboratory Standards Institute with some modifications [66]. The compounds were dissolved in DMSO and serially diluted in Brain Hearth Infusion (BHI) to reach concentrations 256–2 µg/mL. The plate was inoculated with a tested microorganism (reference strain *E. faecalis* ATCC 29212 and clinical isolates VRE 342 B, VRE 368, and VRE 725B [50]. The plate was incubated at 37 °C for 24 h. After the incubation, the minimum inhibitory concentration was evaluated. A total of 20 µL of Alamar Blue solution was added to each well, and the plate was incubated 1 h at 37 °C. The minimum inhibitory concentration was evaluated as the lowest concentration of the tested compound, which fully inhibited the color change of Alamar Blue from blue to pink. Vancomycin and ampicillin were used as reference drugs. The experiment was repeated at least three times.

### 4.5. Cytotoxicity Determination against Human Colorectal Tumor Cell Line (HCT116 p53^+/+^)

#### 4.5.1. Cell Culture

The human colorectal tumor cell line (HCT116 p53^+/+^) was given by Dr. Vogelstein [67]. Cells were grown in a tissue culture flask 25 cm^2^ (TPP) in Dulbecco’s Modified Eagle Medium (DMEM) with high glucose 4.5 g/L, L-glutamine, and natrium pyruvate (Biosera). The medium was supplemented with 10 % fetal bovine serum (Biosera, Nuaille, France) and 100 μg/mL of penicillin/streptomycin (Biosera, Nuaille, France).

#### 4.5.2. MTT Assay

Cells were harvested using trypsin (Biosera, Nuaille, France) and were then seeded into the 10 cm tissue culture dish (TPP). The cells were harvested after 24 h during the exponential growth phase and were seeded into a tissue culture test plate with 96 wells (TPP) at a concentration of 7.5 × 10^3^ cells per well and incubated for 24 h. After this period, the growth medium was exchanged for a medium containing compounds in a concentration range from 0.1 to 50 μM. Stock solutions of the investigated compounds were prepared in DMSO (Sigma–Aldrich, Darmstadt, Germany). After 48 h of incubation with the investigated compounds, the medium was replaced with 100 μL of DMEM and 20 μL of MTT solution in each well. This solution was prepared by dissolving 2.5 mg of the Thiazolyl Blue Tetrazolium Bromide (Sigma–Aldrich, Darmstadt, Germany) per 1 mL 1 × PBS. After 2 h of incubation, the medium with MTT solution was replaced with isopropylalcohol (150 μL to each well) to dissolve the newly created formazan crystals. After 10 min, absorbance was measured using a Synergy H1 Hybride Multi-mode Microplate Reader (Bio Tek; Agilent Technologies, Santa Clara, CA, USA) at 595 nm. The inhibitory concentration (IC_50_) was defined as the concentration of the investigated compounds that was necessary to reduce the metabolic activity of cells to 50 % of the untreated control cells, and it was expressed as means ± standard deviation (SD) in software GraphPadPrism 5 (GraphPad Software, San Diego, CA, USA). Each individual compound was tested in triplicate and repeated three times.

### 4.6. Molecular Modeling

#### 4.6.1. Molecular Docking into hCA IX

The crystal structure (PDB: 3IAI [68]) of hCA IX was prepared via protein preparation wizard [69] in Maestro 12.7 (Schrödinger, Inc., Mannheim, Germany). Bond orders were assigned. All missing hydrogen atoms were added. As the hCA IX is a homotetramer, the B, C, and D chains were removed. The structure was optimized for pH 7.0 using PROPKA. All water molecules were removed. Finally, the protein was minimized using the OPLS3e force field [70], and a grid for docking was generated. 

Using the CombiGlide module of Schrödinger Suite (Schrödinger, Inc., Mannheim, Germany), a small virtual combinatorial library of 76 compounds was prepared (Supplementary Materials). Ligands were prepared using the LigPrep module in Maestro. In order to obtain correct molecular geometries and protonation states at pH 7.0 ± 2.0, Epik module and OPLS3e force field [70] were used. Metal-binding states were added.

Docking was performed in the program Schrodinger Glide [71] with XP precision for all molecules. Penalization for a low probable ionization state was used. As it is well known that sulfonamide bound to CAs create a metal-coordination bond between sulfonamide nitrogen and zinc dication, and that this strong type of interaction is not in the parametrization of the scoring function, the constraint to preserve the metal-coordination bond between zinc dication and deprotonated sulfonamide group was specified. A sampling of the nitrogen inversions and ring conformation was allowed. Relative binding affinities with optimization of poses and residues of amino acids within 5 Å for all docked poses were predicted for the most interesting ligands by Prime/MM-GBSA method [72,73].

#### 4.6.2. Retrieval of Enterococcal CA Protein Sequence

*E. faecalis* contains an α-CA and a γ-CA. The γ-CAs have a much smaller active site cavity and are much less efficient catalyst [6], making the α-CA a probable target of studied sulfonamides. The spatial structure of enterococcal α-CA was not resolved by now, therefore homology modeling of the enzyme was needed. The sequence of the α-CA from E. faecalis ATCC 29212, which was used in the screening, was retrieved from the NCBI database [74] with accession number OOC96771 and 232 amino acid length [75].

#### 4.6.3. 3D Homology Modeling of Enterococcal CA

The suitable template for homology modeling was identified in PDB using their advanced search to identify the highest sequence identity protein. The 3D model of the enterococcal α-CA was built using Prime in Schrodinger Suite 2018-4 [72,73] using the identified protein as a template. The target and template sequences were aligned using the Clustal W method in Prime. The overall stability of the protein was assessed using the Ramachandran plot [76].

The nontemplate loops (residues 44–46 and 159–164) were refined using OPLS 3e [70] in a water environment using the variable-dielectric generalized Born model [77], which incorporates residue-dependent effects. 

#### 4.6.4. Molecular Dynamic (MD) Simulation of Enterococcal CA

Structure **32** (with the lowest measured MIC against E. faecalis) was docked into the active site of the modeled 3D structure of the enterococcal α-CA using Glide with XP protocol, and with the best pose found was further optimized using MD simulations.

Molecular dynamics and molecular mechanics studies were designed and performed utilizing Gromacs simulation suite, version 2018.5. Initial structural inspections and visualizations were administered to Pymol 2.0.7 and Visual Molecular Dynamics (VMD). Molecular dynamics simulation of enterococcal protein was carried out with the inclusion of an explicit TIP3P water model in order to enable relaxation of structure required for the further production stage of analysis. We also performed MD equilibration and production runs for enzyme structure in a vacuum (without any solvent). Atomistic simulations and minimizations were carried out under the potential field of a CHARMM36 all-atom forcefield, which is suitable for modeling interactions regarding metalloproteins [78].

**Figure 15:**
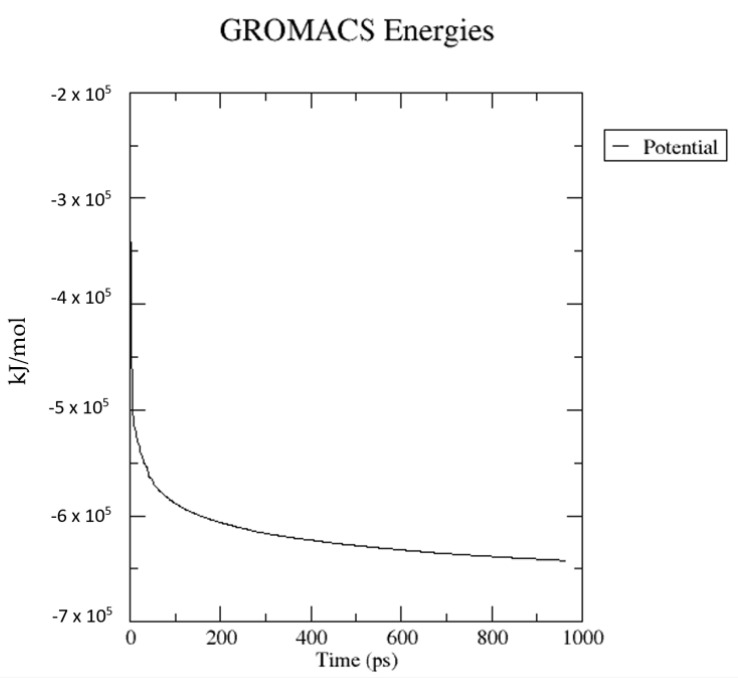
Energy minimization progress. The graph illustrates the descent of potential energy of receptor + solvent system as steepest descent algorithm is applied with the aforementioned conditions.

Prior to MD simulation, geometry optimization was carried out to avoid steric clashes and to lead the system to local minima. The structure of the protein was confined to periodic boundary conditions of the dodecahedron box with 10,636 water molecules and 8 chlorine atoms to balance the net charge of the system. The distance of the receptor of 2 nm from each box boundary is maintained to prevent protein self-interaction. Geometry optimization protocol consisted of the steepest descent algorithm with at least 1000 kcal/mol/nm maximum force necessary to stop minimization and optimization step of 0.01. The maximum number of minimization steps was set to 5000. The optimization process is depicted in Figure 15.

**Figure 16:**
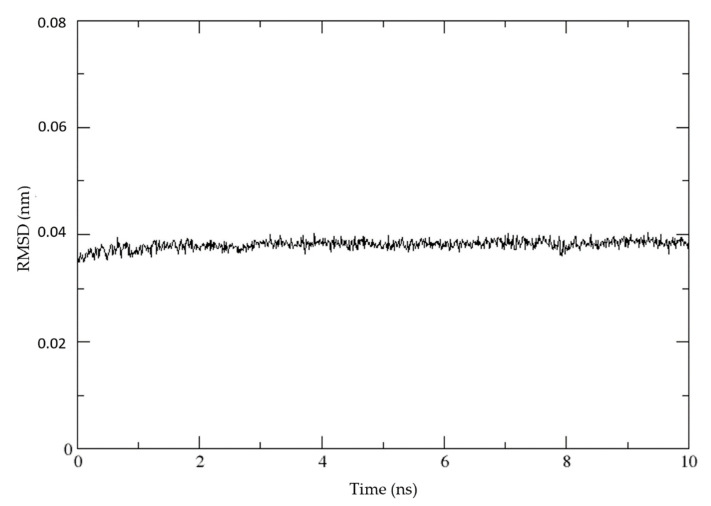
RMSD of whole protein structure after 10 ns production run. The whole 10 ns portion of the run was used for analysis. Only marginal variation of RMSD during 10 ns around the mean value of ~0.035 nm can be observed, suggesting the stability of the receptor structure after NVT equilibration run and within NPT production MD run.

Following the energy minimization, we approached to MD simulations with initial 500 ps equilibration in NVT ensemble, where N—number of particles, V—volume, and T—temperature were conserved. The production stage of 10 ns simulation was subjected to NPT ensemble (N—number of particles, P—pressure, and T—temperature where conserved). According to the analysis of the root mean square deviation (RMSD) of the protein structure, we decided to stop the production phase after 10 ns. As one can see in Figure 16, RMSD oscillates around the mean value.

A portion of the final 1 ns trajectory was further utilized for analysis. The integration time step was set to 2 fs, and every 1 ps frame of calculated trajectory was stored. Leap-frog integrator was employed with NVT and also an NPT production run ensemble. The periodic boundary conditions (PBC) were applied isotropically in all directions. The receptor and solvent molecules were thermostated at 298 K by the Parrinello–Rahman velocity rescale algorithm [79]. Isotropic pressure was applied by the Berendsen algorithm with an equilibrium pressure of 1 bar with a time constant of 3.0 ps and compressibility of 4.5 × 10^−5^ bar^−1^ [80]. The short-range cut-off for Lennard-Jones and electrostatic interactions were regulated to 10 Å. The long-range interactions with the reciprocal-space interactions evaluated on a 0.16 nm spacing grid and with cubic interpolation of fourth-order were treated by particle mesh Ewald (PME) method. LINCS algorithm was employed to constraint all bond lengths [81]. The 3D representation of prepared protein structure is in Figure 4.

#### 4.6.5. Induced Fit Docking to Enterococcal CA

The five compounds most active against *E. faecalis* (**7**, **9**, **21**, **25**, **32**) were used in the molecular docking exploiting Induced Fit Docking (IFD) protocol of Schrödinger Suite [82]. The IFD protocol involves the use of both the Glide docking program [71] and the Prime [72,73], a module for protein structure modeling. We chose the IFD procedure to implement flexibility of protein, as we did not have enough information about the conformation of the binding site amino acid residues when inhibitors are bound for this protein. No sidechains of the protein homology model were trimmed, just the van der Waals radii of ligand and protein atoms were scaled to half of their normal size during initial docking. The ligand molecules were prepared for IFD using LigPrep to generate all possible tautomers, enantiomers, and ionization states at pH 7.0 ± 2.0 using Epik [83,84]. All molecules are deprotonated on the sulfonamide group as it is well known that primary sulfonamide creates a complex bond with zinc dication (Zn^2+^) through the deprotonated amine of the sulfonamide group [85]. Afterward, were all generated molecules minimized using the OPLS 3e forcefield [70]. The best pose is chosen based on the IFD score calculated according to the following formula:

IFDScore = 1.0 ∗ Prime_Energy + 9.057 ∗ GlideScore + 1.428 ∗ Glide_Ecoul, where Prime_Energy is the energy of protein calculated with Prime, GlideScore is the score calculated with docking module Glide, and Glide_Ecoul is the Coulomb term of the GlideScore.

#### 4.6.6. Binding Energy Calculation with Enterococcal CA

The binding free energies of top poses obtained from IFD output were carried out by using Prime-MM/GBSA (molecular mechanics generalized Born surface area). Prime MM/GBSA includes the OPLS 3e force field [70], VSGB solvent model [77], and rotamer searching algorithms. The MM/GBSA calculations are used to estimate the relative binding affinity of ligands to the binding site of the protein (reported in kcal/mol). As the MM/GBSA binding energies are approximate free energies of binding, a more negative value indicates stronger binding.

#### 4.6.7. Prediction of ADMET and Fluorescence

Except for finding important structural features for binding with the target protein, it was in our interest to look for the ADMET properties of the most promising compounds. Schrodinger QikProp was used to calculate the ADMET profile for the five *s*-triazine analogues, and GLORY [86,87] was used to additional predictions of metabolism.

Stilbenes and especially chalcones are known to have fluorescent properties. Prediction tool ChemFLuo [88] was used to predict the probability of the compounds to possess green or blue fluorescence.

## Figures and Tables

**Table 1:** Synthesis of chalcone precursors. Comparison of reaction times and %yields of reactions catalyzed by H_2_SO_4_, Ce(III) supp., and catalyzed by binary catalytic system H_2_SO_4_ /Ce(III) supp. (Reaction conditions—1 eq of aminoacetophenone, 1 eq of appropriate benzaldehyde, appropriate catalytic system, methanol, reflux.).

Compound	Catalytic System					
	H_2_SO_4_^a^		Ce(III) Supp. ^b^		H_2_SO_4_^a^ /Ce(III) Supp. ^b^	
	Time (h)	%Yield	Time (h)	%Yield	Time (h)	%Yield
**A**	40	87	38	85	12	88
**B**	24	90	24	83	6	89
**C**	40	72	36	72	8	83
**D**	36	81	34	82	10	93

^a^ Catalytic amount of H_2_SO_4_. ^b^ 5 mol% Ce(III) supp. at the cation exchanger.

**Table 2:** Inhibition data for synthesized *s*-triazine derivatives **1**–**44** (for the structure see Scheme 2) and standard sulfonamide CA inhibitors against hCA I, hCA II, hCA VII, hCA IX, and hCA XII; their selectivity ratios for inhibition of isozyme hCA VII over hCA II, hCA IX over hCA II, and hCA XII over hCA II. For the evaluation, a methodology based on stopped-flow assay was employed [45]; three different assays for each compound; see Section 4.3.

Comp.	n	R^1^	R^2^ or R^3^	*K*_I_ (nM) ^a^					Selectivity Ratio		
				hCA I	hCA II	hCA VII	hCA IX	hCA XII	K_I_ (hCA II)/K_I_ (hCA VII)	K_I_ (hCA II) /K_I_ (hCA IX)	K_I_ (hCA II) /K_I_ (hCA XII)
**1**	0	Cl	NH-CH_2_CH_2_CH_2_OH	26.8	44.8	127.7	228.9	NT	0.35	0.19	NT
**2**	0	Cl	NH-CH_2_CH_2_-C_6_H_4_(1,4)-4-SO_2_NH_2_	388.5 ^e^	41.8 ^e^	NT	28.0 ^e^	NT	NT	1.49 ^e^	NT
**3**	2	NH-C_6_H_4_(1,4)-4-SO_2_NH_2_	NH-CH_2_CH_2_-C_6_H_4_(1,4)-4-SO_2_NH_2_	**8.5** ^e^	**4.8** ^e^	NT	**2.0** ^e^	NT	NT	**2.40** ^e^	NT
**4**	0	NH-CH_2_CH_2_CH_2_OH	3-OH	7523	3640	NT	27.5	65.8	NT	**132.36**	**55.32**
**5**	1	Cl	NH-CH_2_CH_2_CH_2_OH	3534 ^e^	57.4 ^e^	331.5	635.1 ^e^	NT	0.17	0.09 ^e^	NT
**6**	1	Cl	NH-C_6_H_4_(1,4)-4-OH	**16.7** ^e^	**7.4** ^e^	NT	**0.4** ^e^	NT	NT	**18.50** ^e^	NT
**7**	1	Cl	NH-CH_2_-C_6_H_4_(1,4)-4-SO_2_NH_2_	731.3 ^e^	75.9 ^e^	NT	307.7 ^e^	NT	NT	0.25 ^e^	NT
**8**	1	Cl	NH-CH_2_CH_2_-C_6_H_4_(1,4)-4-SO_2_NH_2_	391.7 ^e^	**18.5** ^e^	NT	190.0 ^e^	NT	NT	0.10 ^e^	NT
**9**	1	NH-C_6_H_4_(1,4)-4-OH	NH-CH_2_CH(OH)CH_2_OH	491.5 ^e^	66.9 ^e^	NT	32.8 ^e^	NT	NT	2.04 ^e^	NT
**10**	1	NH-CH_2_-C_6_H_4_(1,4)-4-SO_2_NH_2_	NH-CH_2_CH(OH)CH_2_OH	76.1 ^e^	**5.1** ^e^	NT	**1.0** ^e^	NT	NT	**5.10** ^e^	NT
**11**	1	NH-CH_2_-C_6_H_4_(1,4)-4-SO_2_NH_2_	NH-CH_2_CH_2_-C_6_H_4_(1,4)-4-SO_2_NH_2_	313.5 ^e^	**7.8** ^e^	NT	**2.4** ^e^	NT	NT	**3.25** ^e^	NT
**12**	2	NH-CH_2_-C_6_H_4_(1,4)-4-SO_2_NH_2_	NH-CH_2_CH_2_-C_6_H_4_(1,4)-4-SO_2_NH_2_	62.3 ^e^	**5.6** ^e^	NT	**1.6** ^e^	NT	NT	**3.50** ^e^	NT
**13**	1	NH-CH_2_CH_2_CH_2_OH	-H	1960	277.4	95.7	4626	NT	2.90	0.06	NT
**14**	1	NH-CH_2_CH_2_CH_2_OH	2-OH	5399	**35.4**	180.4	622.7	NT	0.20	0.06	NT
**15**	1	NH-CH_2_CH_2_CH_2_OH	3-OH	>10,000	3017	**25.9**	>10,000	NT	**116.48**	0.30	NT
**16**	1	NH-CH_2_CH_2_CH_2_OH	4-OH	799.9	**36.0**	201.0	160.9	NT	0.18	0.23	NT
**17**	1	NH-CH_2_CH_2_CH_2_OH	3-OH	358.2	46.8	NT	**22.2**	492.9	NT	2.11	0.09
**18**	2	Cl	NH-CH_2_CH_2_CH_2_OH	52.0 ^e^	36.1 ^e^	75.7	231.2 ^e^	NT	0.48	0.16 ^e^	NT
**19**	2	Cl	NH-CH_2_CH(OH)CH_2_OH	9290 ^e^	78.0 ^e^	40.7	646.9 ^e^	NT	1.92	0.12 ^e^	NT
**20**	2	Cl	NH-C_6_H_4_(1,4)-4-OH	462.0 ^e^	65.5 ^e^	NT	192.0 ^e^	NT	NT	0.34 ^e^	NT
**21**	2	NH-C_6_H_4_(1,4)-4-OH	-H	>10,000	3158	810.2	>10,000	NT	3.90	0.32	NT
**22**	2	NH-CH_2_CH_2_CH_2_OH	2-OH	7703	938.9	283.5	>10,000	NT	3.32	0.09	NT
**23**	2	NH-CH_2_CH(OH)CH_2_OH	2-OH	>10,000	83.0	88.7	>10,000	NT	0.94	0.01	NT
**24**	2	NH-C_6_H_4_(1,4)-4-OH	2-OH	5013	**63.8**	280.8	227.4	NT	0.23	0.28	NT
**25**	2	NH-CH_2_CH_2_CH_2_OH	3-OH	7487	4128	185.8	>10,000	NT	25.99	0.41	NT
**26**	2	NH-C_6_H_4_(1,4)-4-OH	3-OH	6627	1335	**42.1**	>10,000	NT	**31.71**	0.13	NT
**27**	2	NH-CH_2_CH_2_CH_2_OH	4-OH	8958	**28.8**	458.6	>10,000	NT	0.06	0.00	NT
**28**	2	NH-CH_2_CH(OH)CH_2_OH	4-OH	8223	184.4	594.2	8461	NT	0.31	0.02	NT
**29**	2	NH-C_6_H_4_(1,4)-4-OH	4-OH	5806	730.4	88.6	>10,000	NT	8.24	0.07	NT
**30**	2	NH-CH_2_CH_2_CH_2_OH	-H	5697	5133	NT	**12.1**	**7.6**	NT	**424.21**	**675.39**
**31**	2	NH-CH_2_CH(OH)CH_2_OH	-H	**36.9**	335.5	NT	248.3	**4.4**	NT	1.35	**76.25**
**32**	2	NH-C_6_H_4_(1,4)-4-OH	-H	90.6	7488	NT	2468	**5.9**	NT	3.03	**1269.15**
**33**	1	NH-CH_2_CH_2_-C_6_H_4_(1,4)-4-SO_2_NH_2_	-H	48.0	57.9	NT	1967	**6.2**	NT	0.03	9.34
**34**	2	NH-CH_2_CH(OH)CH_2_OH	2-OH	1672	5632	NT	2713	**8.5**	NT	2.08	**662.59**
**35**	2	NH-C_6_H_4_(1,4)-4-OH	2-OH	3937	5470	NT	238.5	37.1	NT	**22.94**	**147.44**
**36**	1	NH-CH_2_CH_2_-C_6_H_4_(1,4)-4-SO_2_NH_2_	2-OH	447.5	82.0	NT	2722	**8.3**	NT	0.03	9.88
**37**	2	NH-CH_2_CH_2_CH_2_OH	3-OH	675.3	73.5	NT	26.4	601.2	NT	2.78	0.12
**38**	2	NH-CH_2_CH(OH)CH_2_OH	3-OH	6726	4443	NT	**17.3**	84.5	NT	**256.82**	**52.58**
**39**	2	NH-C_6_H_4_(1,4)-4-OH	3-OH	742.5	154.1	NT	876.0	68.5	NT	0.17	2.25
**40**	1	NH-CH_2_CH_2_-C_6_H_4_(1,4)-4-SO_2_NH_2_	3-OH	48.8	**18.9**	NT	**20.3**	60.5	NT	0.93	0.31
**41**	2	NH-CH_2_CH_2_-C_6_H_4_(1,4)-4-SO_2_NH_2_	3-OH	608.2	41.4	NT	31.1	62.2	NT	1.33	0.67
**42**	2	NH-CH_2_CH(OH)CH_2_OH	4-OH	43.5	96.3	NT	258.0	**9.7**	NT	0.37	9.93
**43**	2	NH-C_6_H_4_(1,4)-4-OH	4-OH	4061	4837	NT	25.7	702.5	NT	**188.21**	6.88
**44**	1	NH-CH_2_CH_2_-C_6_H_4_(1,4)-4-SO_2_NH_2_	4-OH	858.9	252.0	NT	249.3	**10.0**	NT	1.01	**25.2**
**AAZ**	-	-	-	250.0 ^b^	12.1 ^b^	2.5 ^b^	25.8 ^b^	5.7	4.84	0.47	2.13
**BRZ**	-	-	-	NT	3.0 ^b^	2.8 ^c^	37.0 ^b^	NT	1.07	0.08	NT
**DCP**	-	-	-	1200.0 ^b^	38.0 ^b^	26.5 ^c^	50.0 ^b^	50.0 ^d^	1.44	0.76	0.76
**DZA**	-	-	-	50 000.0^b^	9.0 ^b^	3.5 ^c^	52.0 ^b^	NT	2.57	0.17	NT
**EZA**	-	-	-	25.0 ^b^	8.0 ^b^	0.8 ^c^	34.0 ^b^	22.0 ^d^	10.26	0.23	0.36
**IND**	-	-	-	31.0 ^b^	15.0 ^b^	NT	24.0 ^b^	3.4 ^d^	NT	0.62	4.41
**MZA**	-	-	-	50.0 ^b^	14.0 ^b^	2.1 ^c^	27.0 ^b^	3.4 ^d^	6.67	0.52	4.12

NT = not tested. ^a^ Errors in the range of 5–10% of the reported value (Mean from three different assays, by stopped-flow technique). ^b^ [46]; ^c^ [47]; ^d^ [48]; ^e^ [39].

**Table 3:** Data of antimicrobial activity (MICs) against *E. faecalis* (EF) and three vancomycin-resistant strains of VRE in comparison with standard ampicillin (AMP) and vancomycin (VAN). The inhibition activity against enterococci was evaluated by the microtitration broth method, according to CLSI, with some modifications; the experiment was repeated at least three times; see Section 4.4.

Comp.	n	R^1^	R^2^ or R^3^	MIC [µM]			
				EF	VRE 342 B	VRE 368	VRE 725 B
**1**	0	Cl	NH-CH_2_CH_2_CH_2_OH	>713	>713	>713	>713
**2 ^a^**	0	Cl	NH-CH_2_CH_2_-C_6_H_4_(1,4)-4-SO_2_NH_2_	132	>528	>528	>528
**3 ^a^**	2	NH-C_6_H_4_(1,4)-4-SO_2_NH_2_	NH-CH_2_CH_2_-C_6_H_4_(1,4)-4-SO_2_NH_2_	>395	>395	>395	>395
**4**	0	NH-CH_2_CH_2_CH_2_OH	3-OH	>479	>479	>479	>479
**5 ^a^**	1	Cl	NH-CH_2_CH_2_CH_2_OH	343	343	343	686
**6 ^a^**	1	Cl	NH-C_6_H_4_(1,4)-4-OH	157	157	314	157
**7 ^a^**	1	Cl	NH-CH_2_-C_6_H_4_(1,4)-4-SO_2_NH_2_	16.49	65.98	32.99	131
**8 ^a^**	1	Cl	NH-CH_2_CH_2_-C_6_H_4_(1,4)-4-SO_2_NH_2_	128	128	64.26	>514
**9 ^a^**	1	NH-C_6_H_4_(1,4)-4-OH	NH-CH_2_CH(OH)CH_2_OH	34.67	34.67	69.34	34.67
**10 ^a^**	1	NH-CH_2_-C_6_H_4_(1,4)-4-SO_2_NH_2_	NH-CH_2_CH(OH)CH_2_OH	>475	>475	>475	>475
**11 ^a^**	1	NH-CH_2_-C_6_H_4_(1,4)-4-SO_2_NH_2_	NH-CH_2_CH_2_-C_6_H_4_(1,4)-4-SO_2_NH_2_	197	98.80	197	98.80
**12 ^a^**	2	NH-CH_2_-C_6_H_4_(1,4)-4-SO_2_NH_2_	NH-CH_2_CH_2_-C_6_H_4_(1,4)-4-SO_2_NH_2_	>387	>387	>387	>387
**13 ^b^**	1	NH-CH_2_CH_2_CH_2_OH	-H	228	228	114	228
**14 ^b^**	1	NH-CH_2_CH_2_CH_2_OH	2-OH	111	111	111	>444
**15 ^b^**	1	NH-CH_2_CH_2_CH_2_OH	3-OH	222	222	111	111
**16**	1	NH-CH_2_CH_2_CH_2_OH	4-OH	>444	>444	>444	>444
**17 ^b^**	1	NH-CH_2_CH_2_CH_2_OH	3-OH	>467	>467	467	>467
**18 ^a^**	2	Cl	NH-CH_2_CH_2_CH_2_OH	330	330	661	661
**19 ^a^**	2	Cl	NH-CH_2_CH(OH)CH_2_OH	>635	>635	>635	>635
**20 ^a^**	2	Cl	NH-C_6_H_4_(1,4)-4-OH	38.01	76.03	38.01	>608
**21**	2	NH-C_6_H_4_(1,4)-4-OH	-H	26.33	26.33	26.33	26.33
**22 ^b^**	2	NH-CH_2_CH_2_CH_2_OH	2-OH	108	108	108	108
**23 ^b^**	2	NH-CH_2_CH(OH)CH_2_OH	2-OH	52.83	52.83	52.83	105
**24 ^b^**	2	NH-C_6_H_4_(1,4)-4-OH	2-OH	51.30	51.30	51.30	102
**25**	2	NH-CH_2_CH_2_CH_2_OH	3-OH	27.13	27.13	27.13	108
**26**	2	NH-C_6_H_4_(1,4)-4-OH	3-OH	51.30	51.30	25.65	51.30
**27**	2	NH-CH_2_CH_2_CH_2_OH	4-OH	>434	>434	>434	434
**28**	2	NH-CH_2_CH(OH)CH_2_OH	4-OH	>444	>444	>444	>444
**29**	2	NH-C_6_H_4_(1,4)-4-OH	4-OH	25.65	25.65	102	51.30
**30**	2	NH-CH_2_CH_2_CH_2_OH	-H	>469	>469	>469	>469
**31**	2	NH-CH_2_CH(OH)CH_2_OH	-H	>455	>455	>455	>455
**32**	2	NH-C_6_H_4_(1,4)-4-OH	-H	13.80	27.60	27.60	55.20
**33**	1	NH-CH_2_CH_2_-C_6_H_4_(1,4)-4-SO_2_NH_2_	-H	97.44	194	389	194
**34**	2	NH-CH_2_CH(OH)CH_2_OH	2-OH	>443	>443	>443	>443
**35 ^b^**	2	NH-C_6_H_4_(1,4)-4-OH	2-OH	>429	>429	>429	>429
**36**	1	NH-CH_2_CH_2_-C_6_H_4_(1,4)-4-SO_2_NH_2_	2-OH	>380	>380	>380	>380
**37 ^b^**	2	NH-CH_2_CH_2_CH_2_OH	3-OH	>455	>455	>455	>455
**38**	2	NH-CH_2_CH(OH)CH_2_OH	3-OH	>443	>443	>443	>443
**39 ^b^**	2	NH-C_6_H_4_(1,4)-4-OH	3-OH	>429	>429	>429	>429
**40**	1	NH-CH_2_CH_2_-C_6_H_4_(1,4)-4-SO_2_NH_2_	3-OH	190	190	380	190
**41**	2	NH-CH_2_CH_2_-C_6_H_4_(1,4)-4-SO_2_NH_2_	3-OH	372	>372	>372	>372
**42**	2	NH-CH_2_CH(OH)CH_2_OH	4-OH	55.39	110	221	221
**43 ^b^**	2	NH-C_6_H_4_(1,4)-4-OH	4-OH	429	>429	>429	>429
**44**	1	NH-CH_2_CH_2_-C_6_H_4_(1,4)-4-SO_2_NH_2_	4-OH	380	190	>380	380
**AMP**	–	–	–	**11.5**	**11.5**	**11.5**	**5.72**
**VAN**	–	–	–	–	353	353	706

^a^ [39]; ^b^ [40].

**Table 4:** Cytotoxicity data against Human Colorectal Tumour Cell Line (HCT116 p53^+/+^). The cytotoxicity evaluation was performed only for compounds with promising inhibitory activity against hCA IX, hCA XII, or anti-VRE activity. For the evaluation, MTT assay was employed; each individual compound was tested in triplicate and repeated three times; see Section 4.5.

Comp.	n	R^1^	R^2^ or R^3^	Tox IC_50_ [µM]
**7 ^a^**	1	Cl	NH-CH_2_-C_6_H_4_(1,4)-4-SO_2_NH_2_	6.19 ± 2.15
**9 ^a^**	1	NH-C_6_H_4_(1,4)-4-OH	NH-CH_2_CH(OH)CH_2_OH	> 50
**20 ^a^**	2	Cl	NH-C_6_H_4_(1,4)-4-OH	> 50
**21**	2	NH-C_6_H_4_(1,4)-4-OH	-H	> 50
**24 ^b^**	2	NH-C_6_H_4_(1,4)-4-OH	2-OH	> 50
**25**	2	NH-CH_2_CH_2_CH_2_OH	3-OH	> 50
**26**	2	NH-C_6_H_4_(1,4)-4-OH	3-OH	> 50
**29**	2	NH-C_6_H_4_(1,4)-4-OH	4-OH	> 50
**32**	2	NH-C_6_H_4_(1,4)-4-OH	-H	6.51 ± 1.84
**DOX**				0.09 ± 1.12

^a^ [39]. ^b^ [40].

**Table 5:** Computed binding scores of selected ligands docked to the hCA IX.

Ligand	*K*_I_ (hCA IX) [nM]	Docking Score [kcal/mol]	E (MM/GBSA) [kcal/mol]
**4**	27.5	−8.612	−55.08
**17**	22.2	−8.499	−67.42
**30**	12.1	−7.124	−69.19
**37**	26.4	−7.627	−75.03
**38**	17.3	−7.664	−90.82
**40**	20.3	−7.068	−59.25
**41**	31.1	−7.088	−65.38
**43**	25.7	−6.958	−78.89

**Table 6:** Computed binding scores of selected ligands docked to the enterococcal α-CA homology model.

Ligand	MIC (*E. faecalis*) [µM]	Docking Score [kcal/mol]	IFD Score [kcal/mol]	E (MM/GBSA) [kcal/mol]
**7**	16.49	−7.324	3502.93	−51.59
**9 S-enantiomer**	34.67	−7.930	3489.76	−60.74
**9 R-enantiomer**		−7.539	3497.22	−53.54
**21**	26.33	−10.013	3483.60	−69.33
**25**	27.13	−9.829	3492.28	−57.21
**32**	13.80	−9.126	3495.30	−75.35

**Table 7:** Predicted octanol/water partition coefficient (QPlogP_oct/w_), water solubility (QPlogS), peroral absorption in %, affinity to Human ether-a-go-go-related gene (QPlogHERG), and brain/blood partition coefficient (QPlogBB). All except oral absorption are decadic logarithms of molar concentration.

Ligand	QPlogP_oct/w_	QPlogS	Oral Absorp.%	QPlogHERG	QPlogBB
**7**	−0.1	−4.3	14	−6.6	−3.7
**9 S-enantiomer**	−0.5	−3.4	11	−6.8	−4.2
**9 R-enantiomer**	−0.7	−3.4	7	−6.9	−4.5
**21**	3.5	−7.3	27	−9.1	−4.5
**25**	2.2	−6.0	17	−8.2	−4.8
**26**	2.5	−6.5	11	−8.7	−5.2
**32**	4.5	−7.8	58	−9.4	−3.5

**Table 8:** Prediction of green (G) and blue (B) fluorescence.

Ligand	Probability-G	Probability-B
**7**	0.51267	0.0162054
**9**	0.249959	0.00907222
**21**	0.73961	0.02375
**25**	0.641677	0.0461065
**26**	0.688407	0.0478596
**32**	0.649607	0.0226621

## Data Availability

Not applicable.

## Supplementary Materials

The following are available online at https://www.mdpi.com/article/10.3390/ijms23010231/s1.

## Abbreviations

AAZ	acetazolamide
Absorp.	absorption
ADMET	absorption, distribution, metabolism, excretion, and toxicity
AMP	ampicillin
BHI	brain hearth infusion
BRZ	brinzolamide
CA	carbonic anhydrase
cat.	catalyst
CLSI	Clinical and Laboratory Standards Institute
CNS	central neural system
Comp.	compound
DCP	dichlorphenamide
DMEM	Dulbecco’s modified Eagle medium
DMF	*N,N*-dimethylformamide
DMSO	dimethyl sulfoxide
DOX	doxorubicin
DZA	dorzolamide
*E. faecalis*	*Enterococcus faecalis*
*E. faecium*	*Enterococcus faecium*
EMA	European Medicines Agency
EZA	ethoxzolamide
FDA	Food and Drug Administration
FTIR	Fourier transform infrared spectroscopy
hCA	human carbonic anhydrase
HERG	human ether-a-go-go-related gene
IFD	induced-fit docking
IND	indisulam
MD	molecular dynamic
MIC	minimum inhibitory concentration
MM/GBSA	molecular mechanics generalized Born surface area
MRSA	methicillin-resistant *S. aureus*
MTT	methylthiazolyldiphenyl-tetrazolium bromide
MZA	methazolamide
NMR	nuclear magnetic resonance
NT	not tested
PBC	periodic boundary conditions
PBS	phosphate buffered saline
PDB	protein data bank
PME	particle mesh Ewald
RMSD	root mean square deviation
*S. aureus*	*Staphylococcus aureus*
sp.	species
supp.	supported
TLC	thin-layer chromatography
TMS	tetramethylsilane
TOX	toxicity
VAN	vancomycin
VMD	visual molecular dynamics
VRE	vancomycin-resistant enterococci
WHO	World Health Organization
