# Supplementary material for: Novel 1,3,5-Triazinyl Aminobenzenesulfonamides Incorporating Aminoalcohol, Aminochalcone and Aminostilbene Structural Motifs as Potent Anti-VRE Agents, and Carbonic Anhydrases I, II, VII, IX, and XII Inhibitors
Source: Int J Mol Sci. 2021 Dec 26;23(1):231. doi: 10.3390/ijms23010231 (PMC8745223; doi:10.3390/ijms23010231)
Supplement: Supplementary file 1 [file ijms-23-00231-s001.zip › Docking against hCAIX.pdf]

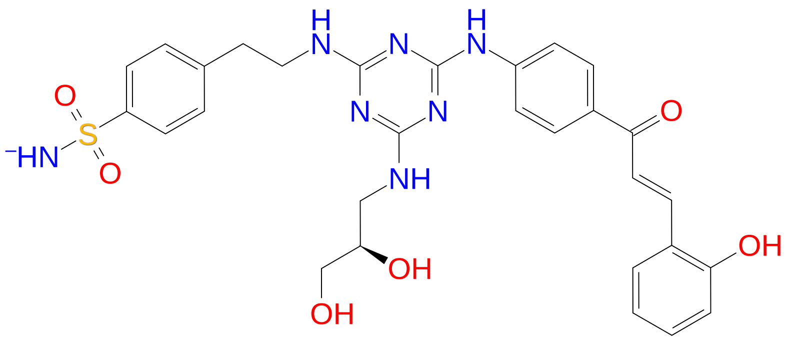

title: kyanurchlorid; [4-(2-aminoetyl)benzensulfonamid]; [3]; [o-hydroxychalcone]
docking score: -10.993

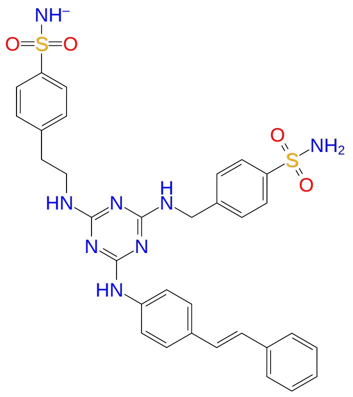

title: kyanurchlorid; [homosulfanilamid]; [4-(2-aminoetyl)benzensulfonamid]; [stilbene]
docking score: -10.483

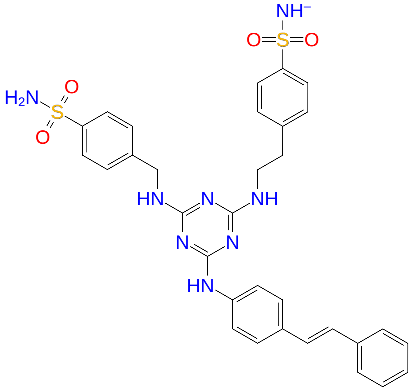

title: kyanurchlorid; [4-(2-aminoetyl)benzensulfonamid]; [homosulfanilamid]; [stilbene]
docking score: -10.483

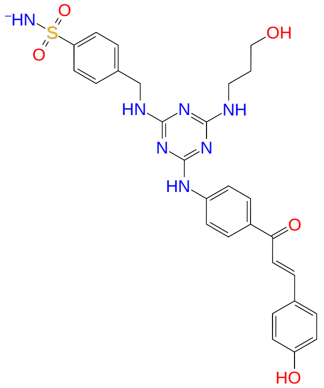

title: kyanurchlorid; [homosulfanilamid]; [2]; [p-hydroxychalcone]
docking score: -10.399

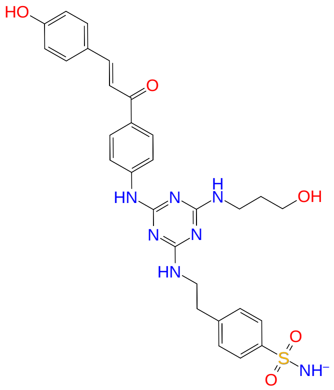

title: kyanurchlorid; [4-(2-aminoetyl)benzensulfonamid]; [2]; [p-hydroxychalcone]
docking score: -10.281

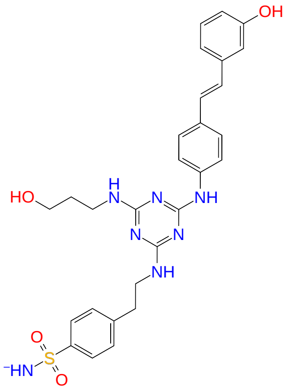

title: kyanurchlorid; [4-(2-aminoetyl)benzensulfonamid]; [2]; [m-hydroxystilbene]
docking score: -10.272

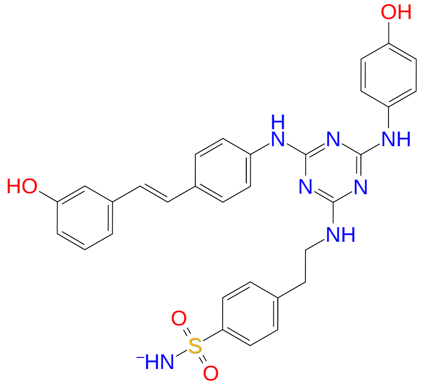

title: kyanurchlorid; [4-(2-aminoetyl)benzensulfonamid]; [6]; [m-hydroxystilbene]
docking score: -10.168

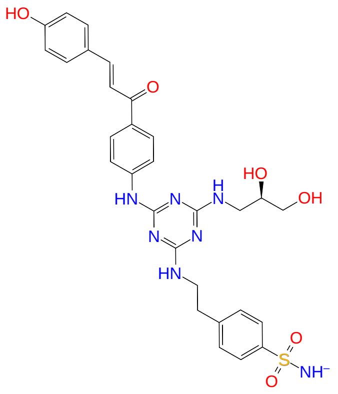

title: kyanurchlorid; [4-(2-aminoetyl)benzensulfonamid]; [3]; [p-hydroxychalcone]
docking score: -10.145

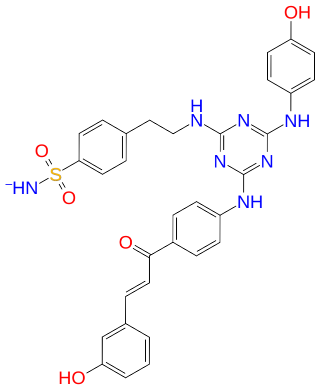

title: kyanurchlorid; [4-(2-aminoetyl)benzensulfonamid]; [6]; [m-hydroxychalcone]
docking score: -10.142

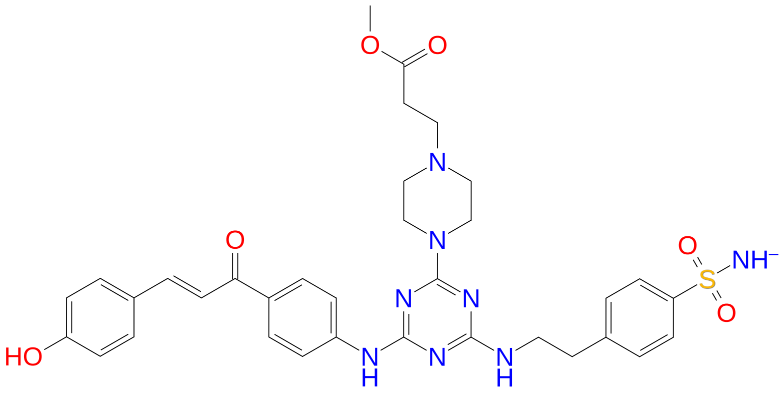

title: kyanurchlorid; [4-(2-aminoetyl)benzensulfonamid]; [11]; [p-hydroxychalcone]
docking score: -10.12

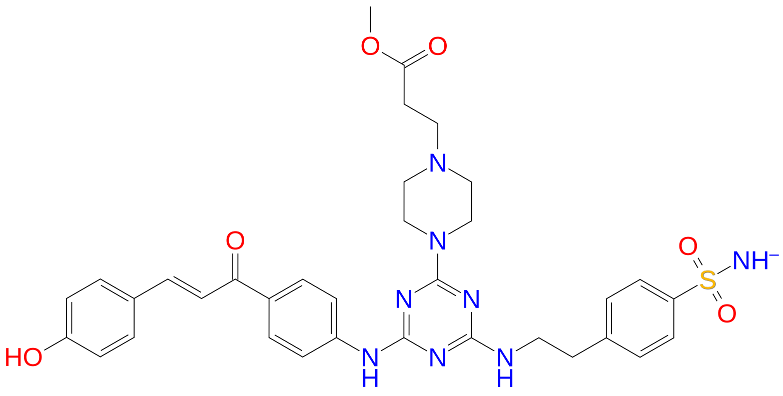

title: kyanurchlorid; [4-(2-aminoetyl)benzensulfonamid]; [11]; [p-hydroxychalcone]
docking score: -10.12

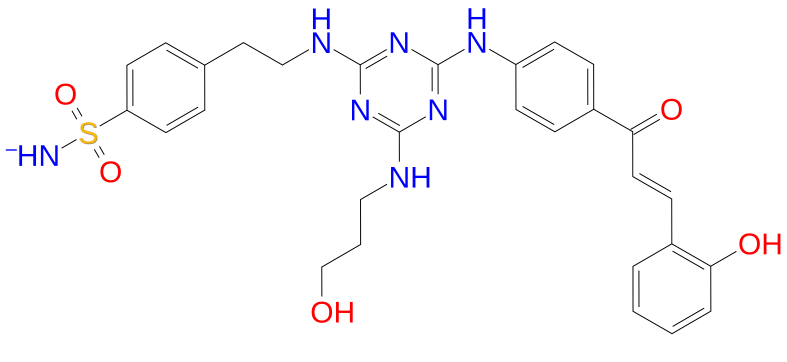

title: kyanurchlorid; [4-(2-aminoetyl)benzensulfonamid]; [2]; [o-hydroxychalcone]
docking score: -9.988

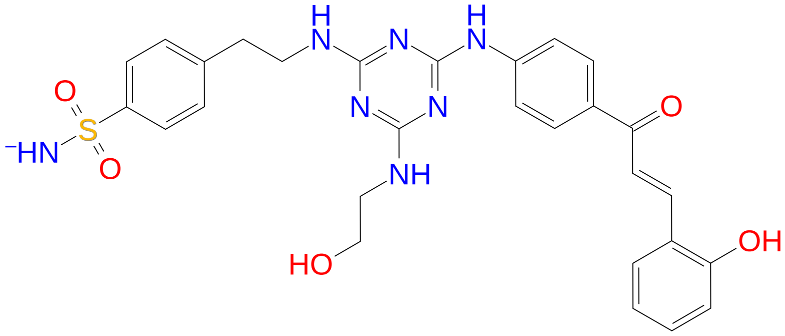

title: kyanurchlorid; [4-(2-aminoetyl)benzensulfonamid]; [1]; [o-hydroxychalcone]
docking score: -9.974

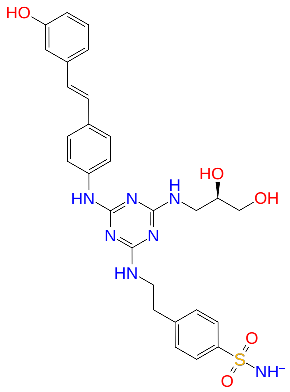

title: kyanurchlorid; [4-(2-aminoetyl)benzensulfonamid]; [3]; [m-hydroxystilbene]
docking score: -9.908

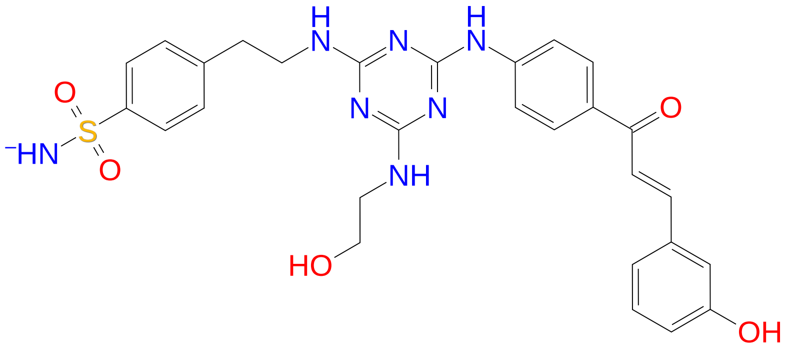

title: kyanurchlorid; [4-(2-aminoetyl)benzensulfonamid]; [1]; [m-hydroxychalcone]
docking score: -9.891

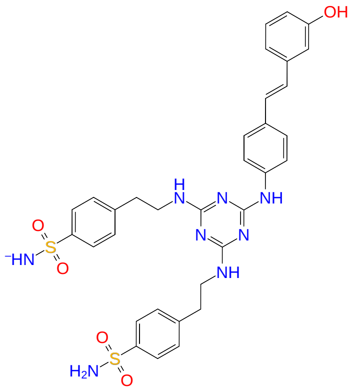

title: kyanurchlorid; [4-(2-aminoetyl)benzensulfonamid]; [4-(2-aminoetyl)benzensulfonamid]; [m-hydroxystilbene]
docking score: -9.733

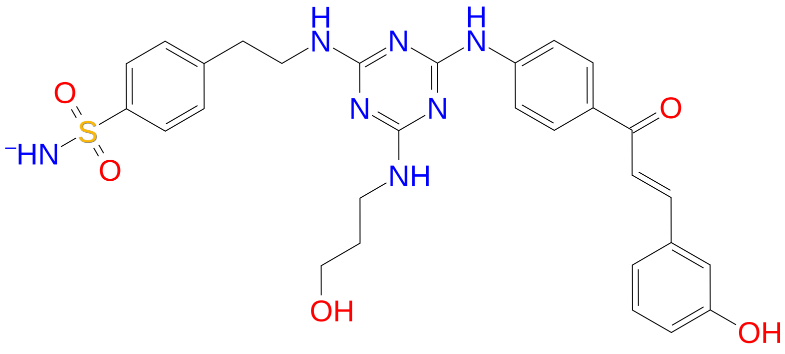

title: kyanurchlorid; [4-(2-aminoetyl)benzensulfonamid]; [2]; [m-hydroxychalcone]
docking score: -9.729

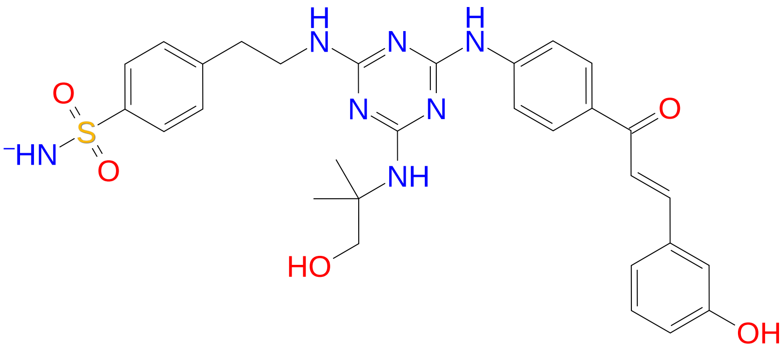

title: kyanurchlorid; [4-(2-aminoetyl)benzensulfonamid]; [5]; [m-hydroxychalcone]
docking score: -9.711

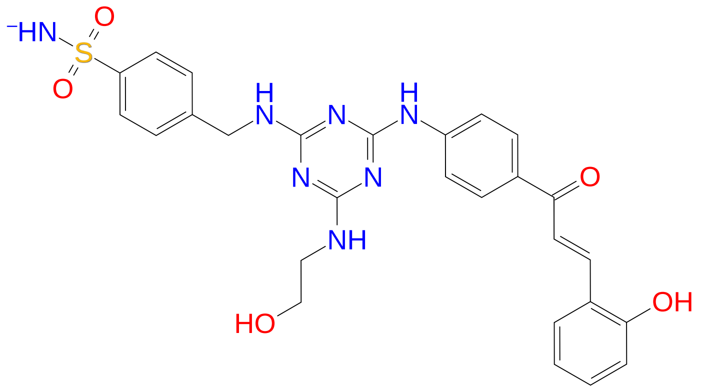

title: kyanurchlorid; [homosulfanilamid]; [1]; [o-hydroxychalcone]
docking score: -9.698

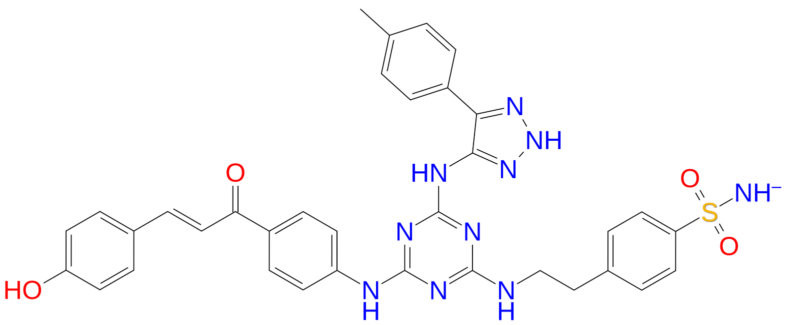

title: kyanurchlorid; [4-(2-aminoetyl)benzensulfonamid]; [17]; [p-hydroxychalcone]
docking score: -9.669

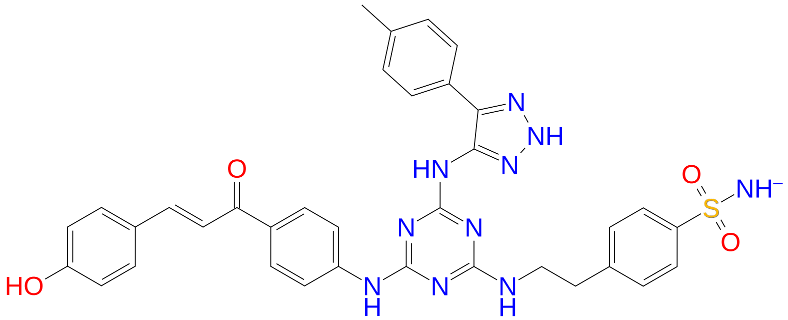

title: kyanurchlorid; [4-(2-aminoetyl)benzensulfonamid]; [17]; [p-hydroxychalcone]
docking score: -9.669

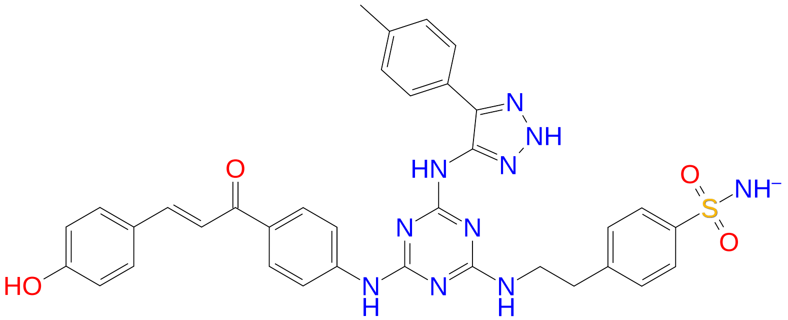

title: kyanurchlorid; [4-(2-aminoetyl)benzensulfonamid]; [17]; [p-hydroxychalcone]
docking score: -9.669

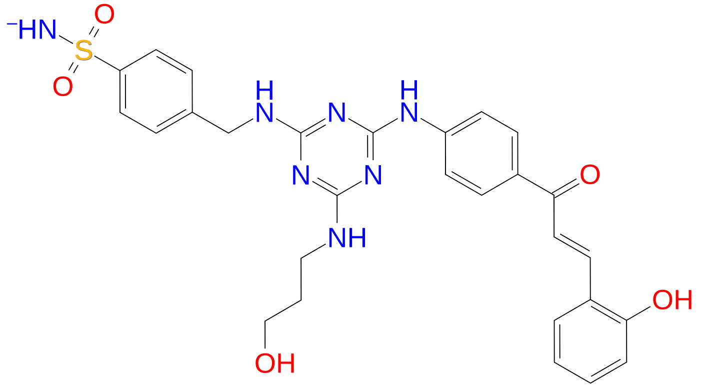

title: kyanurchlorid; [homosulfanilamid]; [2]; [o-hydroxychalcone]
docking score: -9.626

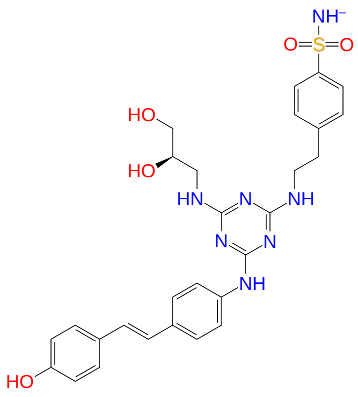

title: kyanurchlorid; [4-(2-aminoetyl)benzensulfonamid]; [3]; [p-hydroxystilbene]
docking score: -9.605

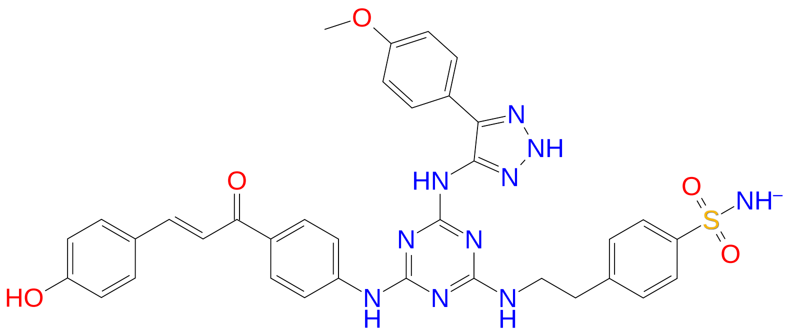

title: kyanurchlorid; [4-(2-aminoetyl)benzensulfonamid]; [16]; [p-hydroxychalcone]
docking score: -9.572

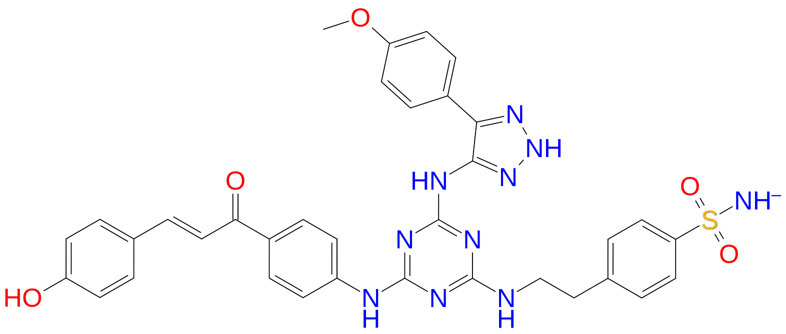

title: kyanurchlorid; [4-(2-aminoetyl)benzensulfonamid]; [16]; [p-hydroxychalcone]
docking score: -9.572

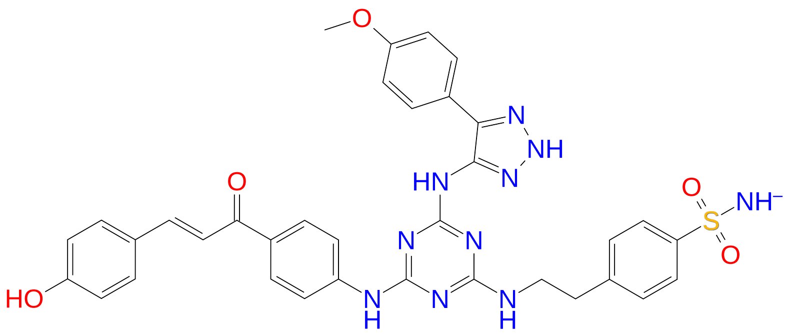

title: kyanurchlorid; [4-(2-aminoetyl)benzensulfonamid]; [16]; [p-hydroxychalcone]
docking score: -9.572

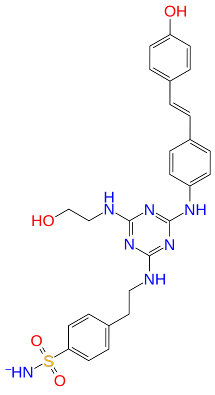

title: kyanurchlorid; [4-(2-aminoetyl)benzensulfonamid]; [1]; [p-hydroxystilbene]
docking score: -9.537

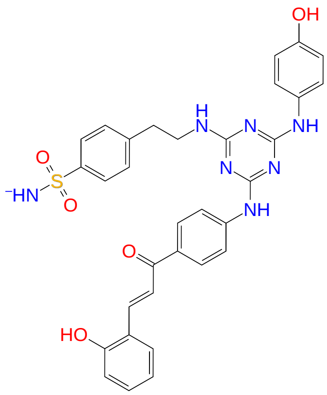

title: kyanurchlorid; [4-(2-aminoetyl)benzensulfonamid]; [6]; [o-hydroxychalcone]
docking score: -9.519

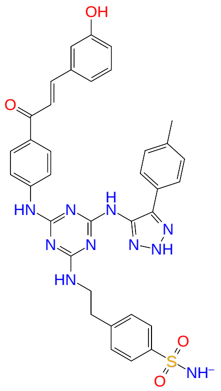

title: kyanurchlorid; [4-(2-aminoetyl)benzensulfonamid]; [17]; [m-hydroxychalcone]
docking score: -9.49

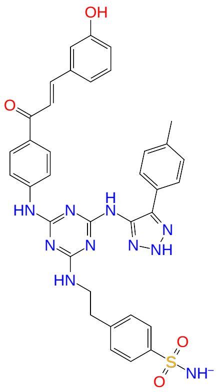

title: kyanurchlorid; [4-(2-aminoetyl)benzensulfonamid]; [17]; [m-hydroxychalcone]
docking score: -9.49

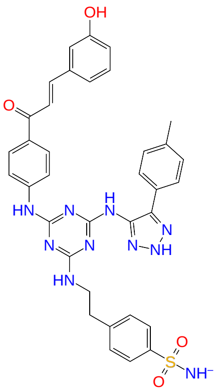

title: kyanurchlorid; [4-(2-aminoetyl)benzensulfonamid]; [17]; [m-hydroxychalcone]
docking score: -9.49

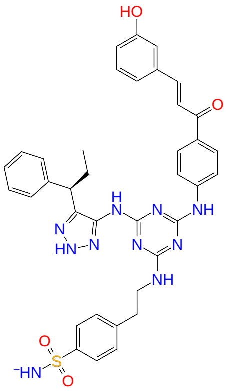

title: kyanurchlorid; [4-(2-aminoetyl)benzensulfonamid]; [21]; [m-hydroxychalcone]
docking score: -9.444

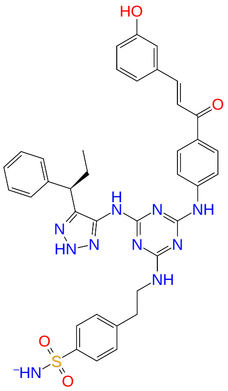

title: kyanurchlorid; [4-(2-aminoetyl)benzensulfonamid]; [21]; [m-hydroxychalcone]
docking score: -9.444

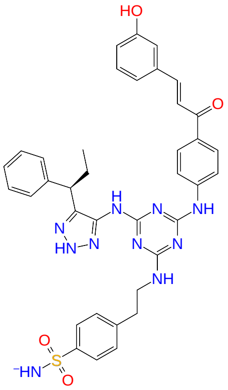

title: kyanurchlorid; [4-(2-aminoetyl)benzensulfonamid]; [21]; [m-hydroxychalcone]
docking score: -9.444

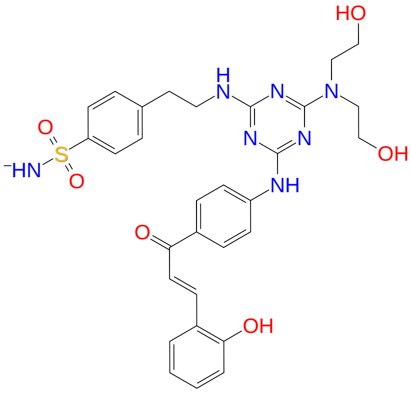

title: kyanurchlorid; [4-(2-aminoetyl)benzensulfonamid]; [4]; [o-hydroxychalcone]
docking score: -9.438

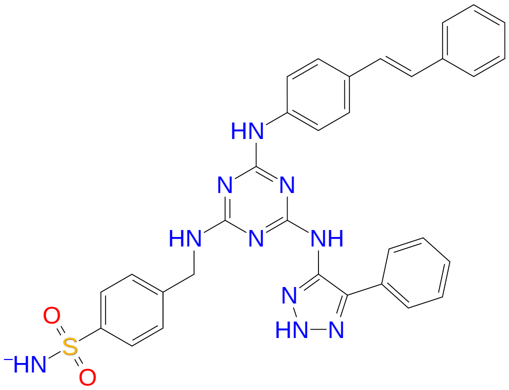

title: kyanurchlorid; [homosulfanilamid]; [20]; [stilbene]
docking score: -9.415

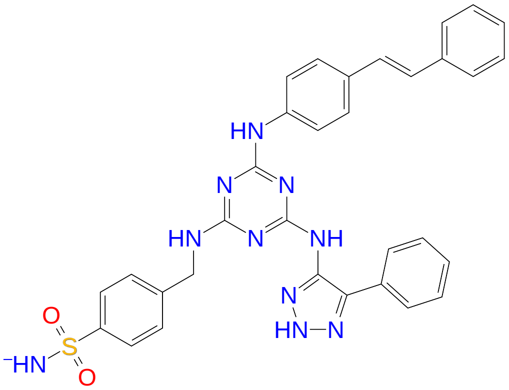

title: kyanurchlorid; [homosulfanilamid]; [20]; [stilbene]
docking score: -9.415

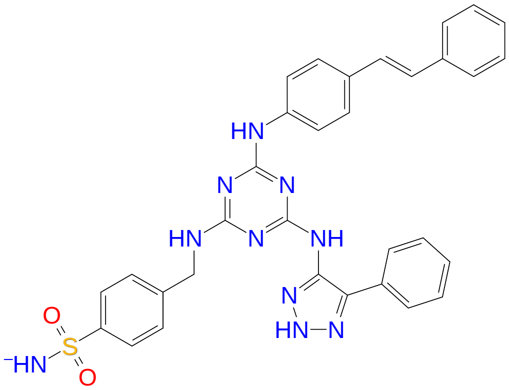

title: kyanurchlorid; [homosulfanilamid]; [20]; [stilbene]
docking score: -9.415

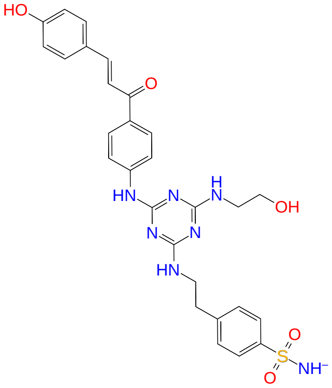

title: kyanurchlorid; [4-(2-aminoetyl)benzensulfonamid]; [1]; [p-hydroxychalcone]
docking score: -9.396

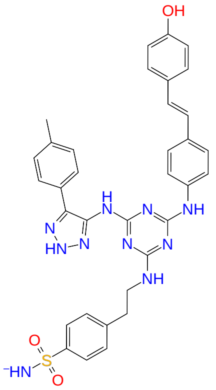

title: kyanurchlorid; [4-(2-aminoetyl)benzensulfonamid]; [17]; [p-hydroxystilbene]
docking score: -9.339

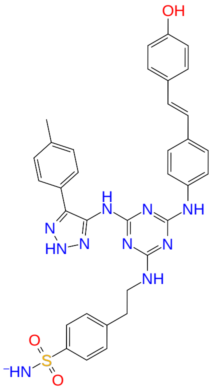

title: kyanurchlorid; [4-(2-aminoetyl)benzensulfonamid]; [17]; [p-hydroxystilbene]
docking score: -9.339

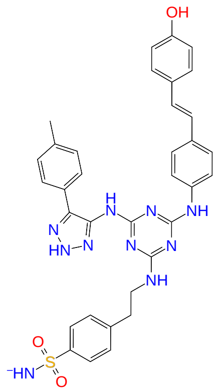

title: kyanurchlorid; [4-(2-aminoetyl)benzensulfonamid]; [17]; [p-hydroxystilbene]
docking score: -9.339

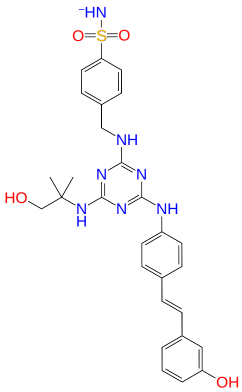

title: kyanurchlorid; [homosulfanilamid]; [5]; [m-hydroxystilbene]
docking score: -9.126

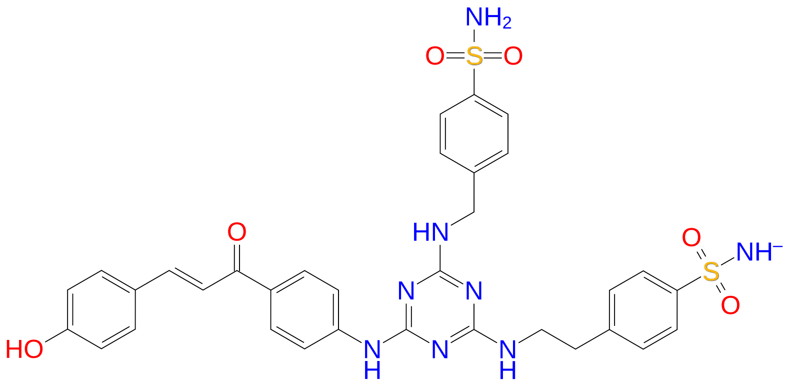

title: kyanurchlorid; [4-(2-aminoetyl)benzensulfonamid]; [homosulfanilamid]; [p-hydroxychalcone]
docking score: -9.093

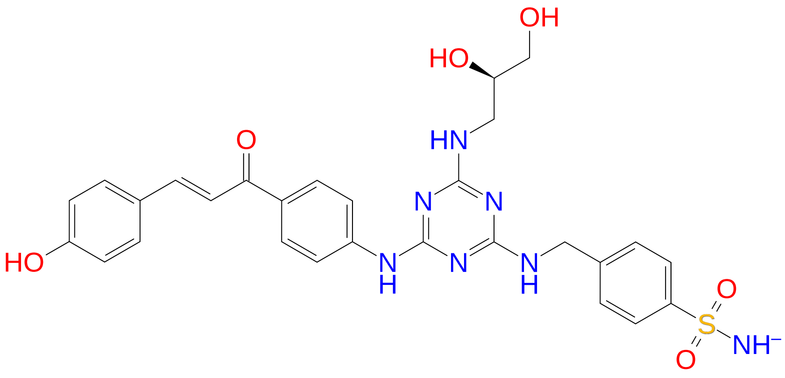

title: kyanurchlorid; [homosulfanilamid]; [3]; [p-hydroxychalcone]
docking score: -9.028

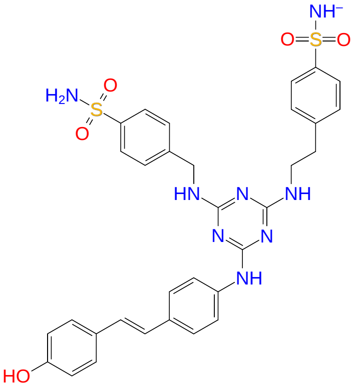

title: kyanurchlorid; [4-(2-aminoetyl)benzensulfonamid]; [homosulfanilamid]; [p-hydroxystilbene]
docking score: -8.981

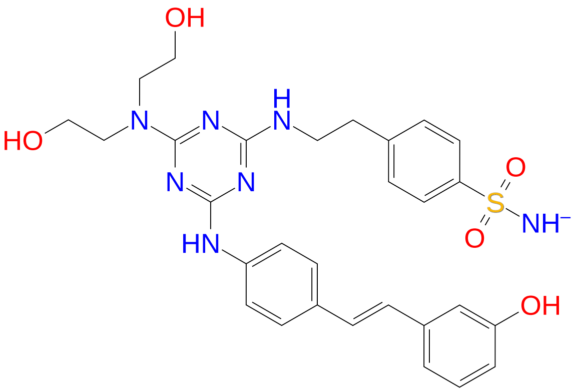

title: kyanurchlorid; [4-(2-aminoetyl)benzensulfonamid]; [4]; [m-hydroxystilbene]
docking score: -8.917

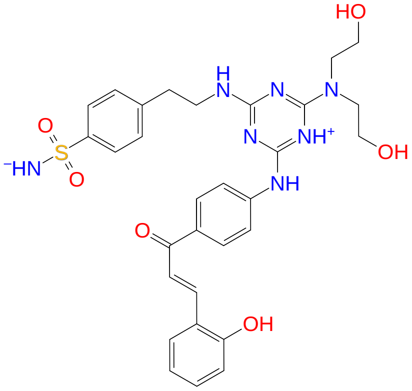

title: kyanurchlorid; [4-(2-aminoetyl)benzensulfonamid]; [4]; [o-hydroxychalcone]
docking score: -8.86

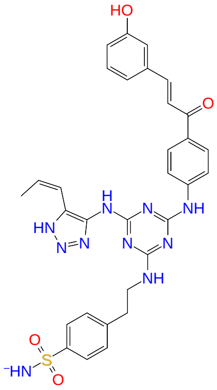

title: kyanurchlorid; [4-(2-aminoetyl)benzensulfonamid]; [18]; [m-hydroxychalcone]
docking score: -8.817

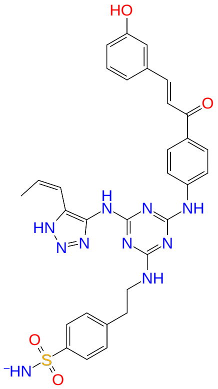

title: kyanurchlorid; [4-(2-aminoetyl)benzensulfonamid]; [18]; [m-hydroxychalcone]
docking score: -8.817

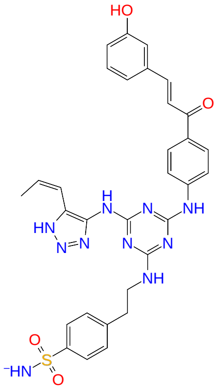

title: kyanurchlorid; [4-(2-aminoetyl)benzensulfonamid]; [18]; [m-hydroxychalcone]
docking score: -8.817

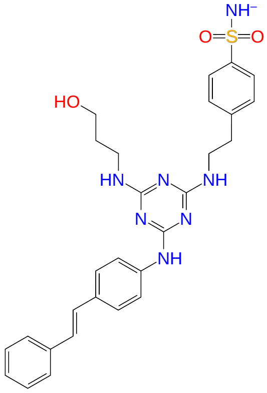

title: kyanurchlorid; [4-(2-aminoetyl)benzensulfonamid]; [2]; [stilbene]
docking score: -8.813

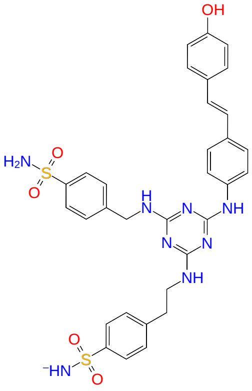

title: kyanurchlorid; [homosulfanilamid]; [4-(2-aminoetyl)benzensulfonamid]; [p-hydroxystilbene]
docking score: -8.649

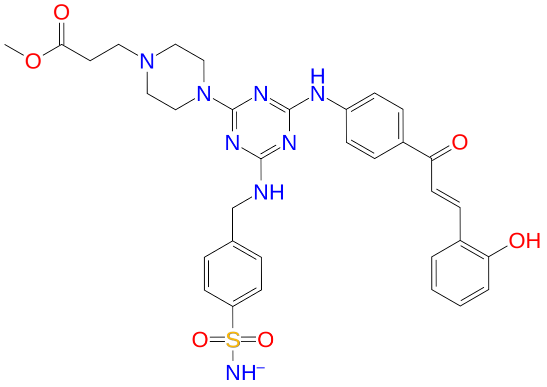

title: kyanurchlorid; [homosulfanilamid]; [11]; [o-hydroxychalcone]
docking score: -8.646

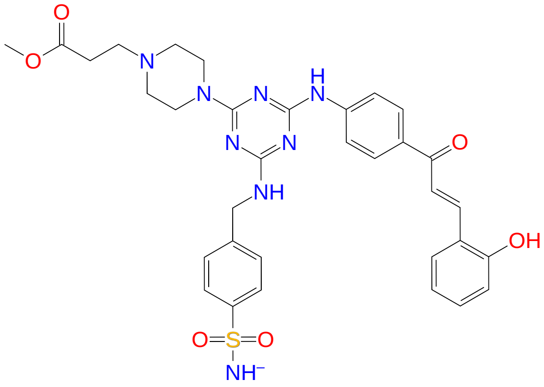

title: kyanurchlorid; [homosulfanilamid]; [11]; [o-hydroxychalcone]
docking score: -8.646

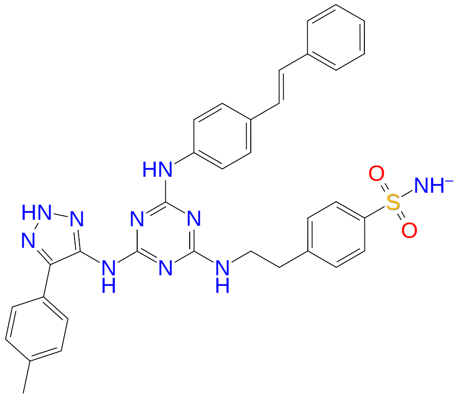

title: kyanurchlorid; [4-(2-aminoetyl)benzensulfonamid]; [17]; [stilbene]
docking score: -8.634

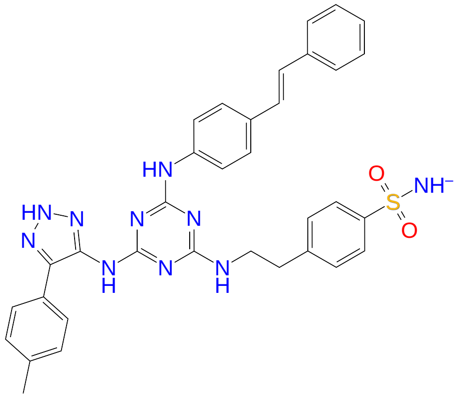

title: kyanurchlorid; [4-(2-aminoetyl)benzensulfonamid]; [17]; [stilbene]
docking score: -8.634

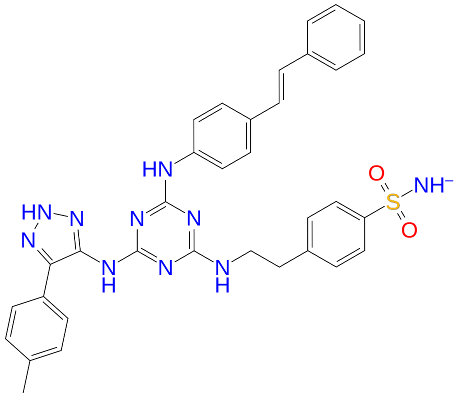

title: kyanurchlorid; [4-(2-aminoetyl)benzensulfonamid]; [17]; [stilbene]
docking score: -8.634

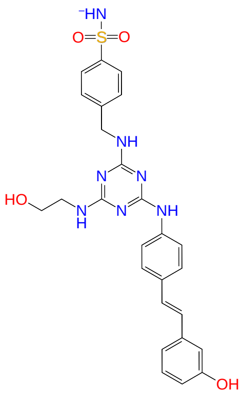

title: kyanurchlorid; [homosulfanilamid]; [1]; [m-hydroxystilbene]
docking score: -8.539

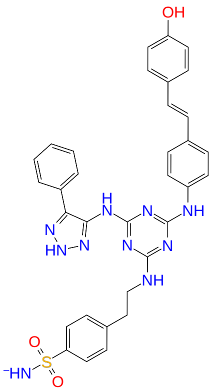

title: kyanurchlorid; [4-(2-aminoetyl)benzensulfonamid]; [20]; [p-hydroxystilbene]
docking score: -8.414

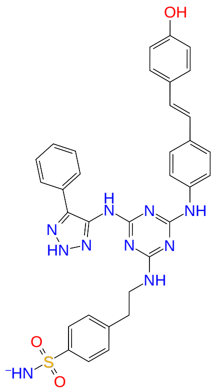

title: kyanurchlorid; [4-(2-aminoetyl)benzensulfonamid]; [20]; [p-hydroxystilbene]
docking score: -8.414

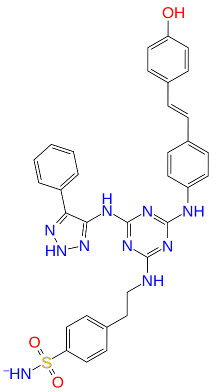

title: kyanurchlorid; [4-(2-aminoetyl)benzensulfonamid]; [20]; [p-hydroxystilbene]
docking score: -8.414

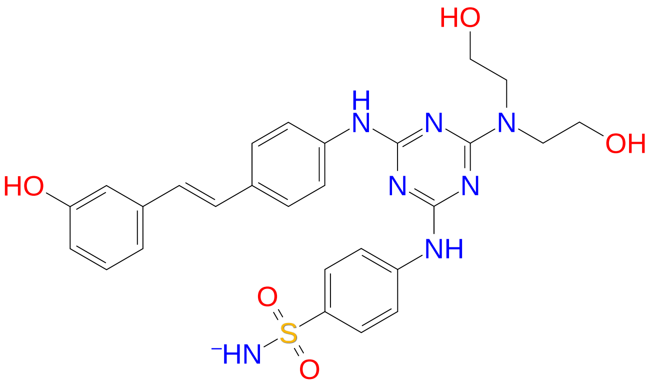

title: kyanurchlorid; [sulfanilamid]; [4]; [m-hydroxystilbene]
docking score: -8.246

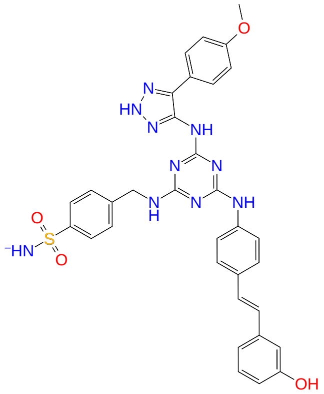

title: kyanurchlorid; [homosulfanilamid]; [16]; [m-hydroxystilbene]
docking score: -8.194

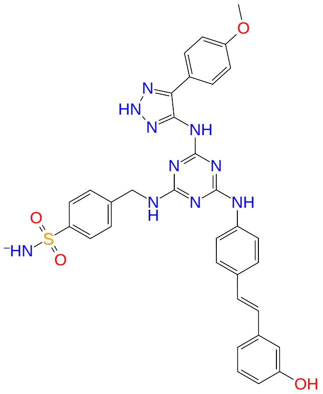

title: kyanurchlorid; [homosulfanilamid]; [16]; [m-hydroxystilbene]
docking score: -8.194

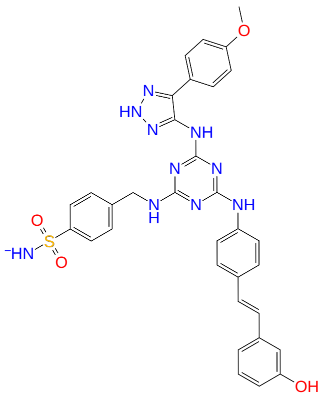

title: kyanurchlorid; [homosulfanilamid]; [16]; [m-hydroxystilbene]
docking score: -8.194

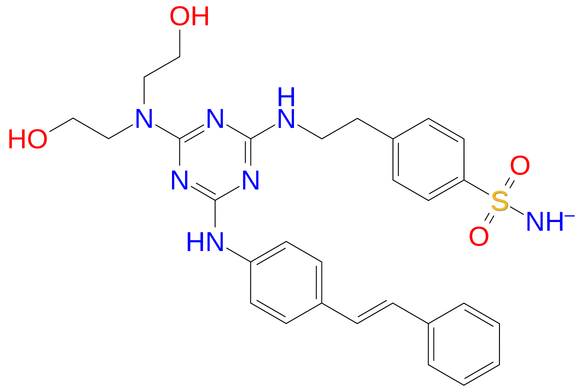

title: kyanurchlorid; [4-(2-aminoetyl)benzensulfonamid]; [4]; [stilbene]
docking score: -8.172

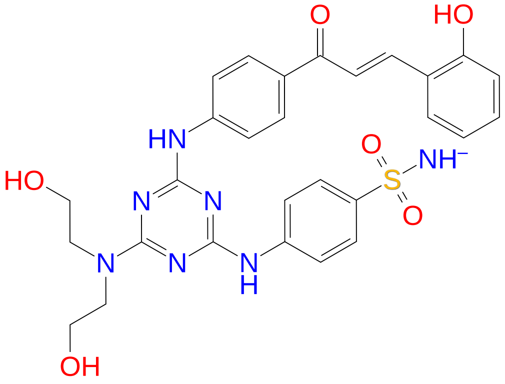

title: kyanurchlorid; [sulfanilamid]; [4]; [o-hydroxychalcone]
docking score: -8.113

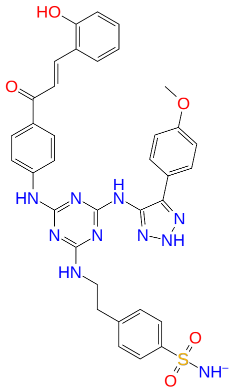

title: kyanurchlorid; [4-(2-aminoetyl)benzensulfonamid]; [16]; [o-hydroxychalcone]
docking score: -7.462

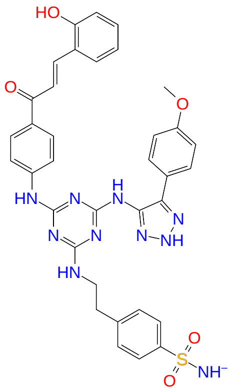

title: kyanurchlorid; [4-(2-aminoetyl)benzensulfonamid]; [16]; [o-hydroxychalcone]
docking score: -7.462

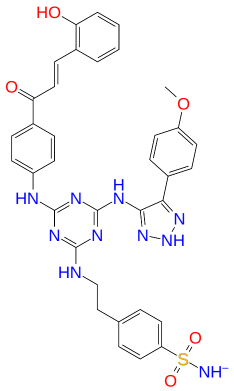

title: kyanurchlorid; [4-(2-aminoetyl)benzensulfonamid]; [16]; [o-hydroxychalcone]
docking score: -7.462

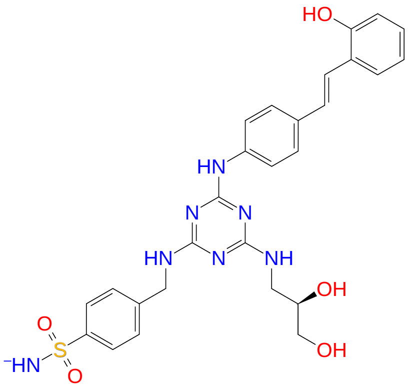

title: kyanurchlorid; [homosulfanilamid]; [3]; [o-hydroxystilbene]
docking score: -4.241

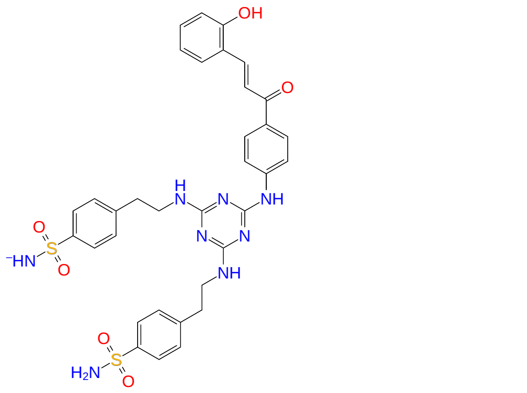

title: kyanurchlorid; [4-(2-aminoetyl)benzensulfonamid]; [4-(2-aminoetyl)benzensulfonamid]; [o-hydroxychalcone]
docking score: -3.988

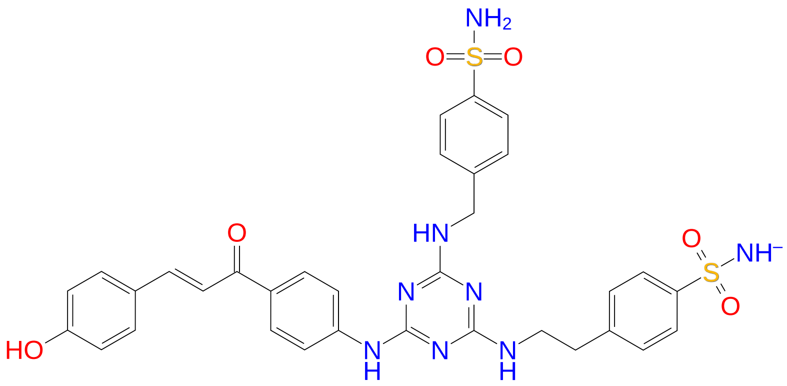

title: kyanurchlorid; [homosulfanilamid]; [4-(2-aminoetyl)benzensulfonamid]; [p-hydroxychalcone]
docking score: -3.054

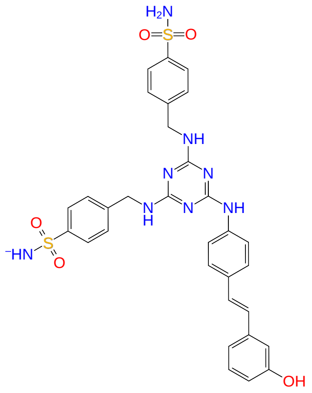

title: kyanurchlorid; [homosulfanilamid]; [homosulfanilamid]; [m-hydroxystilbene]
docking score: -2.52
