# Supplementary material for: Novel 1,3,5-Triazinyl Aminobenzenesulfonamides Incorporating Aminoalcohol, Aminochalcone and Aminostilbene Structural Motifs as Potent Anti-VRE Agents, and Carbonic Anhydrases I, II, VII, IX, and XII Inhibitors
Source: Int J Mol Sci. 2021 Dec 26;23(1):231. doi: 10.3390/ijms23010231 (PMC8745223; doi:10.3390/ijms23010231)
Supplement: Supplementary file 1 [file ijms-23-00231-s001.zip › Supplemetary information.pdf]

Supplementary information

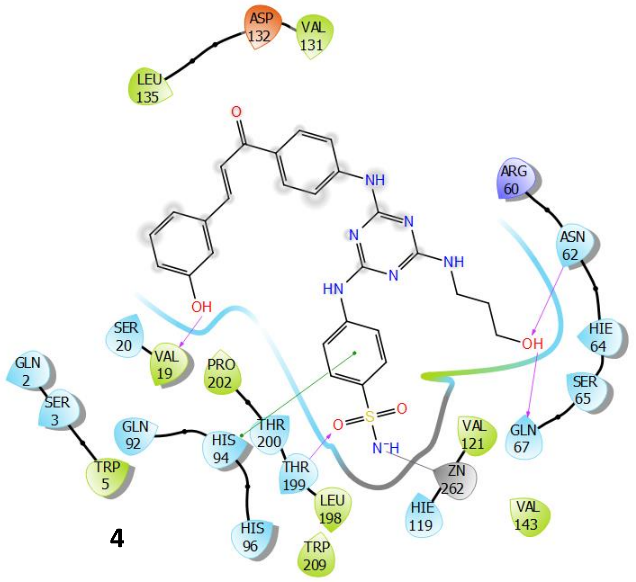

4

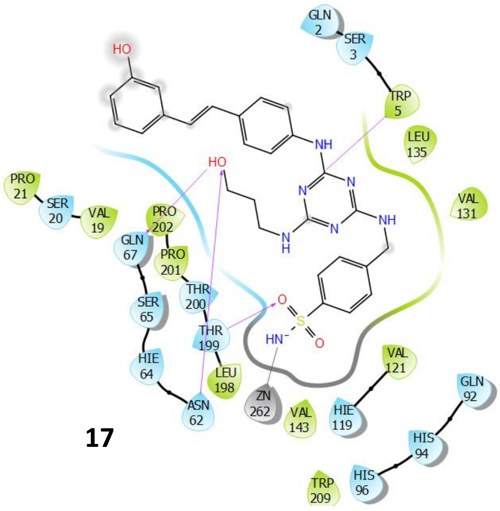

17

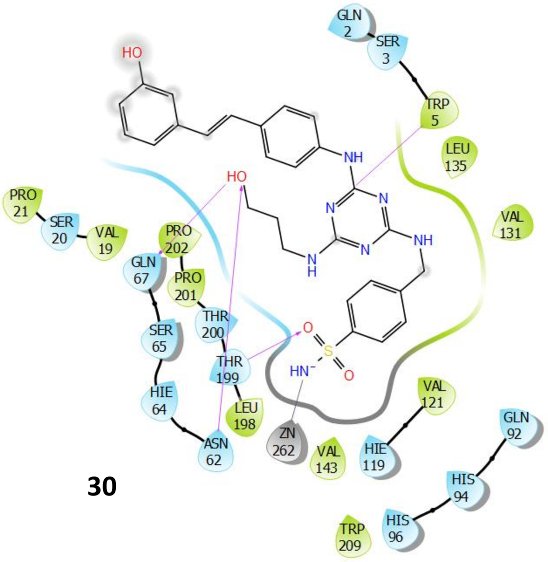

30

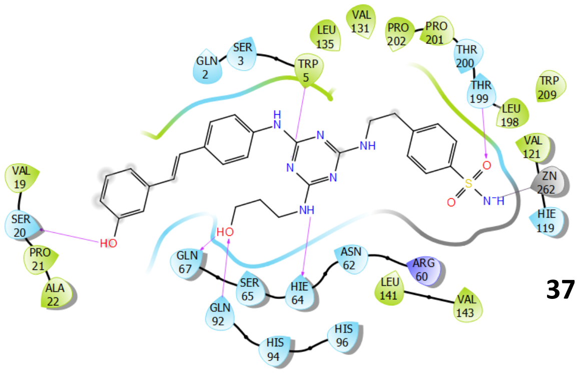

37

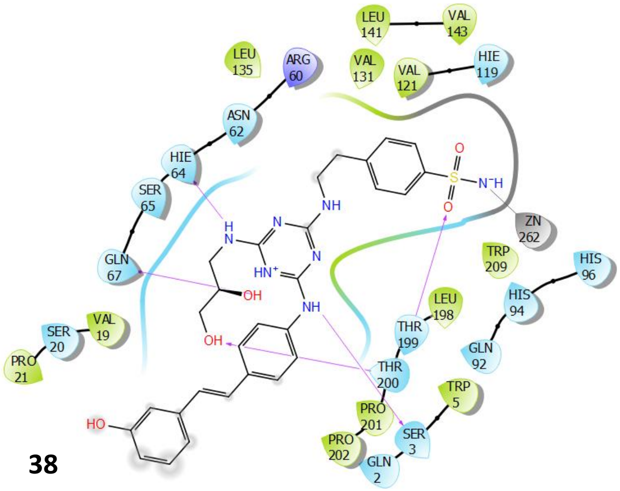

38

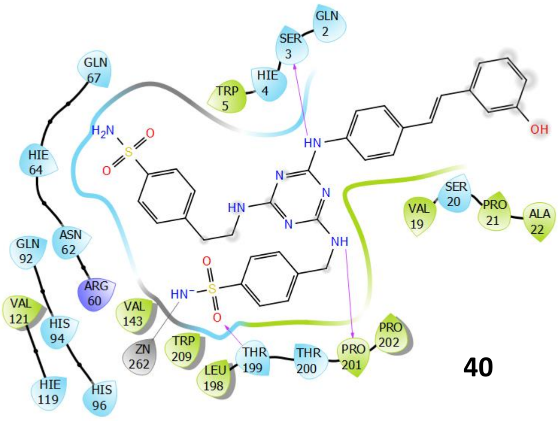

40

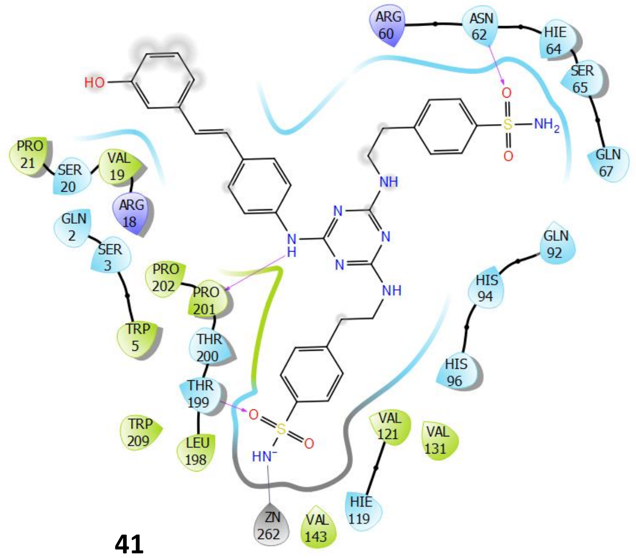

41

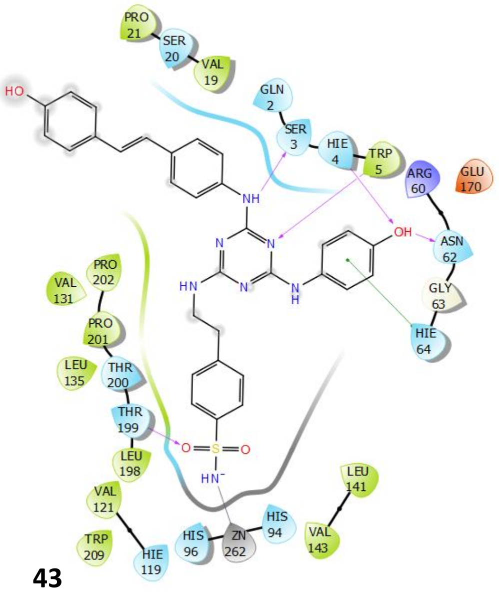

43

Suppl. Inf. Figure 1. 2D interaction diagrams of ligands docked in hCA IX.

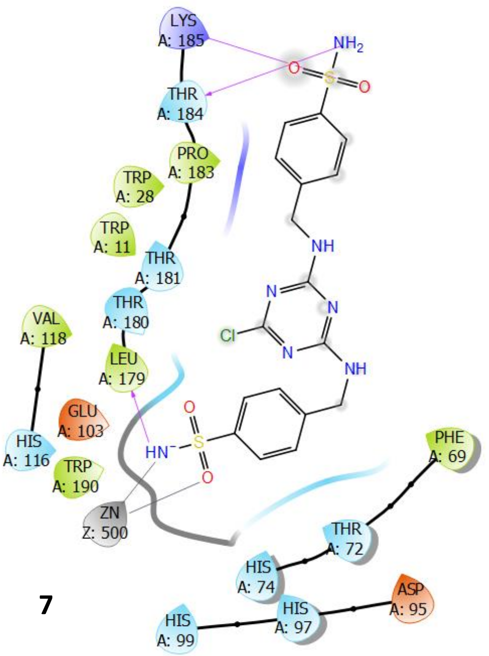
LYS A: 185
THR A: 184
PRO A: 183
TRP A: 28
TRP A: 11
THR A: 181
THR A: 180
VAL A: 118
LEU A: 179
GLU A: 103
HIS A: 116
TRP A: 190
ZN Z: 500
PHE A: 69
THR A: 72
HIS A: 74
HIS A: 99
HIS A: 97
ASP A: 95
NH2
O
S
NH
N
Cl
HN⁻

7

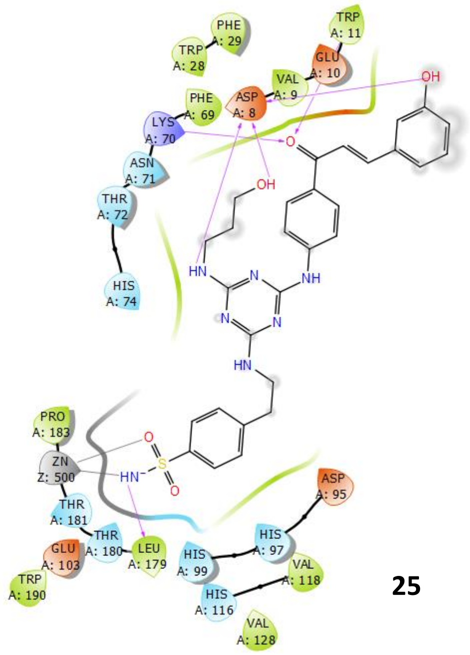
PHE A: 29
TRP A: 28
TRP A: 11
GLU A: 10
VAL A: 9
PHE A: 69
ASP A: 8
LYS A: 70
ASN A: 71
THR A: 72
HIS A: 74
OH
O
HN
NH
N
PRO A: 183
ZN Z: 500
HN⁻
THR A: 181
THR A: 180
LEU A: 179
GLU A: 103
TRP A: 190
ASP A: 95
HIS A: 97
HIS A: 99
VAL A: 118
HIS A: 116
VAL A: 128

25

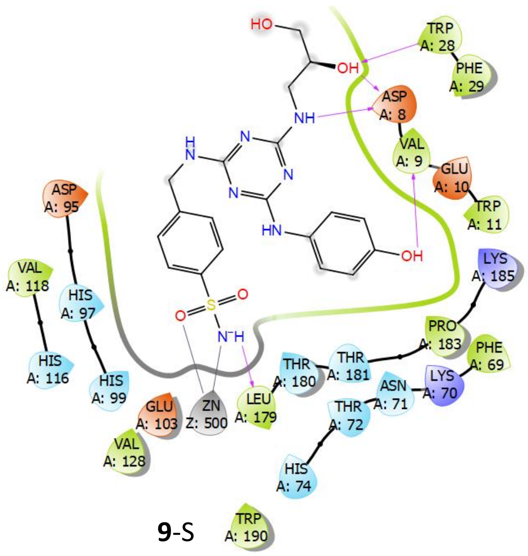
HO
OH
TRP A: 28
PHE A: 29
ASP A: 8
NH
VAL A: 9
GLU A: 10
TRP A: 11
HN
OH
ASP A: 95
VAL A: 118
HIS A: 97
HIS A: 116
HIS A: 99
LYS A: 185
PRO A: 183
PHE A: 69
THR A: 180
THR A: 181
ASN A: 71
LYS A: 70
THR A: 72
GLU A: 103
ZN Z: 500
LEU A: 179
VAL A: 128
HIS A: 74
TRP A: 190

9-S

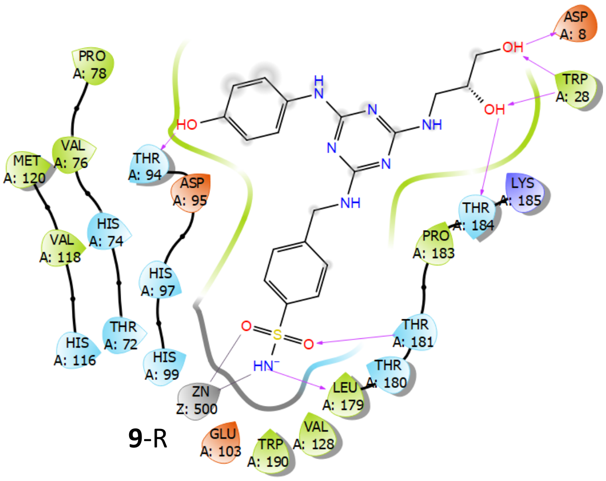
PRO A: 78
VAL A: 76
MET A: 120
THR A: 94
HO
ASP A: 95
HIS A: 74
VAL A: 118
HIS A: 97
HIS A: 116
THR A: 72
HIS A: 99
ZN Z: 500
NH
OH
ASP A: 8
TRP A: 28
LYS A: 185
THR A: 184
PRO A: 183
THR A: 181
THR A: 180
LEU A: 179
HN⁻
GLU A: 103
TRP A: 190
VAL A: 128

9-R

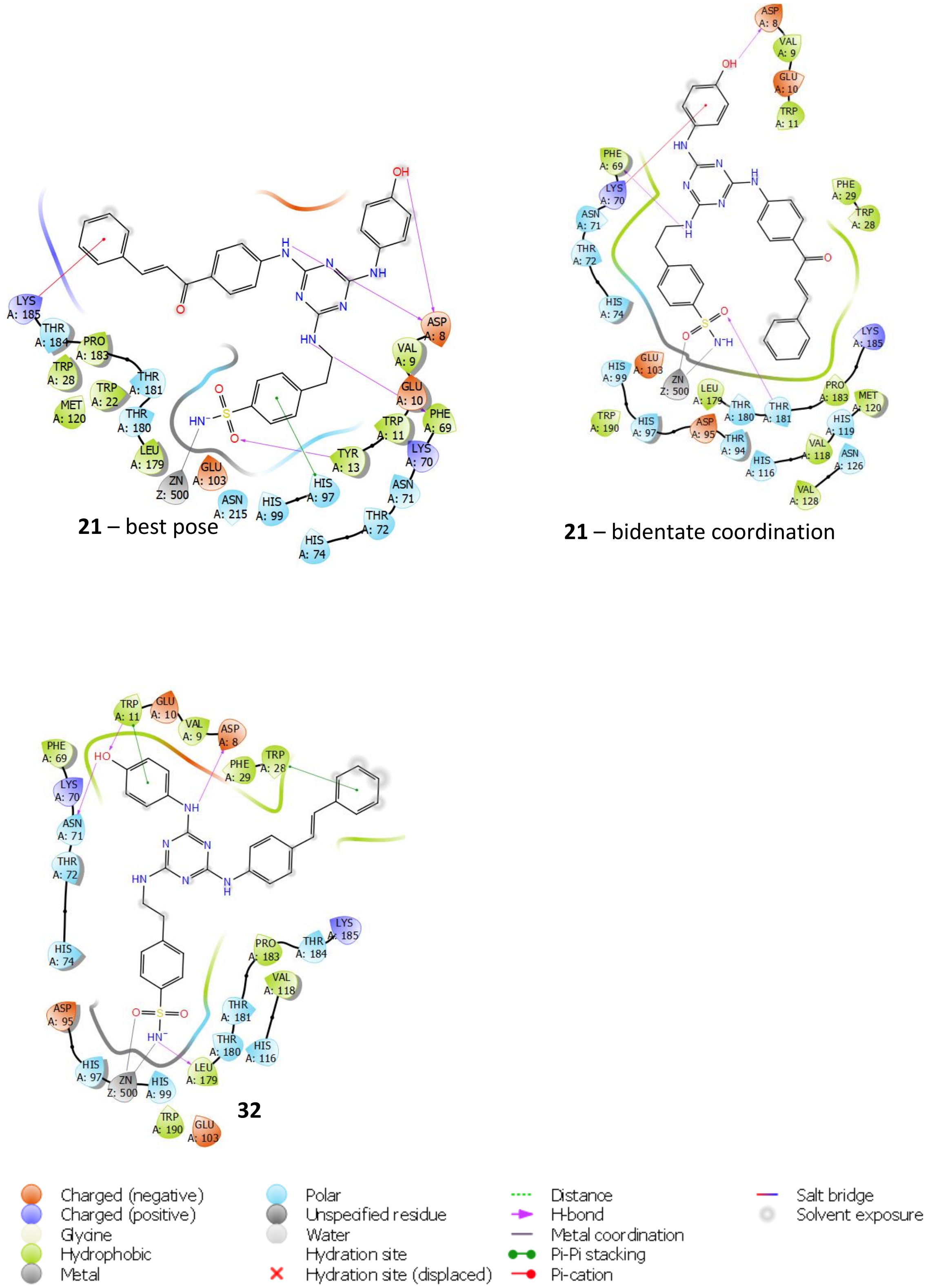

Suppl. Inf. Figure 2. 2D interaction diagrams of ligands docked in enterococcal CA.

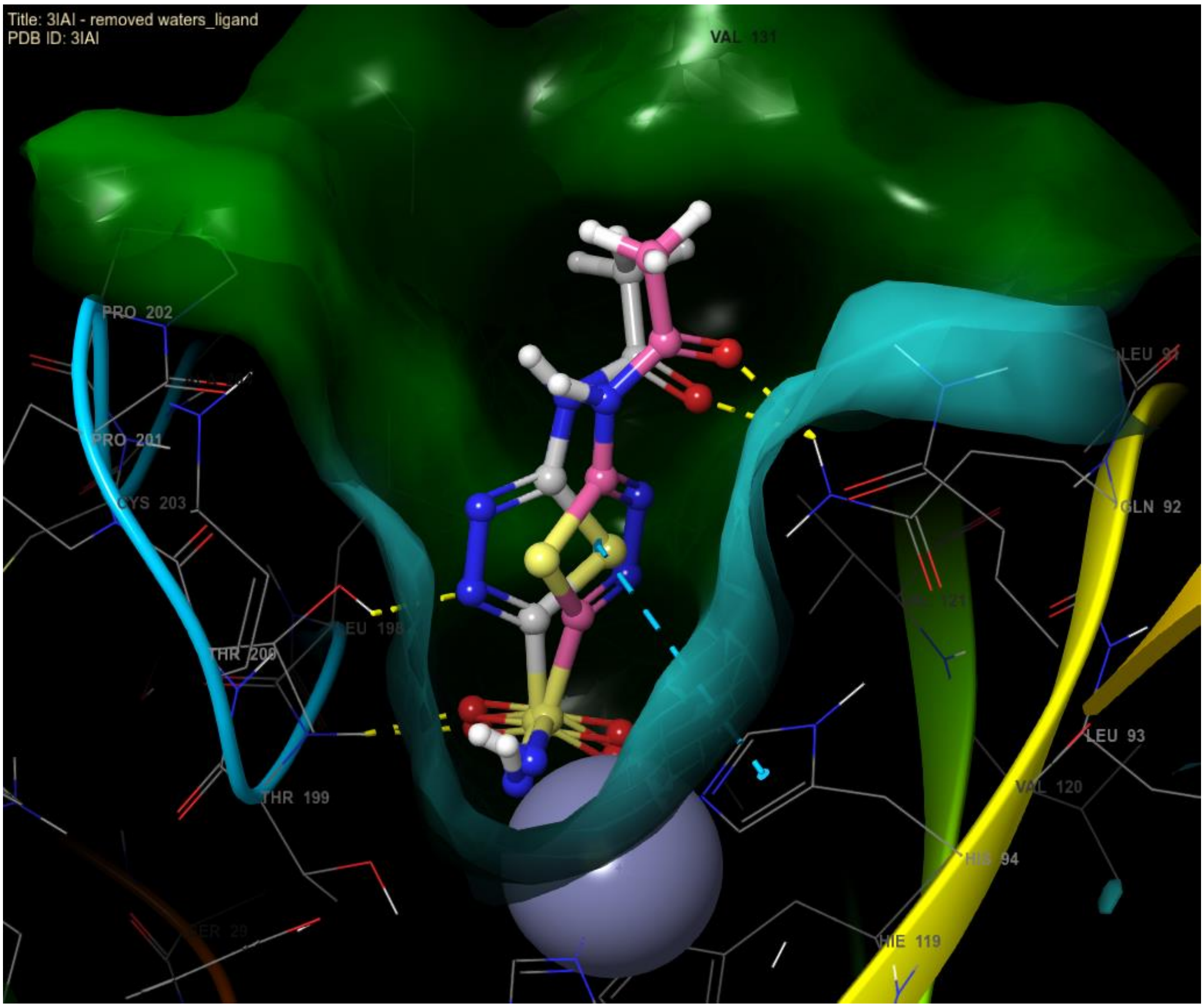

Suppl. Inf. Figure 3. Superposition of acetazolamide co-crystallized with hCA IX (the molecule with light gray carbons) and pose of acetazolamide docked in hCA IX (the molecule with pink carbons). RMSD = 1.57 Å^2

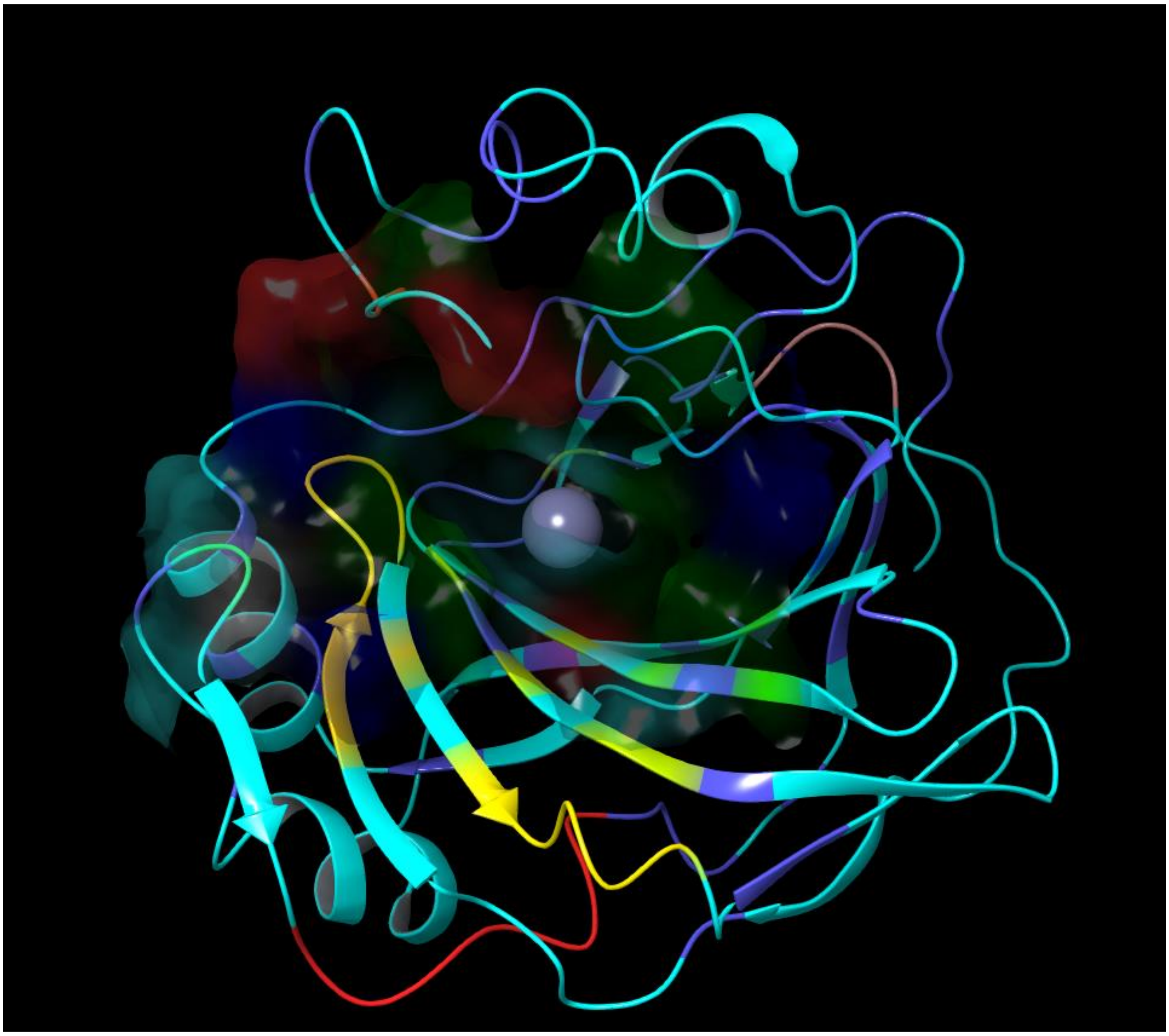

Suppl. Inf. Figure 4. 3D representation of enterococcal carbonic anhydrase created by homology modeling. Protein is depicted by ribbon and surface area of binding site with zinc dication at the bottom.
