# Supplementary figures and images for: Novel 1,3,5-Triazinyl Aminobenzenesulfonamides Incorporating Aminoalcohol, Aminochalcone and Aminostilbene Structural Motifs as Potent Anti-VRE Agents, and Carbonic Anhydrases I, II, VII, IX, and XII Inhibitors
Source: Int J Mol Sci. 2021 Dec 26;23(1):231. doi: 10.3390/ijms23010231 (PMC8745223; doi:10.3390/ijms23010231)

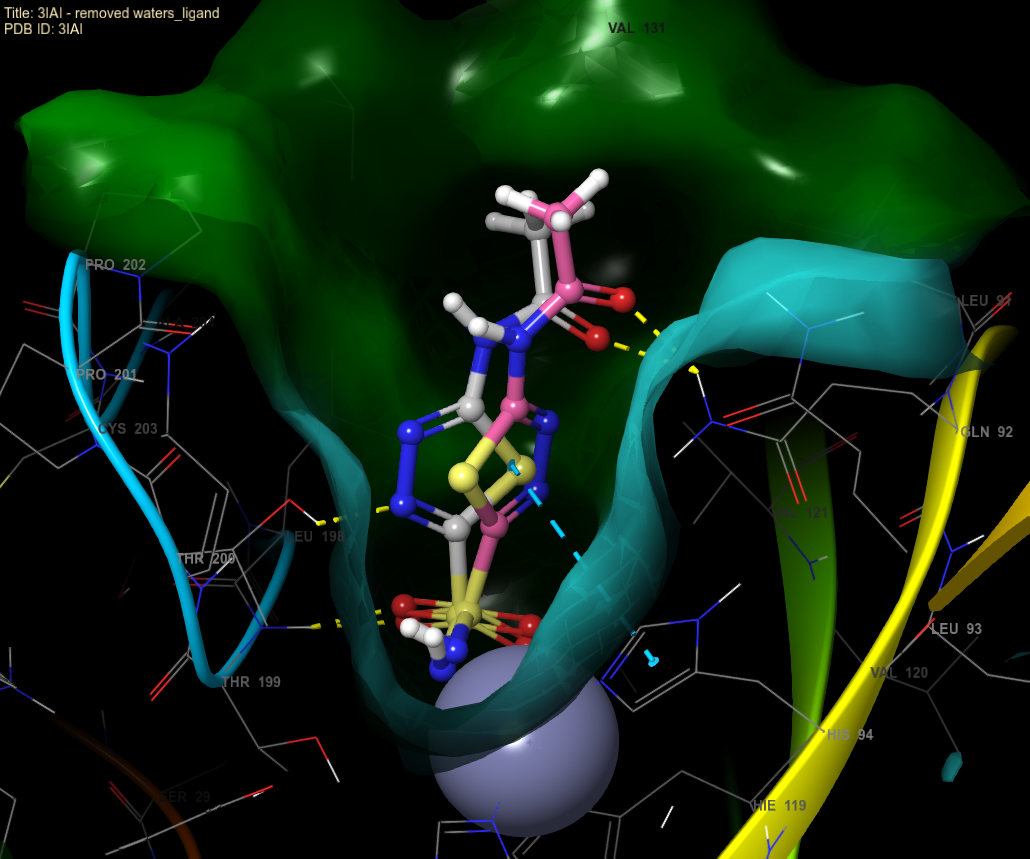

Supplement: Supplementary file 1 [file ijms-23-00231-s001.zip › Suppl. inf. Fig. 2 CAIX-acetazolamide - validation.png]
